# Supplementary material for: Head Injury Evaluation and Ambulance Diagnosis (HOME) Study protocol: a feasibility study assessing the implementation of the Canadian CT Head Rule in the prehospital setting
Source: BMJ Open. 2024 Jun 11;14(6):e077191. doi: 10.1136/bmjopen-2023-077191 (PMC11168128; doi:10.1136/bmjopen-2023-077191)
Supplement: Supplementary data [file bmjopen-2023-077191supp001.pdf]

HOME STUDY

**Head injury Evaluation and Ambulance Diagnosis: A Feasibility Study**

**Semi-structured interview, topic guide – Patients**

**Date:** **Participant ID number:**

**Pre-interview**

- Privacy notice read.
- Consent form.
- Information sheet.

---

1. Please share your experience from the moment you were initially approached about the study.
   - **Follow-up questions, if required:**

   a. How did you feel about being approached for the study? Were there any specific factors that influenced your decision to participate?
   b. Were you given enough information to help you to decide whether to take part in this study? Was there anything that you found confusing or unclear?
   c. In this study, we didn't ask patients for permission during the ambulance journey. This is because they had experienced a head injury and were being taken to hospital as an emergency. How did you feel about this? Do you think you would have been able to make a decision about taking part when you were still in the ambulance?

2. How did you feel about the way we asked for your permission to take part in this study?
   - **Follow-up questions, if required:**

   a. Please share your overall thoughts about how we asked for your permission to take part in this study.
   b. Is there anything particularly good or bad about the way we asked for your permission to take part in the study?
   c. Do you think you were given enough time to decide whether to take part? Why or why not?

3. Can you recall any specific reasons or factors that influenced your belief that it is important to understand the study? How did this understanding affect your decision to take part in this study?



Alqurashi N, *et al. BMJ Open* 2024; 14:e077191. doi: 10.1136/bmjopen-2023-077191

4. In your opinion, are there any factors that you believe might discourage people from participating in this study?

**Follow-up questions, if required:**

a. Is there anything that might discourage people from participating in this study? How do you think these factors could be addressed?

5. Can you share any unexpected events or experiences that occurred during your participation in the study?

6. Would you recommend taking part in this trial to somebody else?

**Follow-up questions, if required:**

a. Can you explain your thoughts and the reasons behind your recommendation or non-recommendation of participation in this study to others?
b. In your opinion, what specific factors or aspects of the study influenced your recommendation or non-recommendation?
c. Is there anything that you think would stop people from fully participating in this study?

7. Is there anything else you would still like to know about this study?

***END***



Alqurashi N, *et al. BMJ Open* 2024; 14:e077191. doi: 10.1136/bmjopen-2023-077191

**Head injury Evaluation and Ambulance Diagnosis: A Feasibility Study**

**Semi-structured interview, topic guide - <u>Paramedics</u>**

**Date:** **Participant ID number:**

**Pre-interview**

- Privacy notice read.
- Consent form.
- Information sheet.

---

1. Firstly, I would like to know how long you have been a paramedic, and what is your highest academic qualification level?

2. Have you completed the training on the study protocol?
   a. How did you find it?
   b. Did you find the study protocol easy to understand?
   c. Was the protocol easy to remember in practice?
   d. What could be improved in the trial protocol?
3. What was the best aspect of being involved in the study?
   a. Could you tell me how easy it was to use the study case report form?

4. What would be the advantages of using the Canadian CT head Rule in the prehospital setting?
   a. Looking back to your experience, could you tell me how did you find using the Canadian CT head rule in the prehospital field?

5. What would be the disadvantages of using the Canadian CT head Rule in the prehospital setting?
   a. Looking back to your experience, did you experience any barriers/challenges to applying the Canadian CT Head Rule?

6. Would you like the Canadian CT Head Rule to become part of your practice?

7. Do you have any additional thoughts you would like to share about the study?

***END***



Alqurashi N, *et al. BMJ Open* 2024; 14:e077191. doi: 10.1136/bmjopen-2023-077191
