# Supplementary material for: Head Injury Evaluation and Ambulance Diagnosis (HOME) Study protocol: a feasibility study assessing the implementation of the Canadian CT Head Rule in the prehospital setting
Source: BMJ Open. 2024 Jun 11;14(6):e077191. doi: 10.1136/bmjopen-2023-077191 (PMC11168128; doi:10.1136/bmjopen-2023-077191)
Supplement: Author's manuscript [file bmjopen-2023-077191.draft_revisions.pdf]

BMJ Open

BMJ Open is committed to open peer review. As part of this commitment we make the peer review history of every article we publish publicly available.

When an article is published we post the peer reviewers' comments and the authors' responses online. We also post the versions of the paper that were used during peer review. These are the versions that the peer review comments apply to.

The versions of the paper that follow are the versions that were submitted during the peer review process. They are not the versions of record or the final published versions. They should not be cited or distributed as the published version of this manuscript.

BMJ Open is an open access journal and the full, final, typeset and author-corrected version of record of the manuscript is available on our site with no access controls, subscription charges or pay-per-view fees (http://bmjopen.bmj.com).

If you have any questions on BMJ Open's open peer review process please email info.bmjopen@bmj.com

BMJ Open

Head Injury Evaluation and Ambulance Diagnosis (HOME) Study: A Feasibility Study Assessing the Implementation of the Canadian CT Head Rule in the Prehospital Setting

Journal:	*BMJ Open*
Manuscript ID	bmjopen-2023-077191
Article Type:	Protocol
Date Submitted by the Author:	27-Jun-2023
Complete List of Authors:	Alqurashi, Naif; The University of Manchester, Division of Cardiovascular Sciences; King Saud University, Department of Accidents and Trauma, Prince Sultan bin Abdelaziz College for Emergency Medical Services, King Saud University, Riyadh, Saudi Arabia.<br>Bell, Steve; North West Ambulance Service NHS Trust, Medical Directorate<br>Carley, Simon D; Manchester University NHS Foundation Trust, Emergency Department<br>Lecky, Fiona; University of Sheffield, School of Health and Related Research<br>Body, Richard; The University of Manchester, Division of Cardiovascular Sciences
Keywords:	ACCIDENT & EMERGENCY MEDICINE, Feasibility Studies, TRAUMA MANAGEMENT

SCHOLARONE™
Manuscripts

Head Injury Evaluation and Ambulance Diagnosis (HOME) Study: A Feasibility Study Assessing the Implementation of the Canadian CT Head Rule in the Prehospital Setting

Naif Alqurashi (1,2) Steve Bell (3), Simon D Carley (4), Fiona Lecky (5), Richard Body (1,4)

1. Division of Cardiovascular Sciences, University of Manchester, Manchester, UK.
2. Department of Accidents and Trauma, Prince Sultan bin Abdulaziz College for Emergency Medical Services, King Saud University, Riyadh, Saudi Arabia.
3. Medical Directorate, North West Ambulance Service NHS Trust, Bolton, BL1 5DD, UK.
4. Emergency Department, Manchester University NHS Foundation Trust, Manchester, UK.
5. University of Sheffield, School of Health and Related Research, Sheffield, UK.

***Corresponding author:** Naif Alqurashi, Division of Cardiovascular Sciences, University of Manchester, 46 Grafton Street, Manchester, M13 9PL, United Kingdom; E-mail: nalqurashi1@ksu.edu.sa

Key words: Prehospital; Traumatic brain injury; Triage; Trauma centre; Feasibility study

Word count: 3988 words, excluding the abstract, strengths and limitations, tables and the declarations.

For peer review only

Abstract

Introduction

Traumatic brain injury (TBI) is a common presentation in the prehospital environment. At present, paramedics do not routinely use tools to identify low-risk patients who could be left at scene or taken to a local hospital rather than a major trauma centre. The Canadian Computed Tomography Head Rule (CCHR) was developed to guide the use of computed tomography imaging in hospital. It has not been evaluated in the prehospital setting. We aim to address this gap by evaluating the feasibility and acceptability of implementing the CCHR to patients and paramedics, and the feasibility of conducting a full-scale clinical trial of its use.

Methods and analysis

We will recruit adult patients who are being transported to an emergency department (ED) by ambulance after suffering a mild TBI. Paramedics will prospectively collect data for the CCHR. All patients will be transported to the ED, where deferred consent will be taken and the treating clinician will re-assess the CCHR, blinded to paramedic interpretation. The primary clinical outcome will be neurosurgically significant TBI. Feasibility outcomes include recruitment and attrition rates. We will assess acceptability of the CCHR to paramedics using the Ottawa Acceptability of Decision Rules Instrument. Inter-observer reliability of the CCHR will be assessed between paramedics and the treating clinician in the ED. Participating paramedics and patients will be invited to participate in semi-structured interviews to explore the acceptability of trial processes and facilitators and barriers to the use of the CCHR in practice. Data will be analysed thematically. We anticipate recruiting approximately 100 patients over six months.

For peer review only

Ethics and dissemination

This study was approved by the Health Research Authority and the Research Ethics Committee (REC reference: 22/NW/0358). The results will be published in a peer-reviewed journal, presented at conferences, and will be incorporated into a doctoral thesis.

For peer review only

Strengths and limitations of this study

- To our knowledge, this study will be the first to evaluate the feasibility and acceptability of implementing the Canadian CT Head Rule (CCHR) in the prehospital setting, filling an important gap in the existing literature.
- The study has a limited sample size, as its primary objective is to assess the acceptability and feasibility of implementing the CCHR in the prehospital setting.
- The findings of this study will be used in the future to inform the design of a fully powered diagnostic test accuracy study.

For peer review only

Introduction

Traumatic brain injury (TBI) is widely recognised as being one of the most common causes of death and disability especially among young adults (1); however, it is becoming more frequent in the older population (2). TBI is a heterogeneous pathophysiological condition that varies widely in terms of its aetiology, severity, clinical presentation and outcomes. This broad spectrum of severity creates clinical challenges, as many patients with mild TBI may be admitted to a major trauma centre (MTC), leading to the inappropriate allocation of MTC resources for minor trauma (3).

The goal of prehospital trauma triage tools is to allocate patients efficiently and promptly to the medical facility that is most appropriate for their medical needs and for the resources available. Most TBIs are mild in nature and can be treated at a local trauma unit or non-specialised acute hospital (4). There is no evidence indicating that these patients should be transported to a specialist neurological centre. Therefore, accurate prehospital triage of these patients will reduce the likelihood of MTCs being overwhelmed with patients suffering from mild TBI or non-TBI injuries as well as reducing the costs that might result from over-triaging patients who are unlikely to benefit from the costly resources of an MTC.

The cranial CT scan is considered the gold standard for the emergency evaluation of patients with suspected intracranial injury (5). Various validated clinical tools have been developed to determine whether a CT scan is needed to rule out intracranial lesions in adult patients with mild TBI. Of these, the Canadian CT Head Rule (CCHR) was developed through a series of prospective studies, which involved thousands of patients, to determine the need for neuroimaging (6, 7). A comparison of the various clinical tools used in different countries and settings, most notably the CCHR and the New Orleans Criteria (NOC), has been conducted.

For peer review only

While there have been some variations in the results of these comparisons, the CCHR is more specific than the NOC, although both have a similar level of sensitivity (8-10). CCHR is a highly sensitive rule to assist clinicians in identifying patients with clinically significant head injuries who should undergo CT scanning (6). Many studies have found that that the CCHR has 100% sensitivity in identifying patients who require neurosurgical interventions, as well as capturing clinically significant findings on a CT scan and is more specific (with a specificity rate 36%-65%) than other clinical decision rules. (9-11).

The CCHR rule consists of five high-risk clinical factors for neurosurgical interventions and two medium-risk factors for predicting brain injury on a CT scan (6). It takes into account a number of variables related to the patient's medical history and the findings of physical examinations. Considering that paramedics have access to all the necessary data for the CCHR, it might be feasible to utilise the CCHR in the prehospital setting to promote consistency in care and optimise resource utilisation. Prehospital implementation of the CCHR could allow paramedics to transport patients to lower-level or non-trauma centres, or even (if shown to be sufficiently sensitive) safely leave patients at the scene. However, no studies have explored whether clinical decision aids can be used to rule out neurosurgically significant TBI in patients with apparently mild TBI in the prehospital field.

To obtain reliable estimates of the diagnostic accuracy of the CCHR, a large, multi-centre study is warranted. This would be expensive and time consuming; however, the findings of such a study could inform changes to clinical practice. Successful delivery of such research would depend heavily upon adherence to the study protocol and the rates of recruitment attrition. Before embarking on such a study, it is necessary to evaluate the viability of the intended study processes (12).

For peer review only

The purpose of this study is to establish the feasibility of a fully powered diagnostic-test accuracy study to evaluate the accuracy of the CCHR when applied by paramedics to patients with mild TBI in the prehospital setting.

Research question

Is it feasible to conduct a prospective clinical study to evaluate the diagnostic accuracy of the CCHR in the prehospital field to assist with making informed decisions regarding the appropriate level of care for patients with minor head injuries?

Objectives

- To determine the feasibility of a study to evaluate the diagnostic accuracy of the CCHR in the prehospital environment.
- To evaluate the inter-observer reliability of the CCHR between prehospital clinicians and ED physicians.
- To evaluate the clinical sensibility of using the CCHR in the prehospital environment and to measure paramedics' attitudes toward the CCHR.
- To obtain an estimate of the diagnostic accuracy of the CCHR when used in the prehospital setting.

Methods

Study design and setting

A prospective, multi-centre feasibility study will be conducted. The study will take place in Greater Manchester, a region covered by the North West Ambulance Service, which is a National Health Service (NHS) trust providing emergency medical services and patient

For peer review only

transportation throughout the North West region of England. This study protocol has been prospectively registered through the ISRCTN registry (study ID: ISRCTN92566288).

For the purpose of the study, we modified the CCHR for prehospital use (Table 1). The original rule excluded patients <16 years, patients with a bleeding disorder or users of oral anticoagulants and/or antiplatelet and or those experiencing a post-traumatic seizure. In the version modified for this study, only patients under the age of 16 years will be excluded. We will include these patients in the study, but those patients who are taking anticoagulants and/or antiplatelet medications will be considered to be 'positive' with the modified CCHR (i.e., would require transport to hospital). Anticoagulant use is defined as the use of warfarin, edoxaban, dabigatran, apixaban, and rivaroxaban. Antiplatelets use is defined as the use of clopidogrel, ticlopidine, ticagrelor, dipyridamole and prasugrel. Figure 1 displays a visual representation of the recruitment process.

Participants

Patient eligibility

Inclusion criteria:

This study will be conducted using a convenience sample of adult patients with mild TBI who will be transported to Northern Care Alliance NHS Foundation Trust and Manchester University Hospital NHS Foundation Trust. Only those patients who meet the following criteria will be included:

- Adult patients (≥16 years) who receive an emergency ambulance response for a primary complaint of head injury.
- Glasgow Coma Scale (GCS) score of 13–15 at the time of assessment by attending paramedics.

For peer review only

- Patients transported to the hospital for clinical care.

Exclusion criteria:

- Secondary transfers patients including interfacility transport.
- Penetrating skull injury.
- Trauma to other body regions that that require clinical treatment.
- Prisoners.

Table1: The original and modified versions of the Canadian CT Head rule

**Canadian CT head rule (original)**	**Canadian CT head rule (modified)**
**High risk (for neurosurgical interventions)**	**High risk (for neurosurgical interventions)**
- GCS score <15 at 2 hrs after injury	- GCS score <15 at presentation
- Suspected open or depressed skull fracture	- Suspected open or depressed skull fracture
- Any sign of basal skull fracture	- Any sign of basal skull fracture
- Vomiting ≥ 2 episodes	- Vomiting ≥ 2 episodes
- Age ≥ 65 years	- Age ≥ 65 years
**Medium risk (for brain injury on CT)**	**Medium risk (for brain injury on CT)**
Amnesia before impact > 30 minutes	- Amnesia before impact > 30 minutes
Dangerous mechanism of injury (pedestrian struck by vehicle, ejection from vehicle, fall from >3 feet or >5 stairs.	Dangerous mechanism of injury (pedestrian struck by vehicle, ejection from vehicle, fall from >3 feet or >5 stairs.
* Patients with any positive high risk or medium risk criteria will be considered to have a 'positive' outcome (requiring transport to a hospital)	

For peer review only

Study duration and sample size

This is a feasibility study, in which evaluating recruitment rate is an outcome; therefore, it is not possible to specify a sample size a priori. However, we intend to recruit participants for a fixed period of six months. Based on data routinely collected by the Manchester University NHS Foundation Trust, a total of 5,332 patients arrived in the ED by ambulance with a primary complaint of head injury over a period of four years and nine months up to November 2020. We intend to recruit participants from two similarly sized MTCs. We anticipate that approximately 1,100 patients will be eligible to participate in this study during the recruitment period. Accounting for the availability of trained paramedics on the study delegation log and concurrent injuries to other body systems that would exclude patients from participating in this study, approximately 100 participants can be recruited to this study.

Outcomes

We will study the following feasibility outcomes:

- The number of eligible patients who are approached.
- The proportion of those approached who consent to participate in the study.
- The completeness of data collection.
- The completeness of follow-up.
- Determination of the inter-observer reliability of the CCHR completed by prehospital care providers and ED physicians.
- Determination of the inter-observer reliability for each component of the CCHR assessed by prehospital care providers and ED physicians.
- The diagnostic performance and clinical sensibility of the CCHR in the opinion of attending paramedics.

For peer review only

- Determine the acceptability of trial processes as perceived by paramedics and patients.

Additionally, as a preliminary evaluation of the diagnostic accuracy of the CCHR, the presence of traumatic intracranial lesions requiring neurosurgical intervention will be examined. This includes any neurosurgical intervention and/or abnormal head CT findings and/or death within seven days resulting from TBI.

Measuring and defining outcomes

Feasibility outcomes will be assessed using screening logs and case report forms completed by attending paramedics during the course of the study. At the end of the study period, a minimum of two clinicians (affiliated with the relevant NHS trusts) will review in-hospital patient records to diagnose the secondary outcomes of this feasibility study (neurosurgical significant TBI, abnormal CT findings and death within seven days). A third independent investigator will be invited to adjudicate any discrepancies. Diagnostic adjudication will be blinded to the final interpterion of the CCHR. Additionally, a neuroradiology consultant/expert will be consulted in case of the CT scan diagnosis is inconclusive.

Neurosurgical interventions are defined as any neurosurgical intervention including intracranial pressure monitoring or an Abbreviated Injury Scale (AIS) with the severity ≥3 in the head region. Abnormal head CT findings will be classified as normal or abnormal and are defined as at least one of the following: skull fractures, intracranial lesions, such as cerebral contusion and traumatic intracranial haemorrhages, or other clinically significant findings requiring hospital admission, neurosurgical interventions and routine follow-up.

For peer review only

Data collection

Prehospital setting

During the study period, all patients will receive standard prehospital care in line with local guidelines. All participating paramedics will be provided with bespoke study training prior to taking part in this study. Training on all aspects of the study protocol will be provided, including eligibility assessment, consent process, data collection procedures and guidance on interpreting the CCHR.

Prehospital data will be collected using Research Electronic Data Capture (REDCap), a secure web-based platform hosted at the University of Manchester. REDCap is a software application that was created to facilitate the process of collecting and managing research data. Prior to recruitment, a training session will be held to equip participating sites with data collection skills. In addition, paper case report forms (CRFs) will be available as an alternative method of data collection. It is expected that this approach will provide flexibility and ensure continuity in data collection. A variety of data will be collected in the prehospital setting, including, but not limited to:

- Baseline demographics information (i.e, gender - age)
- Anticoagulant use.
- Injury characteristics (i.e., mechanism of injury, place).
- Past medical history.
- Initial chief complaint (i.e., vomiting, headache, loss of consciousness, amnesia).
- Prehospital physiological parameters (i.e., GCS, vital signs).
- Clinical criteria required to use the CCHR.
- Destination hospital.

For peer review only

In-hospital environment

REDCap will be utilised to collect hospital data, and a paper CRF will also be available as an alternative. Emergency physicians at the receiving hospital will assess the patients to determine whether a CT scan is necessary in accordance with local and national guidelines. Relevant data about imaging studies undertaken and their findings will be extracted from hospital records. The routine radiology reports will be used to extract the follow-up data required for this study. Where there is uncertainty about the interpretation of the radiology report, a second radiologist will be asked to interpret the images.

Further, emergency physicians at the receiving hospitals will be asked to assess the patients using the CCHR and record their findings, blinded to the assessments made by participating paramedics. These data will be used to determine the inter-observer reliability of the CCHR between paramedics in the prehospital environment and clinicians in the ED. Additionally, in-hospital medical records will be reviewed to obtain information regarding neurosurgical interventions undertaken during the patient's hospital admission.

Follow-up

Following the initial presentation to the ED, patients will be followed up to the time of hospital discharge. Data will be collected from the clinical records of the patient, including, but not limited to

- GCS on arrival in hospital.
- Details of the clinician's assessment of the CCHR.
- Outcome of any brain imaging (i.e., CT and/or MR scans, recorded as part of routine practice).
- AIS in relation to head injury.

For peer review only

- Injury Severity Score.
- Mortality status.
- Neurosurgical procedures and/or surgery.

Evaluation of acceptability

We will use a qualitative approach to evaluate the acceptability of trial processes used to study participants. We will purposively sample (a) study participants and (b) paramedics. The intention is to maximise heterogeneity (according to age, sex and ethnic origin, and for paramedics, seniority and base ambulance station). Patients and participating paramedics will be interviewed for the purpose of exploring their experiences of participating in a study of this nature. Previous emergency care research has demonstrated the value of this method in providing information regarding the feasibility and design of a larger study (13).

To maximise convenience for participants and thereby recruitment, participants will be invited to take part in a semi-structured interview either by telephone, face-to-face or video conference. Informed consent will be obtained from all participants. We will explore the factors that facilitate or discourage participation in the research. Paramedics and study participants will be offered a £30 high street voucher to thank them for taking part. Telephone and sign language interpreters will be provided where necessary. The interview topic guides (Appendix 1) consist of a number of questions and prompts concerning the patients and paramedics' experiences of participating in this study, as well as the paramedics’ perceptions of the viability of the CCHR. Prior to commencement of the interviews, two pilot interviews will be carried out. To determine the relevance, clarity and duration required to complete each interview, one will be with a paramedic and the other will be with a patient (14).

For peer review only

The Ottawa Acceptability of Decision Rules Instrument (OADRI) is a 12-item instrument that will be used to measure paramedics' attitudes toward the CCHR and clinical sensibility (15). Paramedics will be asked to provide their level of agreement with twelve statements about the CCHR using a six-point Likert scale. The first seven statements are worded in such a way that a higher number indicates higher acceptance, while the last five are worded in the opposite manner to minimise the acquiescence bias (16).

Ethical approval and consent process

Ethical approval to conduct this feasibility study has been obtained from the Health Research Authority and the Research Ethics Committee (REC reference: 22/NW/0358). To ensure privacy and confidentiality, all records and files relating to the study will be kept in a secure location and will only be accessible to members of the research team.

An exception to the normal process of informed consent is established for patients in emergency situations who are unable to provide consent. As stated in the Declaration of Helsinki, "for a research subject who is legally incompetent, physically or mentally incapable of giving consent or is a legally incompetent minor, the investigator must obtain informed consent from the legally authorised representative in accordance with applicable law. These groups should not be included in research unless the research is necessary to promote the health of the population represented and this research cannot instead be performed on legally competent persons” (17).

In this study, we will include patients in the prehospital environment who are receiving an emergency ambulance response having sustained a head injury. The nature of this presentation means that mental capacity to provide informed consent for participation in research will be

For peer review only

compromised, especially given the time critical nature of the emergency medical response. Therefore, we will adopt a deferred consent model, which is widely used in research of this nature (18).

Paramedics will enrol eligible participants, enabling them to collect the required data. Patient care will be unchanged. We will then seek deferred written informed consent after the patients have received their initial treatment in hospital, and once they have regained their mental capacity.

When seeking written informed consent, a participant information sheet will be provided. Deferred consent may be obtained by either of the following means: (a) a member of staff may seek written informed consent at the receiving hospital; or (b) deferred consent may be sought in the community by post (written informed consent), email or telephone. While seeking deferred consent, staff will be available to answer any questions that the participants may have.

For participants lacking capacity, we will seek consent from their relative or legally authorised representative, which is known as proxy consent (19). A research nurse will briefly explain to them that the patient will receive the usual hospital care for TBI. The responsible nurse will also clarify that only basic clinical data will be reported using a case report form to be analysed to improve prehospital care. A brief information sheet will be provided upon request. The ability of participants to provide consent can be assessed at any point during the study period. Participants who regain capacity will be provided with a detailed participant information sheet and their written consent to continue in this feasibility study will be obtained. If the patient does not regain capacity, we will seek deferred assent from a relative or professional legal representative to retain the patient's data in the study.

For peer review only

Data analysis

Quantitative data

A descriptive analysis will be conducted based on the baseline data for patients enrolled in the study. It will include an estimation of the means and standard deviations, as well as the proportion of patients who are eligible and willing to participate in this feasibility study. The inter-observer reliability between paramedics and emergency physicians' use of the CCHR will be calculated using Cohen's Kappa, a statistical method for measuring agreement between two observers, together with 95% confidence intervals. Additionally, we will report upon the CCHR's diagnostic performance for ruling out clinically significant patients with mild TBI. This will be achieved by calculating the sensitivity, specificity, positive and negative likelihood ratios with 95% confidence intervals against a reference standard of traumatic intracranial lesion, which will be identified from in-hospital records. Given the nature of this feasibility study, the analyses will be viewed as exploratory, preliminary and will be descriptive in nature. Furthermore, to determine the acceptability of the CCHR, the OADRI average item scores will be calculated, with higher scores indicating greater acceptability.

Qualitative data

The six-stage thematic analysis approach will be employed to analyse all interviews, as suggested by Braun and Clarke (20). This involves becoming familiar with the data, generating initial codes, searching for themes, reviewing themes, defining and naming themes and sub-themes, and producing the report. We will adhere to the four criteria for trustworthiness established by Lincoln and Guba: credibility, dependability, confirmability and transferability (21). Each step of the qualitative data collection and analysis will be guided by a qualitative research expert. The qualitative data will be managed and analysed using NVivo 12.

For peer review only

Progression criteria

This feasibility study will be overseen and governed by the Trial Steering Committee (TSC), which will include the chief investigator, principal investigator, two lay members, topic experts in TBI and one paramedic. The role of the TSC is to provide external oversight and to ensure that the study is conducted in compliance with Good Clinical Practice guidelines (22, 23).

The traffic light approach (green/amber/red), also known as a (stop-amend-go) rule, will be used to determine whether or not to proceed with a full definitive trial (24). Green (go) indicates that the study meets the pre-set criteria, and that it would be feasible for a full-powered definitive trial to be conducted without any modifications to the study protocol. Amber (amend) indicates that further feasibility work or modifications to the protocol are required before proceeding with the full trial. Red (stop) indicates that proceeding to a definitive study is not recommended. Taking into account the feasibility outcomes, as well as qualitative interviews with patients and paramedics, a decision will be made regarding whether or not to proceed with the trial or whether to make any amendments to the study design. The progression criteria are shown in Table 2.

Patient and public involvement

It has become increasingly apparent that involving patients and the public in healthcare research can enhance the development and implementation of clinical studies, the recruitment of participants, and the relevance of study topics and outcomes in relation to their needs and experiences (25, 26). It will be a priority to take into account the perspectives of patients and the public during the design, conduct, reporting and dissemination of the research. To ensure maximum clinical impact, the study has been discussed with patient and public representatives through the NIHR Brain Injury MedTech Co-operative. A comprehensive and in-depth

For peer review only

discussion was conducted regarding the study procedures, including key aspects such as the consent process and patient-facing materials. Two members have agreed to join the study steering committee, which is expected to meet two or three times during the study period to advise on the progress of the research, discuss any adverse events, proposed protocol amendments and other matters relating to research governance. Additionally, a lay summary will be developed in collaboration with the lay members to disseminate the study findings.

Table 2: Progression criteria to proceed with a definitive trial

	Contributing data	Green (Proceed to a definitive trial)	Amber (Proceed to a definitive trial following some changes to the protocol	Do not proceed to a definitive trial
**Screening**	Training and delegation logs	At least 20 paramedics at participating ambulance hubs trained and on delegation logs	At least 10 paramedics at participating ambulance hubs trained and on delegation logs	Less than 10 paramedics at participating ambulance hubs trained and on delegation logs
**Recruitment acceptability**	Screening & recruitment log	≥ 80% of eligible screened patients are consented to take part	≥ 60% of eligible screened patients are consented to take part	< 60% of eligible screened patients are consented to take part
**Retention**	Screening & recruitment log	Retention of ≥ 90% of study participants until study completion	Retention of ≥ 80% of study participants until study completion	Retention of < 80% of study participants until study completion
**Study procedures**	Participation data	At least 85% of the outcome data is collected (e.g., case report forms)	A minimum of 60% of the outcome data is collected (e.g., case report forms)	< 60% of the outcome data is collected (e.g., case report forms)
**Adverse events**	Participation data	No or very minor adverse events and no participants discontinued the study	Minor or serious adverse events leading to 10% or less participants discontinuing the study	Serious adverse events leading to >10% of participants discontinuing the study
**Acceptability**	Participant interview Paramedics questionnaire	Responses to OADRI* all 'agree' or 'strongly agree'. No major barriers to acceptability/participation identified during	Responses to OADRI mainly 'agree' or 'strongly agree'. Some barriers to participation identified during interviews	Responses to OADRI mainly 'disagree' or 'strongly disagree'. Major barriers to acceptability/participation

	and interviews	interviews with paramedics and patients	but remediable action identified.	identified during interviews with no clear remediable action.

* OADRI: Ottawa Acceptability of Decision Rules Instrument

Safety considerations, adverse events and serious adverse events

Given the nature of this study, any risk to participants is very low. It is important to note that this study is observational in nature: a patient's participation in this study will have no effect on the quality of medical care they usually receive in both prehospital and hospital settings. Our study will have no impact on the decision to undergo a CT scan as this will be undertaken at the discretion of the treating clinician, as part of routine clinical care, in accordance with current national guidance. The study will also have no effect on the decision to transport patients to hospital. There is also a potential risk of data-sharing to be considered. There are small risks associated with data management, but we have carefully considered how data will be handled during the study and we will operate in accordance with current legislation and the standard operating procedures of the sponsor organisation to mitigate that risk. We are not aware of any potential risks this study would pose to the researchers.

Because of the low-risk nature of the study, we do not anticipate any adverse events (including serious adverse events) as a result of participating in this research. However, given the patient group (patients with TBI) we anticipate that many patients will experience expected adverse events, which are related to their initial injury. We will not ask sites to report expected adverse events. Expected serious adverse events include, for example, death due to head injury; neurosurgical procedures for TBI; admission to hospital for traumatic brain injury; loss of function due to TBI. Unexpected adverse events and serious adverse events should be reported.

For peer review only

Impact

It is anticipated that the feasibility study will provide valuable insight into whether conducting a full trial in the future is warranted. It will also provide guidance to enhance the study design, recruitment plan, and consent process. If feasibility is established, we will prepare to run a fully powered multi-centre study to evaluate the diagnostic accuracy of using the CCHR in the prehospital field. This will also involve drafting a proposal to apply for research funding.

The outcomes relating to diagnostic accuracy will be of particular relevance given our knowledge that this is the first study investigating the feasibility of using the CCHR in the prehospital environment. Therefore, it is anticipated that the findings of this feasibility study will be used to inform design of a fully powered diagnostic test accuracy study. The findings of this study will be complemented by the findings of mixed methods research that we are also conducting which will explore facilitators and barriers to implementing new diagnostic pathways in the prehospital environment as perceived by practising paramedics (27).

Dissemination

The results will be published in peer-reviewed journals, presented at relevant national and international conferences, and will be incorporated into a doctoral thesis (NA). The study findings will also be disseminated through presentations at relevant academic forums.

For peer review only

Declarations

Ethics approval and consent to participate

The nature of this study requires ethical approval from the Health Research Authority and Research Ethics Committee, which has been obtained (REC reference: 22/NW/0358).

Consent for publication

Not applicable.

Availability of data and material

Not applicable.

Competing interests

Richard Body has consulted for Abbott Point of Care and is undertaking contract clinical research with BrainBox, which manufactures assays for biomarkers of TBI.

Funding

This work is supported by a PhD scholarship awarded to NA by the Saudi Arabian Cultural Bureau in the UK and King Saud University (KSU1872). The funders had no role in the design of this study protocol. The publication fee is funded by the cardiovascular department, University of Manchester, UK. No grant or award number.

Authors contributions

NA collaborated with the study team (SB, SC, FL, and RB) in preparing the initial study protocol. The protocol was developed through collective input and discussions. NA took the lead in drafting the manuscript, working closely with SB, SC, FL, and RB to ensure its accuracy and completeness. The final version of the manuscript was approved by all authors.

For peer review only

Acknowledgements

Not applicable.

Author affiliations

[1]Division of Cardiovascular Sciences, University of Manchester, Manchester, UK. [2]Department of Accidents and Trauma, Prince Sultan bin Abdulaziz College for Emergency Medical Services, King Saud University, Riyadh, Saudi Arabia. [3]Medical Directorate, North West Ambulance Service NHS Trust, Bolton, BL1 5DD, UK. [4] Emergency Department, Manchester University NHS Foundation Trust, Manchester, UK. [5]University of Sheffield, School of Health and Related Research, Sheffield, UK.

For peer review only

References

1. Berkner J, Mannix R, Qiu J. Clinical Traumatic Brain Injury in the Preclinical Setting. Methods Mol Biol. 2016;1462:11-28.
2. Majdan M, Plancikova D, Brazinova A, Rusnak M, Nieboer D, Feigin V, et al. Epidemiology of traumatic brain injuries in Europe: a cross-sectional analysis. Lancet Public Health. 2016;1(2):e76-e83.
3. Anandalwar SP, Mau CY, Gordhan CG, Majmundar N, Meleis A, Prestigiacomo CJ, et al. Eliminating unnecessary routine head CT scanning in neurologically intact mild traumatic brain injury patients: implementation and evaluation of a new protocol. J Neurosurg. 2016;125(3):667-73.
4. Decuypere M, Klimo P, Jr. Spectrum of traumatic brain injury from mild to severe. Surg Clin North Am. 2012;92(4):939-57, ix.
5. Haydel MJ, Preston CA, Mills TJ, Luber S, Blaudeau E, DeBlieux PM. Indications for computed tomography in patients with minor head injury. N Engl J Med. 2000;343(2):100-5.
6. Stiell IG, Lesiuk H, Wells GA, McKnight RD, Brison R, Clement C, et al. The Canadian CT Head Rule Study for patients with minor head injury: rationale, objectives, and methodology for phase I (derivation). Ann Emerg Med. 2001;38(2):160-9.
7. Stiell IG, Lesiuk H, Wells GA, Coyle D, McKnight RD, Brison R, et al. Canadian CT head rule study for patients with minor head injury: methodology for phase II (validation and economic analysis). Ann Emerg Med. 2001;38(3):317-22.
8. Stiell IG, Clement CM, Rowe BH, Schull MJ, Brison R, Cass D, et al. Comparison of the Canadian CT Head Rule and the New Orleans Criteria in patients with minor head injury. Jama. 2005;294(12):1511-8.
9. Papa L, Stiell IG, Clement CM, Pawlowicz A, Wolfram A, Braga C, et al. Performance of the Canadian CT Head Rule and the New Orleans Criteria for predicting any traumatic intracranial injury on computed tomography in a United States Level I trauma center. Acad Emerg Med. 2012;19(1):2-10.
10. Bouida W, Marghli S, Souissi S, Ksibi H, Methammem M, Haguiga H, et al. Prediction value of the Canadian CT head rule and the New Orleans criteria for positive head CT scan and acute neurosurgical procedures in minor head trauma: a multicenter external validation study. Ann Emerg Med. 2013;61(5):521-7.
11. Bonney PA, Briggs A, Briggs RG, Jarvis CA, Attenello F, Giannotta SL. Rate of Intracranial Hemorrhage After Minor Head Injury. Cureus. 2020;12(9):e10653.
12. Arain M, Campbell MJ, Cooper CL, Lancaster GA. What is a pilot or feasibility study? A review of current practice and editorial policy. BMC medical research methodology. 2010;10(1):1-7.
13. van den Berg P, Kendal S, Alderson HV, Body R. An exploration of patients' experiences of participation in a randomised controlled trial of the Manchester Acute Coronary Syndromes (MACS) decision rule. Emerg Med J. 2017;34(9):593-8.
14. Majid MAA, Othman M, Mohamad SF, Lim SAH, Yusof A. Piloting for interviews in qualitative research: Operationalization and lessons learnt. International Journal of Academic Research in Business and Social Sciences. 2017;7(4):1073-80.
15. Brehaut JC, Graham ID, Wood TJ, Taljaard M, Eagles D, Lott A, et al. Measuring acceptability of clinical decision rules: validation of the Ottawa acceptability of decision rules instrument (OADRI) in four countries. Medical Decision Making. 2010;30(3):398-408.

For peer review only

16. Schriesheim C, Hill K. Controlling Acquiescence Response Bias by Item Reversals: The Effect on Questionnaire Validity. Educational and Psychological Measurement - EDUC PSYCHOL MEAS. 1981;41:1101-14.
17. Association WM. World Medical Association Declaration of Helsinki: ethical principles for medical research involving human subjects. Jama. 2013;310(20):2191-4.
18. Kompanje E, Jansen T, Le Noble J, de Geus H, Bakker J. Deferred consent for inclusion of patients unable to give their consent in studies in the field of emergency medicine. Nederlands Tijdschrift Voor Geneeskunde. 2008;152(38):2057-61.
19. Kompanje EJO, Maas AIR, Hilhorst M, Slieker F, Teasdale G. Ethical considerations on consent procedures for emergency research in severe and moderate traumatic brain injury. Acta Neurochirurgica. 2005;147:633-40.
20. Braun V, Clarke V. Using thematic analysis in psychology. Qualitative research in psychology. 2006;3(2):77-101.
21. Forero R, Nahidi S, De Costa J, Mohsin M, Fitzgerald G, Gibson N, et al. Application of four-dimension criteria to assess rigour of qualitative research in emergency medicine. BMC Health Serv Res. 2018;18(1):120.
22. Daykin A, Selman L, Cramer H, McCann S, Shorter G, Sydes M, et al. What are the roles and valued attributes of a Trial Steering Committee? Ethnographic study of eight clinical trials facing challenges. Trials. 2016;17:307.
23. Harman NL, Conroy EJ, Lewis SC, Murray G, Norrie J, Sydes MR, et al. Exploring the role and function of trial steering committees: results of an expert panel meeting. Trials. 2015;16:597.
24. Avery KNL, Williamson PR, Gamble C, Connell Francischetto E, Metcalfe C, Davidson P, et al. Informing efficient randomised controlled trials: exploration of challenges in developing progression criteria for internal pilot studies. BMJ Open. 2017;7(2):e013537.
25. Price A, Albarqouni L, Kirkpatrick J, Clarke M, Liew SM, Roberts N, et al. Patient and public involvement in the design of clinical trials: an overview of systematic reviews. Journal of evaluation in clinical practice. 2018;24(1):240-53.
26. Gamble C, Dudley L, Allam A, Bell P, Goodare H, Hanley B, et al. Patient and public involvement in the early stages of clinical trial development: A systematic cohort investigation. BMJ open. 2014;4:e005234.
27. Alqurashi N, Alotaibi A, Bell S, Lecky F, Body R. Towards exploring current challenges and future opportunities relating to the prehospital triage of patients with traumatic brain injury: a mixed-methods study protocol. BMJ Open. 2023;13(3):e068555.

For peer review only

Figure legends:

- **Figure 1: Study flowchart**

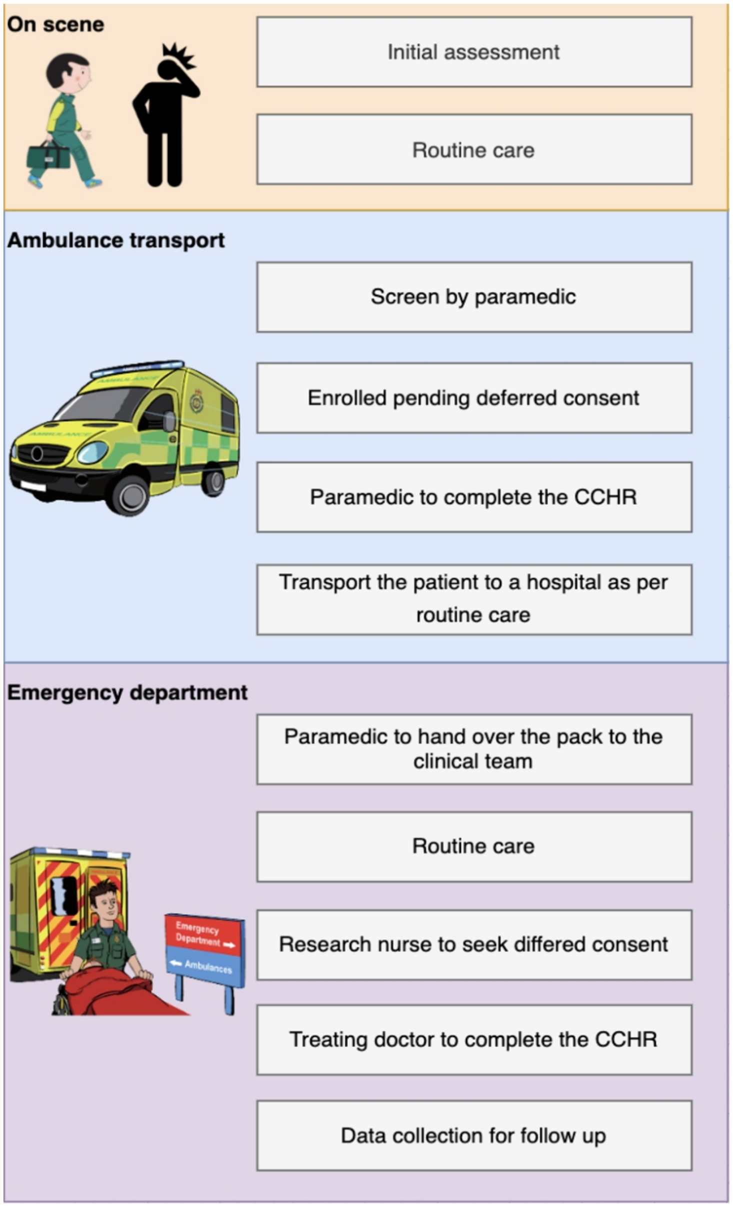

Figure 1: Study flowchart

149x247mm (144 x 144 DPI)

Head injury Evaluation and Ambulance Diagnosis: A Feasibility Study

Semi-structured interview, topic guide – Patients

Date: **Participant ID number:**

Pre-interview

- Privacy notice read.
- Consent form.
- Information sheet.

---

1. Please share your experience from the moment you were initially approached about the study.
   - **Follow-up questions, if required:**
   a. How did you feel about being approached for the study? Were there any specific factors that influenced your decision to participate?
   b. Were you given enough information to help you to decide whether to take part in this study? Was there anything that you found confusing or unclear?
   c. In this study, we didn't ask patients for permission during the ambulance journey. This is because they had experienced a head injury and were being taken to hospital as an emergency. How did you feel about this? Do you think you would have been able to make a decision about taking part when you were still in the ambulance?

2. How did you feel about the way we asked for your permission to take part in this study?
   - **Follow-up questions, if required:**
   a. Please share your overall thoughts about how we asked for your permission to take part in this study.
   b. Is there anything particularly good or bad about the way we asked for your permission to take part in the study?
   c. Do you think you were given enough time to decide whether to take part? Why or why not?

3. Can you recall any specific reasons or factors that influenced your belief that it is important to understand the study? How did this understanding affect your decision to take part in this study?

For peer review only - http://bmjopen.bmj.com/site/about/guidelines.xhtml

4. In your opinion, are there any factors that you believe might discourage people from participating in this study?

Follow-up questions, if required:

   a. Is there anything that might discourage people from participating in this study? How do you think these factors could be addressed?

5. Can you share any unexpected events or experiences that occurred during your participation in the study?

6. Would you recommend taking part in this trial to somebody else?

Follow-up questions, if required:

   a. Can you explain your thoughts and the reasons behind your recommendation or non-recommendation of participation in this study to others?
   b. In your opinion, what specific factors or aspects of the study influenced your recommendation or non-recommendation?
   c. Is there anything that you think would stop people from fully participating in this study?

7. Is there anything else you would still like to know about this study?

******END******

For peer review only - http://bmjopen.bmj.com/site/about/guidelines.xhtml

Head injury Evaluation and Ambulance Diagnosis: A Feasibility Study
Semi-structured interview, topic guide - Paramedics

Date: **Participant ID number:**

Pre-interview

- Privacy notice read.
- Consent form.
- Information sheet.

---

1. Firstly, I would like to know how long you have been a paramedic, and what is your highest academic qualification level?

2. Have you completed the training on the study protocol?
   a. How did you find it?
   b. Did you find the study protocol easy to understand?
   c. Was the protocol easy to remember in practice?
   d. What could be improved in the trial protocol?
3. What was the best aspect of being involved in the study?
   a. Could you tell me how easy it was to use the study case report form?

4. What would be the advantages of using the Canadian CT head Rule in the prehospital setting?
   a. Looking back to your experience, could you tell me how did you find using the Canadian CT head rule in the prehospital field?

5. What would be the disadvantages of using the Canadian CT head Rule in the prehospital setting?
   a. Looking back to your experience, did you experience any barriers/challenges to applying the Canadian CT Head Rule?

6. Would you like the Canadian CT Head Rule to become part of your practice?

7. Do you have any additional thoughts you would like to share about the study?

END

For peer review only - http://bmjopen.bmj.com/site/about/guidelines.xhtml

BMJ Open

Head Injury Evaluation and Ambulance Diagnosis (HOME) Study Protocol: A Feasibility Study Assessing the Implementation of the Canadian CT Head Rule in the Prehospital Setting

Journal:	*BMJ Open*
Manuscript ID	bmjopen-2023-077191.R1
Article Type:	Protocol
Date Submitted by the Author:	15-Dec-2023
Complete List of Authors:	Alqurashi, Naif; The University of Manchester, Division of Cardiovascular Sciences; King Saud University, Department of Accidents and Trauma, Prince Sultan bin Abdelaziz College for Emergency Medical Services, King Saud University, Riyadh, Saudi Arabia.<br>Bell, Steve; North West Ambulance Service NHS Trust, Medical Directorate<br>Carley, Simon D; Manchester University NHS Foundation Trust, Emergency Department<br>Lecky, Fiona; University of Sheffield, School of Health and Related Research<br>Body, Richard; The University of Manchester, Division of Cardiovascular Sciences
**Primary Subject Heading**:	Emergency medicine
Secondary Subject Heading:	Evidence based practice
Keywords:	ACCIDENT & EMERGENCY MEDICINE, Feasibility Studies, TRAUMA MANAGEMENT

SCHOLARONE™
Manuscripts

Head Injury Evaluation and Ambulance Diagnosis (HOME) Study Protocol: A Feasibility Study Assessing the Implementation of the Canadian CT Head Rule in the Prehospital Setting

Naif Alqurashi (1,2) Steve Bell (3), Simon D Carley (4), Fiona Lecky (5), Richard Body (1,4)

1. Division of Cardiovascular Sciences, University of Manchester, Manchester, UK.
2. Department of Accidents and Trauma, Prince Sultan bin Abdulaziz College for Emergency Medical Services, King Saud University, Riyadh, Saudi Arabia.
3. Medical Directorate, North West Ambulance Service NHS Trust, Bolton, BL1 5DD, UK.
4. Emergency Department, Manchester University NHS Foundation Trust, Manchester, UK.
5. University of Sheffield, School of Health and Related Research, Sheffield, UK.

***Corresponding author:** Naif Alqurashi, Division of Cardiovascular Sciences, University of Manchester, 46 Grafton Street, Manchester, M13 9PL, United Kingdom; E-mail: nalqurashi1@ksu.edu.sa

Key words: Prehospital; Traumatic brain injury; Triage; Trauma centre; Feasibility study

Word count: 4263 words, excluding the abstract, strengths and limitations, tables and the declarations.

For peer review only

Abstract

Introduction

Traumatic brain injury (TBI) is a common presentation in the prehospital environment. At present, paramedics do not routinely use tools to identify low-risk patients who could be left at scene or taken to a local hospital rather than a major trauma centre. The Canadian Computed Tomography Head Rule (CCHR) was developed to guide the use of computed tomography imaging in hospital. It has not been evaluated in the prehospital setting. We aim to address this gap by evaluating the feasibility and acceptability of implementing the CCHR to patients and paramedics, and the feasibility of conducting a full-scale clinical trial of its use.

Methods and analysis

We will recruit adult patients who are being transported to an emergency department (ED) by ambulance after suffering a mild TBI. Paramedics will prospectively collect data for the CCHR. All patients will be transported to the ED, where deferred consent will be taken and the treating clinician will re-assess the CCHR, blinded to paramedic interpretation. The primary clinical outcome will be neurosurgically significant TBI. Feasibility outcomes include recruitment and attrition rates. We will assess acceptability of the CCHR to paramedics using the Ottawa Acceptability of Decision Rules Instrument. Inter-observer reliability of the CCHR will be assessed between paramedics and the treating clinician in the ED. Participating paramedics and patients will be invited to participate in semi-structured interviews to explore the acceptability of trial processes and facilitators and barriers to the use of the CCHR in practice. Data will be analysed thematically. We anticipate recruiting approximately 100 patients over six months.

For peer review only

Ethics and dissemination

This study was approved by the Health Research Authority and the Research Ethics Committee (REC reference: 22/NW/0358). The results will be published in a peer-reviewed journal, presented at conferences, and will be incorporated into a doctoral thesis.

For peer review only

Strengths and limitations of this study

- To our knowledge, this study will be the first to evaluate the feasibility and acceptability of implementing the Canadian CT Head Rule (CCHR) in the prehospital setting, filling an important gap in the existing literature.
- The study has a limited sample size, as its primary objective is to assess the acceptability and feasibility of implementing the CCHR in the prehospital setting.
- The findings of this study will be used in the future to inform the design of a fully powered diagnostic test accuracy study.

For peer review only

Introduction

Traumatic brain injury (TBI) is widely recognised as being one of the most common causes of death and disability especially among young adults (1); however, it is becoming more frequent in the older population (2). TBI is a heterogeneous pathophysiological condition that varies widely in terms of its aetiology, severity, clinical presentation and outcomes. This broad spectrum of severity creates clinical challenges, as many patients with mild TBI may be admitted to a major trauma centre (MTC), leading to the inappropriate allocation of MTC resources for minor trauma (3).

The goal of prehospital trauma triage tools is to allocate patients efficiently and promptly to the medical facility that is most appropriate for their medical needs and for the resources available. Most TBIs are mild in nature and can be treated at a local trauma unit or non-trauma centres (4). There is no evidence indicating that these patients should be transported to a specialist neurological centre. Therefore, accurate prehospital triage of these patients will reduce the likelihood of MTCs being overwhelmed with patients suffering from mild TBI or non-TBI injuries as well as reducing the costs that might result from over-triaging patients who are unlikely to benefit from the costly resources of an MTC.

The cranial CT scan is considered the gold standard for the emergency evaluation of patients with suspected intracranial injury (5). Various validated clinical tools have been developed to determine whether a CT scan is needed to rule out intracranial lesions in adult patients with mild TBI. Of these, the Canadian CT Head Rule (CCHR) was developed through a series of prospective studies, which involved thousands of patients, to determine the need for neuroimaging (6, 7). A comparison of the various clinical tools used in different countries and settings, most notably the CCHR and the New Orleans Criteria (NOC), has been conducted.

For peer review only

While there have been some variations in the results of these comparisons, the CCHR is more specific than the NOC, although both have a similar level of sensitivity (8-10). CCHR is a highly sensitive rule to assist clinicians in identifying patients with clinically significant head injuries who should undergo CT scanning (6). Many studies have found that that the CCHR has 100% sensitivity in identifying patients who require neurosurgical interventions, as well as capturing clinically significant findings on a CT scan and is more specific (with a specificity rate 36%-65%) than other clinical decision rules. (9-11).

The CCHR rule consists of five high-risk clinical factors for neurosurgical interventions and two medium-risk factors for predicting brain injury on a CT scan (6). It takes into account a number of variables related to the patient's medical history and the findings of physical examinations. Considering that paramedics have access to all the necessary data for the CCHR, it might be feasible to utilise the CCHR in the prehospital setting to promote consistency in care and optimise resource utilisation. Prehospital implementation of the CCHR could allow paramedics to transport patients to lower-level or non-trauma centres, or even (if shown to be sufficiently sensitive) safely leave patients at the scene. However, no studies have explored whether clinical decision aids can be used to rule out neurosurgically significant TBI in patients with apparently mild TBI in the prehospital field.

There is a substantial opportunity to improve healthcare efficiency and patient experiences by reducing unnecessary ambulance transports to hospitals, specifically to distant MTCs (12). Incorporating clinical decision tools, such as the CCHR, into prehospital environment can enhance non-conveyance practices and improve ambulance response times. This adoption could enable paramedics to more efficiently identify patients with less serious head injuries who might not require urgent hospital care, thereby optimising the use of hospital resources.

For peer review only

Implementing such a strategy is anticipated to not only improve ambulance response times for other emergencies but also contribute to considerable healthcare cost savings.

To obtain reliable estimates of the diagnostic accuracy of the CCHR, a large, multi-centre study is warranted. This would be expensive and time consuming; however, the findings of such a study could inform changes to clinical practice. Successful delivery of such research would depend heavily upon adherence to the study protocol and the rates of recruitment attrition. Before embarking on such a study, it is necessary to evaluate the viability of the intended study processes (13).

The purpose of this study is to establish the feasibility of a fully powered diagnostic-test accuracy study to evaluate the accuracy of the CCHR when applied by paramedics to patients with mild TBI in the prehospital setting.

Research question

Is it feasible to conduct a prospective clinical study to evaluate the diagnostic accuracy of the CCHR in the prehospital field to assist with making informed decisions regarding the appropriate level of care for patients with minor head injuries?

Objectives

- To determine the feasibility of a study to evaluate the diagnostic accuracy of the CCHR in the prehospital environment.
- To evaluate the inter-observer reliability of the CCHR between prehospital clinicians and ED physicians.

For peer review only

- To evaluate the clinical sensibility of using the CCHR in the prehospital environment and to measure paramedics' attitudes toward the CCHR.
- To obtain an estimate of the diagnostic accuracy of the CCHR when used in the prehospital setting.

Methods

Study design and setting

A prospective, multi-centre feasibility study will be conducted in Greater Manchester, a region within the service area of the North West Ambulance Service (NWAS). NWAS is a National Health Service (NHS) trust provides emergency medical services and patient transportation across the North West region of England. It covers an extensive area of over 5,400 square miles, providing emergency and non-emergency medical services to a population of over 7.5 million people. There are two adults MTCs in Greater Manchester, which are part of the network covered by NWAS. These are Salford Royal Hospital and Manchester Royal Infirmary. In addition to these MTCs, the network also includes several trauma units and local emergency hospitals, each playing a distinct role in the trauma care pathway. The study protocol has been prospectively registered through the ISRCTN registry (study ID: ISRCTN92566288).

For the purpose of the study, we modified the CCHR for prehospital use (Table 1). The original rule excluded patients <16 years, patients with a bleeding disorder or users of oral anticoagulants and/or antiplatelet and or those experiencing a post-traumatic seizure. In the modified version, only patients under the age of 16 years will be excluded. However, patients taking anticoagulant or antiplatelet drugs will be included and will be considered 'positive' with the modified CCHR (i.e., would require transport to hospital). Anticoagulant use is defined as

For peer review only

the use of warfarin, edoxaban, dabigatran, apixaban, and rivaroxaban. Antiplatelets use is defined as the use of clopidogrel, ticlopidine, ticagrelor, dipyridamole and prasugrel. Figure 1 displays a visual representation of the recruitment process.

Participants

Patient eligibility

Inclusion criteria:

This study will be conducted using a convenience sample of adult patients with mild TBI who will be transported to Northern Care Alliance NHS Foundation Trust and Manchester University Hospital NHS Foundation Trust. Only those patients who meet the following criteria will be included:

- Adult patients (≥16 years) who receive an emergency ambulance response for a primary complaint of head injury.
- Glasgow Coma Scale (GCS) score of 13–15 at the time of assessment by attending paramedics.
- Patients transported to the hospital for clinical care.

Exclusion criteria:

- Secondary transfers patients including interfacility transport.
- Penetrating skull injury.
- Trauma to other body regions that that require clinical treatment.
- Prisoners.

For peer review only

Table1: The original and modified versions of the Canadian CT Head rule

Canadian CT head rule (original)	Canadian CT head rule (modified)
**High risk (for neurosurgical interventions)**	**High risk (for neurosurgical interventions)**
- GCS score <15 at 2 hrs after injury	- GCS score <15 at presentation
- Suspected open or depressed skull fracture	- Suspected open or depressed skull fracture
- Any sign of basal skull fracture	- Any sign of basal skull fracture
- Vomiting ≥ 2 episodes	- Vomiting ≥ 2 episodes
- Age ≥ 65 years	- Age ≥ 65 years
**Medium risk (for brain injury on CT)**	**Medium risk (for brain injury on CT)**
Amnesia before impact > 30 minutes	- Amnesia before impact > 30 minutes
Dangerous mechanism of injury (pedestrian struck by vehicle, ejection from vehicle, fall from >3 feet or >5 stairs.	Dangerous mechanism of injury (pedestrian struck by vehicle, ejection from vehicle, fall from >3 feet or >5 stairs.
* Patients with any positive high risk or medium risk criteria will be considered to have a 'positive' outcome (requiring transport to a hospital)	

Study duration and sample size

This is a feasibility study, in which evaluating recruitment rate is an outcome; therefore, it is not possible to specify a sample size a priori. However, we intend to recruit participants for a fixed period of six months. Based on data routinely collected by the Manchester University NHS Foundation Trust, a total of 5,332 patients arrived in the ED by ambulance with a primary complaint of head injury over a period of four years and nine months up to November 2020. We intend to recruit participants from two similarly sized MTCs. We anticipate that approximately 1,100 patients will be eligible to participate in this study during the recruitment period. Accounting for the availability of trained paramedics on the study delegation log and

For peer review only

concurrent injuries to other body systems that would exclude patients from participating in this study, approximately 100 participants can be recruited to this study. This number of participants is generally accepted as being appropriate for studies that focus on assessing feasibility and acceptability (14).

Outcomes

We will study the following feasibility outcomes:

- The number of eligible patients who are approached.
- The proportion of those approached who consent to participate in the study.
- The completeness of data collection.
- The completeness of follow-up.
- Determination of the inter-observer reliability of the CCHR completed by prehospital care providers and ED physicians.
- Determination of the inter-observer reliability for each component of the CCHR assessed by prehospital care providers and ED physicians.
- The diagnostic performance and clinical sensibility of the CCHR in the opinion of attending paramedics.
- Determine the acceptability of trial processes as perceived by paramedics and patients.

Additionally, as a preliminary evaluation of the diagnostic accuracy of the CCHR, the presence of traumatic intracranial lesions requiring neurosurgical intervention will be examined. This includes any neurosurgical intervention and/or abnormal head CT findings and/or death within seven days resulting from TBI.

For peer review only

Measuring and defining outcomes

Feasibility outcomes will be assessed using screening logs and case report forms completed by attending paramedics during the course of the study. At the end of the study period, a minimum of two clinicians (affiliated with the relevant NHS trusts) will review in-hospital patient records to diagnose the secondary outcomes of this feasibility study (neurosurgical significant TBI, abnormal CT findings and death within seven days). A third independent investigator will be invited to adjudicate any discrepancies. Diagnostic adjudication will be blinded to the final interpterion of the CCHR. Additionally, a neuroradiology consultant/expert will be consulted in case of the CT scan diagnosis is inconclusive.

Neurosurgical interventions are defined as any neurosurgical intervention including intracranial pressure monitoring or an Abbreviated Injury Scale (AIS) with the severity ≥3 in the head region. Abnormal head CT findings will be classified as normal or abnormal and are defined as at least one of the following: skull fractures, intracranial lesions, such as cerebral contusion and traumatic intracranial haemorrhages, or other clinically significant findings requiring hospital admission, neurosurgical interventions and routine follow-up.

Data collection

Prehospital setting

During the study period, all patients will receive standard prehospital care in accordance with local guidelines. Prior to their participation in the study, all participating paramedics will be provided with bespoke training specifically designed for the study. This training will cover all aspects of the study protocol, including eligibility assessment, the consent process, data collection procedures, and guidance on interpreting the CCHR. To ensure accessibility and

For peer review only

flexibility, the training will be offered both face-to-face and through various online sessions conducted via Zoom. Additionally, the training sessions have been recorded and made available online for those unable to attend live. This approach allows paramedics to access the training material at their convenience, ensuring that all participants receive comprehensive training on the study protocols and data collection methods.

Prehospital data will be collected using Research Electronic Data Capture (REDCap), a secure web-based platform hosted at the University of Manchester. REDCap is a software application that was created to facilitate the process of collecting and managing research data. Prior to recruitment, a training session will be held to equip participating sites with data collection skills. In addition, paper case report forms (CRFs) will be available as an alternative method of data collection. It is expected that this approach will provide flexibility and ensure continuity in data collection. A variety of data will be collected in the prehospital setting, including, but not limited to:

- Baseline demographics information (i.e, gender - age)
- Anticoagulant use.
- Injury characteristics (i.e., mechanism of injury, place).
- Past medical history.
- Initial chief complaint (i.e., vomiting, headache, loss of consciousness, amnesia).
- Prehospital physiological parameters (i.e., GCS, vital signs).
- Clinical criteria required to use the CCHR.
- Destination hospital.

In-hospital environment

REDCap will be utilised to collect hospital data, and a paper CRF will also be available as an alternative. Emergency physicians at the receiving hospital will assess the patients to determine

For peer review only

whether a CT scan is necessary in accordance with local and national guidelines. Relevant data about imaging studies undertaken and their findings will be extracted from hospital records. The routine radiology reports will be used to extract the follow-up data required for this study. Where there is uncertainty about the interpretation of the radiology report, a second radiologist will be asked to interpret the images.

Further, emergency physicians at the receiving hospitals will be asked to assess the patients using the CCHR and record their findings, blinded to the assessments made by participating paramedics. These data will be used to determine the inter-observer reliability of the CCHR between paramedics in the prehospital environment and clinicians in the ED. Additionally, in-hospital medical records will be reviewed to obtain information regarding neurosurgical interventions undertaken during the patient's hospital admission.

Follow-up

Following the initial presentation to the ED, patients will be followed up to the time of hospital discharge. Data will be collected from the clinical records of the patient, including, but not limited to

- GCS on arrival in hospital.
- Details of the clinician's assessment of the CCHR.
- Outcome of any brain imaging (i.e., CT and/or MR scans, recorded as part of routine practice).
- AIS in relation to head injury.
- Injury Severity Score.
- Mortality status.
- Neurosurgical procedures and/or surgery.

For peer review only

Evaluation of acceptability

We will use a qualitative approach to evaluate the acceptability of trial processes used to study participants. We will purposively sample (a) study participants and (b) paramedics. The intention is to maximise heterogeneity (according to age, sex and ethnic origin, and for paramedics, seniority and base ambulance station). Patients and participating paramedics will be interviewed for the purpose of exploring their experiences of participating in a study of this nature. Previous emergency care research has demonstrated the value of this method in providing information regarding the feasibility and design of a larger study (15).

To maximise convenience for participants and thereby recruitment, participants will be invited to take part in a semi-structured interview either by telephone, face-to-face or video conference. Informed consent will be obtained from all participants. We will explore the factors that facilitate or discourage participation in the research. Paramedics and study participants will be offered a £30 high street voucher to thank them for taking part. Telephone and sign language interpreters will be provided where necessary. The interview topic guides (Appendix 1) consist of a number of questions and prompts concerning the patients and paramedics' experiences of participating in this study, as well as the paramedics' perceptions of the viability of the CCHR. Prior to commencement of the interviews, two pilot interviews will be carried out. To determine the relevance, clarity and duration required to complete each interview, one will be with a paramedic and the other will be with a patient (16).

The Ottawa Acceptability of Decision Rules Instrument (OADRI) is a 12-item instrument that will be used to measure paramedics' attitudes toward the CCHR and clinical sensibility (17). Paramedics will be asked to provide their level of agreement with twelve statements about the

For peer review only

CCHR using a six-point Likert scale. The first seven statements are worded in such a way that a higher number indicates higher acceptance, while the last five are worded in the opposite manner to minimise the acquiescence bias (18).

Consent process

An exception to the normal process of informed consent is established for patients in emergency situations who are unable to provide consent. As stated in the Declaration of Helsinki, "for a research subject who is legally incompetent, physically or mentally incapable of giving consent or is a legally incompetent minor, the investigator must obtain informed consent from the legally authorised representative in accordance with applicable law. These groups should not be included in research unless the research is necessary to promote the health of the population represented and this research cannot instead be performed on legally competent persons" (19).

In this study, we will include patients in the prehospital environment who are receiving an emergency ambulance response having sustained a head injury. The nature of this presentation means that mental capacity to provide informed consent for participation in research will be compromised, especially given the time critical nature of the emergency medical response. Therefore, we will adopt a deferred consent model, which is widely used in research of this nature (20).

Paramedics will enrol eligible participants, enabling them to collect the required data. Patient care will be unchanged. We will then seek deferred written informed consent after the patients have received their initial treatment in hospital, and once they have regained their mental capacity.

For peer review only

When seeking written informed consent, a participant information sheet will be provided. Deferred consent may be obtained by either of the following means: (a) a member of staff may seek written informed consent at the receiving hospital; or (b) deferred consent may be sought in the community by post (written informed consent), email or telephone. While seeking deferred consent, staff will be available to answer any questions that the participants may have.

For participants lacking capacity, we will seek consent from their relative or legally authorised representative, which is known as proxy consent (21). A research nurse will briefly explain to them that the patient will receive the usual hospital care for TBI. The responsible nurse will also clarify that only basic clinical data will be reported using a case report form to be analysed to improve prehospital care. A brief information sheet will be provided upon request. The ability of participants to provide consent can be assessed at any point during the study period. Participants who regain capacity will be provided with a detailed participant information sheet and their written consent to continue in this feasibility study will be obtained. If the patient does not regain capacity, we will seek deferred assent from a relative or professional legal representative to retain the patient's data in the study.

Data analysis

Quantitative data

A descriptive analysis will be conducted based on the baseline data for patients enrolled in the study to characterise the demographic and clinical profile. This will include the number of eligible participants who were approached, the proportion that consented to participate, data completeness (expressed as number and percentage) for each variable collected and number

For peer review only

and percentage of participants completing follow-up. We will also report recruitment data as rates (number of participants enrolled per week across the study period).

The inter-observer reliability between paramedics and emergency physicians' use of the CCHR will be calculated using Cohen's Kappa, a statistical method for measuring agreement between two observers, together with 95% confidence intervals. Additionally, we will report upon the CCHR's diagnostic performance for ruling out clinically significant TBI. This will be achieved by calculating the sensitivity, specificity, positive and negative likelihood ratios with 95% confidence intervals against a reference standard of traumatic intracranial lesion, which will be identified from in-hospital records. Given the nature of this feasibility study, the analyses will be viewed as exploratory, preliminary and will be descriptive in nature. Furthermore, to determine the acceptability of the CCHR, the OADRI average item scores will be calculated, with higher scores indicating greater acceptability.

Qualitative data

The six-stage thematic analysis approach will be employed to analyse all interviews, as suggested by Braun and Clarke (22). This involves becoming familiar with the data, generating initial codes, searching for themes, reviewing themes, defining and naming themes and sub-themes, and producing the report. We will adhere to the four criteria for trustworthiness established by Lincoln and Guba: credibility, dependability, confirmability and transferability (23). Each step of the qualitative data collection and analysis will be guided by a qualitative research expert. The qualitative data will be managed and analysed using NVivo 12.

Progression criteria

This feasibility study will be overseen and governed by the Trial Steering Committee (TSC), which will include the chief investigator, principal investigator, two lay members, topic experts

For peer review only

in TBI and one paramedic. The role of the TSC is to provide external oversight and to ensure that the study is conducted in compliance with Good Clinical Practice guidelines (24, 25).

The traffic light approach (green/amber/red), also known as a (stop-amend-go) rule, will be used to determine whether or not to proceed with a full definitive trial (26). Green (go) indicates that the study meets the pre-set criteria, and that it would be feasible for a full-powered definitive trial to be conducted without any modifications to the study protocol. Amber (amend) indicates that further feasibility work or modifications to the protocol are required before proceeding with the full trial. Red (stop) indicates that proceeding to a definitive study is not recommended. Taking into account the feasibility outcomes, as well as qualitative interviews with patients and paramedics, a decision will be made regarding whether or not to proceed with the trial or whether to make any amendments to the study design. The progression criteria are shown in Table 2.

Patient and public involvement

It has become increasingly apparent that involving patients and the public in healthcare research can enhance the development and implementation of clinical studies, the recruitment of participants, and the relevance of study topics and outcomes in relation to their needs and experiences (27, 28). It will be a priority to take into account the perspectives of patients and the public during the design, conduct, reporting and dissemination of the research. To ensure maximum clinical impact, the study has been discussed with patient and public representatives through the NIHR Brain Injury MedTech Co-operative. A comprehensive and in-depth discussion was conducted regarding the study procedures, including key aspects such as the consent process and patient-facing materials. Two members have agreed to join the study steering committee, which is expected to meet two or three times during the study period to advise on the progress of the research, discuss any adverse events, proposed protocol

For peer review only

amendments and other matters relating to research governance. Additionally, a lay summary will be developed in collaboration with the lay members to disseminate the study findings.

Table 2: Progression criteria to proceed with a definitive trial.

	**Contributing data**	**Green (Proceed to a definitive trial)**	**Amber (Proceed to a definitive trial following some changes to the protocol**	**Do not proceed to a definitive trial**
**Screening**	Training and delegation logs	At least 20 paramedics at participating ambulance hubs trained and on delegation logs	At least 10 paramedics at participating ambulance hubs trained and on delegation logs	Less than 10 paramedics at participating ambulance hubs trained and on delegation logs
**Recruitment acceptability**	Screening & recruitment log	≥ 80% of eligible screened patients are consented to take part	≥ 60% of eligible screened patients are consented to take part	< 60% of eligible screened patients are consented to take part
**Retention**	Screening & recruitment log	Retention of ≥ 90% of study participants until study completion	Retention of ≥ 80% of study participants until study completion	Retention of < 80% of study participants until study completion
**Study procedures**	Participation data	At least 85% of the outcome data is collected (e.g., case report forms)	A minimum of 60% of the outcome data is collected (e.g., case report forms)	< 60% of the outcome data is collected (e.g., case report forms)
**Adverse events**	Participation data	No or very minor adverse events and no participants discontinued the study	Minor or serious adverse events leading to 10% or less participants discontinuing the study	Serious adverse events leading to >10% of participants discontinuing the study
**Acceptability**	Participant interview Paramedics questionnaire and interviews	Responses to OADRI* all 'agree' or 'strongly agree'. No major barriers to acceptability/participation identified during interviews with paramedics and patients	Responses to OADRI mainly 'agree' or 'strongly agree'. Some barriers to participation identified during interviews but remediable action identified.	Responses to OADRI mainly 'disagree' or 'strongly disagree'. Major barriers to acceptability/participation identified during interviews with no clear remediable action.

* OADRI: Ottawa Acceptability of Decision Rules Instrument

For peer review only

Safety considerations, adverse events and serious adverse events

Given the nature of this study, any risk to participants is very low. It is important to note that this study is observational in nature: a patient's participation in this study will have no effect on the quality of medical care they usually receive in both prehospital and hospital settings. Our study will have no impact on the decision to undergo a CT scan as this will be undertaken at the discretion of the treating clinician, as part of routine clinical care, in accordance with current national guidance. The study will also have no effect on the decision to transport patients to hospital. There is also a potential risk of data-sharing to be considered. There are small risks associated with data management, but we have carefully considered how data will be handled during the study and we will operate in accordance with current legislation and the standard operating procedures of the sponsor organisation to mitigate that risk. We are not aware of any potential risks this study would pose to the researchers.

Because of the low-risk nature of the study, we do not anticipate any adverse events (including serious adverse events) as a result of participating in this research. However, given the patient group (patients with TBI) we anticipate that many patients will experience expected adverse events, which are related to their initial injury. We will not ask sites to report expected adverse events. Expected serious adverse events include, for example, death due to head injury; neurosurgical procedures for TBI; admission to hospital for traumatic brain injury; loss of function due to TBI. Unexpected adverse events and serious adverse events should be reported.

Impact

It is anticipated that the feasibility study will provide valuable insight into whether conducting a full trial in the future is warranted. It will also provide guidance to enhance the study design,

For peer review only

recruitment plan, and consent process. If feasibility is established, we will prepare to run a fully powered multi-centre study to evaluate the diagnostic accuracy of using the CCHR in the prehospital field. This will also involve drafting a proposal to apply for research funding.

The outcomes relating to diagnostic accuracy will be of particular relevance given our knowledge that this is the first study investigating the feasibility of using the CCHR in the prehospital environment. Therefore, it is anticipated that the findings of this feasibility study will be used to inform design of a fully powered diagnostic test accuracy study. The findings of this study will be complemented by the findings of mixed methods research that we are also conducting which will explore facilitators and barriers to implementing new diagnostic pathways in the prehospital environment as perceived by practising paramedics (29).

Ethics and dissemination

Ethical approval to conduct this feasibility study has been obtained from the Health Research Authority and the Research Ethics Committee (REC reference: 22/NW/0358). To ensure privacy and confidentiality, all records and files relating to the study will be kept in a secure location and will only be accessible to members of the research team. The results will be published in peer-reviewed journals, presented at relevant national and international conferences, and will be incorporated into a doctoral thesis (NA). The study findings will also be disseminated through presentations at relevant academic forums.

For peer review only

Declarations

Ethics approval and consent to participate

The nature of this study requires ethical approval from the Health Research Authority and Research Ethics Committee, which has been obtained (REC reference: 22/NW/0358).

Consent for publication

Not applicable.

Availability of data and material

Not applicable.

Competing interests

Richard Body has consulted for Abbott Point of Care and is undertaking contract clinical research with BrainBox, which manufactures assays for biomarkers of TBI.

Funding

This work is supported by a PhD scholarship awarded to NA by the Saudi Arabian Cultural Bureau in the UK and King Saud University (KSU1872). The funders had no role in the design of this study protocol. The publication fee is funded by the cardiovascular department, University of Manchester, UK. No grant or award number.

Authors contributions

NA collaborated with the study team (SB, SC, FL, and RB) in preparing the initial study protocol. The protocol was developed through collective input and discussions. NA took the lead in drafting the manuscript, working closely with SB, SC, FL, and RB to ensure its accuracy and completeness. The final version of the manuscript was approved by all authors.

For peer review only

Acknowledgements

Not applicable.

Author affiliations

[1]Division of Cardiovascular Sciences, University of Manchester, Manchester, UK. [2]Department of Accidents and Trauma, Prince Sultan bin Abdulaziz College for Emergency Medical Services, King Saud University, Riyadh, Saudi Arabia. [3]Medical Directorate, North West Ambulance Service NHS Trust, Bolton, BL1 5DD, UK. [4] Emergency Department, Manchester University NHS Foundation Trust, Manchester, UK. [5]University of Sheffield, School of Health and Related Research, Sheffield, UK.

For peer review only

References

1. Berkner J, Mannix R, Qiu J. Clinical Traumatic Brain Injury in the Preclinical Setting. Methods Mol Biol. 2016;1462:11-28.
2. Majdan M, Plancikova D, Brazinova A, Rusnak M, Nieboer D, Feigin V, et al. Epidemiology of traumatic brain injuries in Europe: a cross-sectional analysis. Lancet Public Health. 2016;1(2):e76-e83.
3. Anandalwar SP, Mau CY, Gordhan CG, Majmundar N, Meleis A, Prestigiacomo CJ, et al. Eliminating unnecessary routine head CT scanning in neurologically intact mild traumatic brain injury patients: implementation and evaluation of a new protocol. J Neurosurg. 2016;125(3):667-73.
4. Decuypere M, Klimo P, Jr. Spectrum of traumatic brain injury from mild to severe. Surg Clin North Am. 2012;92(4):939-57, ix.
5. Haydel MJ, Preston CA, Mills TJ, Luber S, Blaudeau E, DeBlieux PM. Indications for computed tomography in patients with minor head injury. N Engl J Med. 2000;343(2):100-5.
6. Stiell IG, Lesiuk H, Wells GA, McKnight RD, Brison R, Clement C, et al. The Canadian CT Head Rule Study for patients with minor head injury: rationale, objectives, and methodology for phase I (derivation). Ann Emerg Med. 2001;38(2):160-9.
7. Stiell IG, Lesiuk H, Wells GA, Coyle D, McKnight RD, Brison R, et al. Canadian CT head rule study for patients with minor head injury: methodology for phase II (validation and economic analysis). Ann Emerg Med. 2001;38(3):317-22.
8. Stiell IG, Clement CM, Rowe BH, Schull MJ, Brison R, Cass D, et al. Comparison of the Canadian CT Head Rule and the New Orleans Criteria in patients with minor head injury. Jama. 2005;294(12):1511-8.
9. Papa L, Stiell IG, Clement CM, Pawlowicz A, Wolfram A, Braga C, et al. Performance of the Canadian CT Head Rule and the New Orleans Criteria for predicting any traumatic intracranial injury on computed tomography in a United States Level I trauma center. Acad Emerg Med. 2012;19(1):2-10.
10. Bouida W, Marghli S, Souissi S, Ksibi H, Methammem M, Haguiga H, et al. Prediction value of the Canadian CT head rule and the New Orleans criteria for positive head CT scan and acute neurosurgical procedures in minor head trauma: a multicenter external validation study. Ann Emerg Med. 2013;61(5):521-7.
11. Bonney PA, Briggs A, Briggs RG, Jarvis CA, Attenello F, Giannotta SL. Rate of Intracranial Hemorrhage After Minor Head Injury. Cureus. 2020;12(9):e10653.
12. O'Cathain A, Knowles E, Bishop-Edwards L, Coster J, Crum A, Jacques R, et al. Understanding variation in ambulance service non-conveyance rates: a mixed methods study. Health Services and Delivery Research. 2018.
13. Arain M, Campbell MJ, Cooper CL, Lancaster GA. What is a pilot or feasibility study? A review of current practice and editorial policy. BMC medical research methodology. 2010;10(1):1-7.
14. Teresi JA, Yu X, Stewart AL, Hays RD. Guidelines for Designing and Evaluating Feasibility Pilot Studies. Med Care. 2022;60(1):95-103.

For peer review only

15. van den Berg P, Kendal S, Alderson HV, Body R. An exploration of patients' experiences of participation in a randomised controlled trial of the Manchester Acute Coronary Syndromes (MACS) decision rule. Emerg Med J. 2017;34(9):593-8.
16. Majid MAA, Othman M, Mohamad SF, Lim SAH, Yusof A. Piloting for interviews in qualitative research: Operationalization and lessons learnt. International Journal of Academic Research in Business and Social Sciences. 2017;7(4):1073-80.
17. Brehaut JC, Graham ID, Wood TJ, Taljaard M, Eagles D, Lott A, et al. Measuring acceptability of clinical decision rules: validation of the Ottawa acceptability of decision rules instrument (OADRI) in four countries. Medical Decision Making. 2010;30(3):398-408.
18. Schriesheim C, Hill K. Controlling Acquiescence Response Bias by Item Reversals: The Effect on Questionnaire Validity. Educational and Psychological Measurement - EDUC PSYCHOL MEAS. 1981;41:1101-14.
19. Association WM. World Medical Association Declaration of Helsinki: ethical principles for medical research involving human subjects. Jama. 2013;310(20):2191-4.
20. Kompanje E, Jansen T, Le Noble J, de Geus H, Bakker J. Deferred consent for inclusion of patients unable to give their consent in studies in the field of emergency medicine. Nederlands Tijdschrift Voor Geneeskunde. 2008;152(38):2057-61.
21. Kompanje EJO, Maas AIR, Hilhorst M, Slieker F, Teasdale G. Ethical considerations on consent procedures for emergency research in severe and moderate traumatic brain injury. Acta Neurochirurgica. 2005;147:633-40.
22. Braun V, Clarke V. Using thematic analysis in psychology. Qualitative research in psychology. 2006;3(2):77-101.
23. Forero R, Nahidi S, De Costa J, Mohsin M, Fitzgerald G, Gibson N, et al. Application of four-dimension criteria to assess rigour of qualitative research in emergency medicine. BMC Health Serv Res. 2018;18(1):120.
24. Daykin A, Selman L, Cramer H, McCann S, Shorter G, Sydes M, et al. What are the roles and valued attributes of a Trial Steering Committee? Ethnographic study of eight clinical trials facing challenges. Trials. 2016;17:307.
25. Harman NL, Conroy EJ, Lewis SC, Murray G, Norrie J, Sydes MR, et al. Exploring the role and function of trial steering committees: results of an expert panel meeting. Trials. 2015;16:597.
26. Avery KNL, Williamson PR, Gamble C, Connell Francischetto E, Metcalfe C, Davidson P, et al. Informing efficient randomised controlled trials: exploration of challenges in developing progression criteria for internal pilot studies. BMJ Open. 2017;7(2):e013537.
27. Price A, Albarqouni L, Kirkpatrick J, Clarke M, Liew SM, Roberts N, et al. Patient and public involvement in the design of clinical trials: an overview of systematic reviews. Journal of evaluation in clinical practice. 2018;24(1):240-53.
28. Gamble C, Dudley L, Allam A, Bell P, Goodare H, Hanley B, et al. Patient and public involvement in the early stages of clinical trial development: A systematic cohort investigation. BMJ open. 2014;4:e005234.
29. Alqurashi N, Alotaibi A, Bell S, Lecky F, Body R. Towards exploring current challenges and future opportunities relating to the prehospital triage of patients with traumatic brain injury: a mixed-methods study protocol. BMJ Open. 2023;13(3):e068555.

Figure legends:

- **Figure 1: Study flowchart**

For peer review only

For peer review only

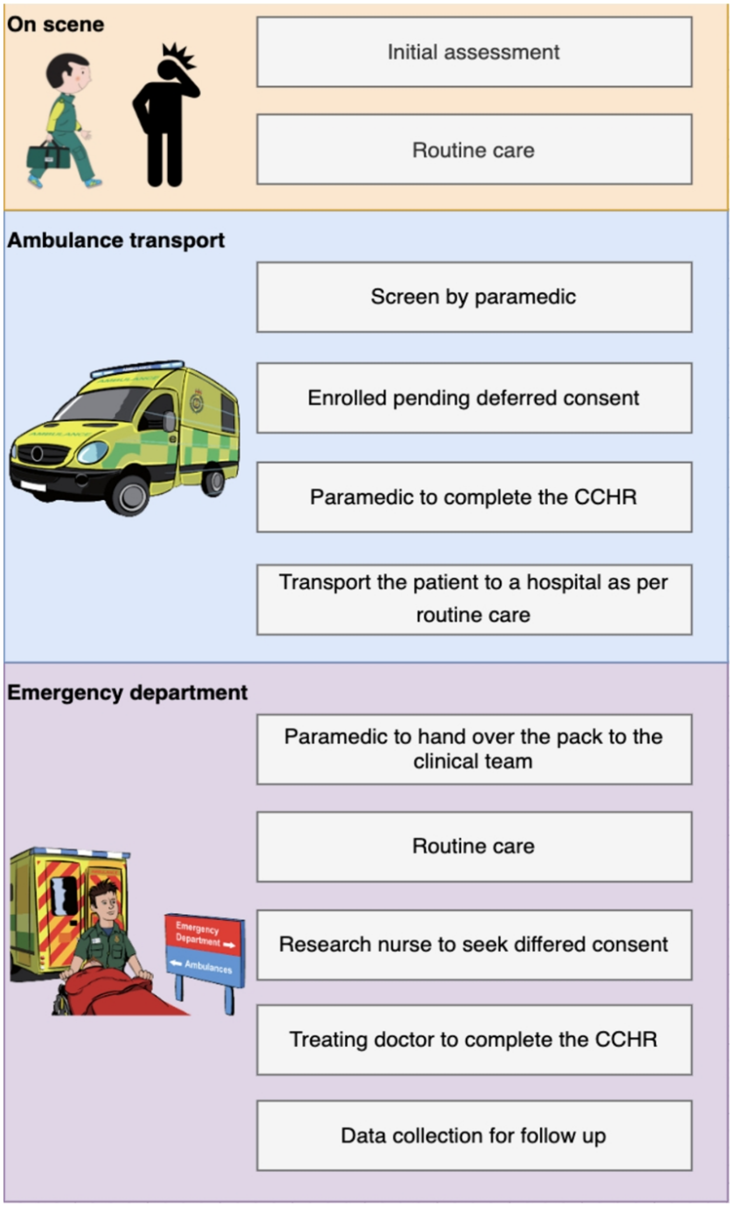

Figure 1: Study flowchart

149x247mm (144 x 144 DPI)

Head injury Evaluation and Ambulance Diagnosis: A Feasibility Study

Semi-structured interview, topic guide – Patients

Date: **Participant ID number:**

Pre-interview

- Privacy notice read.
- Consent form.
- Information sheet.

---

1. Please share your experience from the moment you were initially approached about the study.
   - **Follow-up questions, if required:**
   a. How did you feel about being approached for the study? Were there any specific factors that influenced your decision to participate?
   b. Were you given enough information to help you to decide whether to take part in this study? Was there anything that you found confusing or unclear?
   c. In this study, we didn't ask patients for permission during the ambulance journey. This is because they had experienced a head injury and were being taken to hospital as an emergency. How did you feel about this? Do you think you would have been able to make a decision about taking part when you were still in the ambulance?

2. How did you feel about the way we asked for your permission to take part in this study?
   - **Follow-up questions, if required:**
   a. Please share your overall thoughts about how we asked for your permission to take part in this study.
   b. Is there anything particularly good or bad about the way we asked for your permission to take part in the study?
   c. Do you think you were given enough time to decide whether to take part? Why or why not?

3. Can you recall any specific reasons or factors that influenced your belief that it is important to understand the study? How did this understanding affect your decision to take part in this study?

For peer review only - http://bmjopen.bmj.com/site/about/guidelines.xhtml

4. In your opinion, are there any factors that you believe might discourage people from participating in this study?

Follow-up questions, if required:

   a. Is there anything that might discourage people from participating in this study? How do you think these factors could be addressed?

5. Can you share any unexpected events or experiences that occurred during your participation in the study?

6. Would you recommend taking part in this trial to somebody else?

Follow-up questions, if required:

   a. Can you explain your thoughts and the reasons behind your recommendation or non-recommendation of participation in this study to others?
   b. In your opinion, what specific factors or aspects of the study influenced your recommendation or non-recommendation?
   c. Is there anything that you think would stop people from fully participating in this study?

7. Is there anything else you would still like to know about this study?

END

For peer review only - http://bmjopen.bmj.com/site/about/guidelines.xhtml

Head injury Evaluation and Ambulance Diagnosis: A Feasibility Study
Semi-structured interview, topic guide - Paramedics

Date: **Participant ID number:**

Pre-interview

- Privacy notice read.
- Consent form.
- Information sheet.

---

1. Firstly, I would like to know how long you have been a paramedic, and what is your highest academic qualification level?

2. Have you completed the training on the study protocol?
   a. How did you find it?
   b. Did you find the study protocol easy to understand?
   c. Was the protocol easy to remember in practice?
   d. What could be improved in the trial protocol?
3. What was the best aspect of being involved in the study?
   a. Could you tell me how easy it was to use the study case report form?

4. What would be the advantages of using the Canadian CT head Rule in the prehospital setting?
   a. Looking back to your experience, could you tell me how did you find using the Canadian CT head rule in the prehospital field?

5. What would be the disadvantages of using the Canadian CT head Rule in the prehospital setting?
   a. Looking back to your experience, did you experience any barriers/challenges to applying the Canadian CT Head Rule?

6. Would you like the Canadian CT Head Rule to become part of your practice?

7. Do you have any additional thoughts you would like to share about the study?

END

For peer review only - http://bmjopen.bmj.com/site/about/guidelines.xhtml

BMJ Open

Head Injury Evaluation and Ambulance Diagnosis (HOME) Study Protocol: A Feasibility Study Assessing the Implementation of the Canadian Computed Tomography Head Rule in the Prehospital Setting

Journal:	*BMJ Open*
Manuscript ID	bmjopen-2023-077191.R2
Article Type:	Protocol
Date Submitted by the Author:	21-Mar-2024
Complete List of Authors:	Alqurashi, Naif; The University of Manchester, Division of Cardiovascular Sciences; King Saud University, Department of Accidents and Trauma, Prince Sultan bin Abdelaziz College for Emergency Medical Services, King Saud University, Riyadh, Saudi Arabia.<br>Bell, Steve; North West Ambulance Service NHS Trust, Medical Directorate<br>Carley, Simon D; Manchester University NHS Foundation Trust, Emergency Department<br>Lecky, Fiona; University of Sheffield, School of Health and Related Research<br>Body, Richard; The University of Manchester, Division of Cardiovascular Sciences
**Primary Subject Heading**:	Emergency medicine
Secondary Subject Heading:	Evidence based practice
Keywords:	ACCIDENT & EMERGENCY MEDICINE, Feasibility Studies, TRAUMA MANAGEMENT

SCHOLARONE™ Manuscripts

Head Injury Evaluation and Ambulance Diagnosis (HOME) Study Protocol: A Feasibility Study Assessing the Implementation of the Canadian Computed Tomography Head Rule in the Prehospital Setting

Naif Alqurashi (1,2) Steve Bell (3), Simon D Carley (4), Fiona Lecky (5), Richard Body (1,4)

1. Division of Cardiovascular Sciences, University of Manchester, Manchester, UK.
2. Department of Accidents and Trauma, Prince Sultan bin Abdulaziz College for Emergency Medical Services, King Saud University, Riyadh, Saudi Arabia.
3. Medical Directorate, North West Ambulance Service NHS Trust, Bolton, BL1 5DD, UK.
4. Emergency Department, Manchester University NHS Foundation Trust, Manchester, UK.
5. University of Sheffield, School of Health and Related Research, Sheffield, UK.

***Corresponding author:** Naif Alqurashi, Division of Cardiovascular Sciences, University of Manchester, 46 Grafton Street, Manchester, M13 9PL, United Kingdom; E-mail: nalqurashi1@ksu.edu.sa

Key words: Prehospital; Traumatic brain injury; Triage; Trauma centre; Feasibility study

Word count: 4611 words, excluding the abstract, strengths and limitations, tables and the declarations.

For peer review only

Abstract

Introduction

Traumatic brain injury (TBI) is a common presentation in the prehospital environment. At present, paramedics do not routinely use tools to identify low-risk patients who could be left at scene or taken to a local hospital rather than a major trauma centre. The Canadian Computed Tomography Head Rule (CCHR) was developed to guide the use of computed tomography imaging in hospital. It has not been evaluated in the prehospital setting. We aim to address this gap by evaluating the feasibility and acceptability of implementing the CCHR to patients and paramedics, and the feasibility of conducting a full-scale clinical trial of its use.

Methods and analysis

We will recruit adult patients who are being transported to an emergency department (ED) by ambulance after suffering a mild TBI. Paramedics will prospectively collect data for the CCHR. All patients will be transported to the ED, where deferred consent will be taken and the treating clinician will re-assess the CCHR, blinded to paramedic interpretation. The primary clinical outcome will be neurosurgically significant TBI. Feasibility outcomes include recruitment and attrition rates. We will assess acceptability of the CCHR to paramedics using the Ottawa Acceptability of Decision Rules Instrument. Inter-observer reliability of the CCHR will be assessed between paramedics and the treating clinician in the ED. Participating paramedics and patients will be invited to participate in semi-structured interviews to explore the acceptability of trial processes and facilitators and barriers to the use of the CCHR in practice. Data will be analysed thematically. We anticipate recruiting approximately 100 patients over six months.

For peer review only

Ethics and dissemination

This study was approved by the Health Research Authority and the Research Ethics Committee (REC reference: 22/NW/0358). The results will be published in a peer-reviewed journal, presented at conferences, and will be incorporated into a doctoral thesis.

For peer review only

Strengths and limitations of this study

- To our knowledge, this study will be the first to evaluate the feasibility and acceptability of implementing the Canadian CT Head Rule (CCHR) in the prehospital setting, filling an important gap in the existing literature.
- The study has a limited sample size, as its primary objective is to assess the acceptability and feasibility of implementing the CCHR in the prehospital setting.
- The findings of this study will be used in the future to inform the design of a fully powered diagnostic test accuracy study.

For peer review only

Introduction

Traumatic brain injury (TBI) is widely recognised as being one of the most common causes of death and disability especially among young adults (1); however, it is becoming more frequent in the older population (2). TBI is a heterogeneous pathophysiological condition that varies widely in terms of its aetiology, severity, clinical presentation and outcomes. This broad spectrum of severity creates clinical challenges, as many patients with mild TBI may be admitted to a major trauma centre (MTC), leading to the inappropriate allocation of MTC resources for minor trauma (3).

The goal of prehospital trauma triage tools is to allocate patients efficiently and promptly to the medical facility that is most appropriate for their medical needs and for the resources available. Most TBIs are mild in nature and can be treated at a local trauma unit or non-trauma centres (4). There is no evidence indicating that these patients should be transported to a specialist neurological centre. Therefore, accurate prehospital triage of these patients will reduce the likelihood of MTCs being overwhelmed with patients suffering from mild TBI or non-TBI injuries as well as reducing the costs that might result from over-triaging patients who are unlikely to benefit from the costly resources of an MTC.

The cranial CT scan is considered the gold standard for the emergency evaluation of patients with suspected intracranial injury (5). Various validated clinical tools have been developed to determine whether a CT scan is needed to rule out intracranial lesions in adult patients with mild TBI. Of these, the Canadian CT Head Rule (CCHR) was developed through a series of prospective studies, which involved thousands of patients, to determine the need for neuroimaging (6, 7). A comparison of the various clinical tools used in different countries and settings, most notably the CCHR and the New Orleans Criteria (NOC), has been conducted.

For peer review only

While there have been some variations in the results of these comparisons, the CCHR is more specific than the NOC, although both have a similar level of sensitivity (8-10). CCHR is a highly sensitive rule to assist clinicians in identifying patients with clinically significant head injuries who should undergo CT scanning (6). Many studies have found that that the CCHR has 100% sensitivity in identifying patients who require neurosurgical interventions, as well as capturing clinically significant findings on a CT scan and is more specific (with a specificity rate 36%-65%) than other clinical decision rules. (9-11).

The CCHR rule consists of five high-risk clinical factors for neurosurgical interventions and two medium-risk factors for predicting brain injury on a CT scan (6). It takes into account a number of variables related to the patient's medical history and the findings of physical examinations. Considering that paramedics have access to all the necessary data for the CCHR, it might be feasible to utilise the CCHR in the prehospital setting to promote consistency in care and optimise resource utilisation. Prehospital implementation of the CCHR could allow paramedics to transport patients to lower-level or non-trauma centres, or even (if shown to be sufficiently sensitive) safely leave patients at the scene. However, no studies have explored whether clinical decision aids can be used to rule out neurosurgically significant TBI in patients with apparently mild TBI in the prehospital field.

For peer review only

Optimising the use of healthcare resources and enhancing patient care experiences can be greatly advanced by reducing unnecessary ambulance transports to distant MTCs, when appropriate and feasible (12, 13). In England and Wales, around one million emergency department (ED) visits annually are for head injuries, with 90% being minor (GCS 13-15), and the majority of patients are transported by ambulance services (14). Integrating the CCHR into prehospital field presents a valuable opportunity to improve the triage process, allowing prehospital care providers to make informed decisions regarding the necessity of hospital

transport and the level of care required, thereby optimising healthcare resources and potentially reducing healthcare costs. This approach also opens the door to exploring alternative care pathways for such patients, ensuring they receive appropriate and timely care without overwhelming hospital resources. The application of similar clinical decision tools in other contexts, such as the use of HEART score for managing chest pain, has shown substantial benefits in reducing unnecessary hospital conveyances and associated costs, with an average reduction in healthcare costs of €717 per patient (15). This suggests a similar potential for the CCHR in TBI management.

To obtain reliable estimates of the diagnostic accuracy of the CCHR, a large, multi-centre study is warranted. This would be expensive and time consuming; however, the findings of such a study could inform changes to clinical practice. Successful delivery of such research would depend heavily upon adherence to the study protocol and the rates of recruitment attrition. Before embarking on such a study, it is necessary to evaluate the viability of the intended study processes (16).

The purpose of this study is to establish the feasibility of a fully powered diagnostic-test accuracy study to evaluate the accuracy of the CCHR when applied by paramedics to patients with mild TBI in the prehospital setting.

For peer review only

Research question

Is it feasible to conduct a prospective clinical study to evaluate the diagnostic accuracy of the CCHR in the prehospital field to assist with making informed decisions regarding the appropriate level of care for patients with minor head injuries?

Objectives

- To determine the feasibility of a study to evaluate the diagnostic accuracy of the CCHR in the prehospital environment.
- To evaluate the inter-observer reliability of the CCHR between prehospital clinicians and ED physicians.
- To evaluate the clinical sensibility of using the CCHR in the prehospital environment and to measure paramedics' attitudes toward the CCHR.
- To obtain an estimate of the diagnostic accuracy of the CCHR when used in the prehospital setting.

Methods

Study design and setting

A prospective, multi-centre feasibility study will be conducted in Greater Manchester, a region within the service area of the North West Ambulance Service (NWAS). NWAS is a National Health Service (NHS) trust provides emergency medical services and patient transportation across the North West region of England. It covers an extensive area of over 5,400 square miles, providing emergency and non-emergency medical services to a population of over 7.5 million people. There are two adults MTCs in Greater Manchester, which are part of the network covered by NWAS. These are Salford Royal Hospital and Manchester Royal Infirmary. In addition to these MTCs, the network also includes several trauma units and local emergency hospitals, each playing a distinct role in the trauma care pathway. The study protocol has been prospectively registered through the ISRCTN registry (study ID: ISRCTN92566288).

For peer review only

For the purpose of the study, we modified the CCHR for prehospital use (Table 1). The original rule excluded patients <16 years, patients with a bleeding disorder or users of oral anticoagulants and/or antiplatelet and or those experiencing a post-traumatic seizure. In the modified version, only patients under the age of 16 years will be excluded. However, patients taking anticoagulant or antiplatelet drugs will be included and will be considered 'positive' with the modified CCHR (i.e., would require transport to hospital). Anticoagulant use is defined as the use of warfarin, edoxaban, dabigatran, apixaban, and rivaroxaban. Antiplatelets use is defined as the use of clopidogrel, ticlopidine, ticagrelor, dipyridamole and prasugrel. Figure 1 displays a visual representation of the recruitment process. At the time of writing in March 2024, the study is actively in the recruitment phase and open for participant enrolment.

Participants

Patient eligibility

Inclusion criteria

This study will be conducted using a convenience sample of adult patients with mild TBI who will be transported to Northern Care Alliance NHS Foundation Trust and Manchester University Hospital NHS Foundation Trust. Only those patients who meet the following criteria will be included:

- Adult patients (≥16 years) who receive an emergency ambulance response for a primary complaint of head injury. This includes those involved in Motor Vehicle Crashes (MVCs), provided they meet the other inclusion criteria.
- Glasgow Coma Scale (GCS) score of 13–15 at the time of assessment by attending paramedics.
- Patients transported to the hospital for clinical care.

For peer review only

Exclusion criteria

- Secondary transfers patients including interfacility transport.
- Penetrating skull injury.
- Trauma to other body regions that require clinical treatment, indicating multisystem trauma. This includes patients involved in MVCs where the treating paramedic assesses the presence of injuries beyond isolated head trauma that require clinical intervention. Paramedics will apply their clinical judgment to identify and exclude such cases.
- Prisoners.

Table1: The original and modified versions of the Canadian CT Head rule

**Canadian CT head rule (original)**	**Canadian CT head rule (modified)**
**High risk (for neurosurgical interventions)**	**High risk (for neurosurgical interventions)**
- GCS score <15 at 2 hrs after injury	- GCS score <15 at presentation
- Suspected open or depressed skull fracture	- Suspected open or depressed skull fracture
- Any sign of basal skull fracture	- Any sign of basal skull fracture
- Vomiting ≥ 2 episodes	- Vomiting ≥ 2 episodes
- Age ≥ 65 years	- Age ≥ 65 years
**Medium risk (for brain injury on CT)**	**Medium risk (for brain injury on CT)**
Amnesia before impact > 30 minutes	- Amnesia before impact > 30 minutes
Dangerous mechanism of injury (pedestrian struck by vehicle, ejection from vehicle, fall from >3 feet or >5 stairs.	Dangerous mechanism of injury (pedestrian struck by vehicle, ejection from vehicle, fall from >3 feet or >5 stairs.
* Patients with any positive high risk or medium risk criteria will be considered to have a ‘positive’ outcome (requiring transport to a hospital)	

For peer review only

Study duration and sample size

This is a feasibility study, in which evaluating recruitment rate is an outcome; therefore, it is not possible to specify a sample size a priori. However, we intend to recruit participants for a fixed period of six months. Based on data routinely collected by the Manchester University NHS Foundation Trust, a total of 5,332 patients arrived in the ED by ambulance with a primary complaint of head injury over a period of four years and nine months up to November 2020. We intend to recruit participants from two similarly sized MTCs. We anticipate that approximately 1,100 patients will be eligible to participate in this study during the recruitment period. Accounting for the availability of trained paramedics on the study delegation log and concurrent injuries to other body systems that would exclude patients from participating in this study, approximately 100 participants can be recruited to this study. This number of participants is generally accepted as being appropriate for studies that focus on assessing feasibility and acceptability (17).

Outcomes

We will study the following feasibility outcomes:

- The number of eligible patients who are approached.
- The proportion of those approached who consent to participate in the study.
- The completeness of data collection.
- The completeness of follow-up.
- Determination of the inter-observer reliability of the CCHR completed by prehospital care providers and ED physicians.
- Determination of the inter-observer reliability for each component of the CCHR assessed by prehospital care providers and ED physicians.

For peer review only

- The diagnostic performance and clinical sensibility of the CCHR in the opinion of attending paramedics.
- Determine the acceptability of trial processes as perceived by paramedics and patients.

Additionally, as a preliminary evaluation of the diagnostic accuracy of the CCHR, the presence of traumatic intracranial lesions requiring neurosurgical intervention will be examined. This includes any neurosurgical intervention and/or abnormal head CT findings and/or death within seven days resulting from TBI.

Measuring and defining outcomes

Feasibility outcomes will be assessed using screening logs and case report forms completed by attending paramedics during the course of the study. At the end of the study period, a minimum of two clinicians (affiliated with the relevant NHS trusts) will review in-hospital patient records to diagnose the secondary outcomes of this feasibility study (neurosurgical significant TBI, abnormal CT findings and death within seven days). A third independent investigator will be invited to adjudicate any discrepancies. Diagnostic adjudication will be blinded to the final interpterion of the CCHR. Additionally, a neuroradiology consultant/expert will be consulted in case of the CT scan diagnosis is inconclusive.

Neurosurgical interventions are defined as any neurosurgical intervention including intracranial pressure monitoring or an Abbreviated Injury Scale (AIS) with the severity ≥3 in the head region. Abnormal head CT findings will be classified as normal or abnormal and are defined as at least one of the following: skull fractures, intracranial lesions, such as cerebral contusion and traumatic intracranial haemorrhages, or other clinically significant findings requiring hospital admission, neurosurgical interventions and routine follow-up.

For peer review only

Data collection

Prehospital setting

During the study period, all patients will receive standard prehospital care in accordance with local guidelines. Prior to their participation in the study, all participating paramedics will be provided with bespoke training specifically designed for the study. This training will cover all aspects of the study protocol, including eligibility assessment, the consent process, data collection procedures, and guidance on interpreting the CCHR. To ensure accessibility and flexibility, the training will be offered both face-to-face and through various online sessions conducted via Zoom. Additionally, the training sessions have been recorded and made available online for those unable to attend live. This approach allows paramedics to access the training material at their convenience, ensuring that all participants receive comprehensive training on the study protocols and data collection methods.

Prehospital data will be collected using Research Electronic Data Capture (REDCap), a secure web-based platform hosted at the University of Manchester. REDCap is a software application that was created to facilitate the process of collecting and managing research data. Prior to recruitment, a training session will be held to equip participating sites with data collection skills. In addition, paper case report forms (CRFs) will be available as an alternative method of data collection. It is expected that this approach will provide flexibility and ensure continuity in data collection. A variety of data will be collected in the prehospital setting, including, but not limited to:

- Baseline demographics information (i.e, gender - age)
- Anticoagulant use.
- Injury characteristics (i.e., mechanism of injury, place).

For peer review only

- Past medical history.
- Initial chief complaint (i.e., vomiting, headache, loss of consciousness, amnesia).
- Prehospital physiological parameters (i.e., GCS, vital signs).
- Clinical criteria required to use the CCHR.
- Destination hospital.

Paramedics will record these data contemporaneously, either prior to or immediately following handover at hospital.

In-hospital environment

REDCap will be utilised to collect hospital data, and a paper CRF will also be available as an alternative. Emergency physicians at the receiving hospital will assess the patients to determine whether a CT scan is necessary in accordance with local and national guidelines. Relevant data about imaging studies undertaken and their findings will be extracted from hospital records. The routine radiology reports will be used to extract the follow-up data required for this study. Where there is uncertainty about the interpretation of the radiology report, a second radiologist will be asked to interpret the images.

Further, emergency physicians at the receiving hospitals will be asked to assess the patients using the CCHR and record their findings, blinded to the assessments made by participating paramedics. These data will be used to determine the inter-observer reliability of the CCHR between paramedics in the prehospital environment and clinicians in the ED. Additionally, in-hospital medical records will be reviewed to obtain information regarding neurosurgical interventions undertaken during the patient's hospital admission.

For peer review only

Follow-up

Following the initial presentation to the ED, patients will be followed up to the time of hospital discharge. Data will be collected from the clinical records of the patient, including, but not limited to

- GCS on arrival in hospital.
- Details of the clinician's assessment of the CCHR.
- Outcome of any brain imaging (i.e., CT and/or MR scans, recorded as part of routine practice).
- AIS in relation to head injury.
- Injury Severity Score.
- Mortality status.
- Neurosurgical procedures and/or surgery.

Evaluation of acceptability

We will use a qualitative approach to evaluate the acceptability of trial processes used to study participants. We will purposively sample (a) study participants and (b) paramedics. The intention is to maximise heterogeneity (according to age, sex and ethnic origin, and for paramedics, seniority and base ambulance station). Patients and participating paramedics will be interviewed for the purpose of exploring their experiences of participating in a study of this nature. Previous emergency care research has demonstrated the value of this method in providing information regarding the feasibility and design of a larger study (18).

To maximise convenience for participants and thereby recruitment, participants will be invited to take part in a semi-structured interview either by telephone, face-to-face or video conference.

For peer review only

Informed consent will be obtained from all participants. We will explore the factors that facilitate or discourage participation in the research. Paramedics and study participants will be offered a £30 high street voucher to thank them for taking part. Telephone and sign language interpreters will be provided where necessary. The interview topic guides (Appendix 1) consist of a number of questions and prompts concerning the patients and paramedics' experiences of participating in this study, as well as the paramedics’ perceptions of the viability of the CCHR. Prior to commencement of the interviews, two pilot interviews will be carried out. To determine the relevance, clarity and duration required to complete each interview, one will be with a paramedic and the other will be with a patient (19).

The Ottawa Acceptability of Decision Rules Instrument (OADRI) is a 12-item instrument that will be used to measure paramedics' attitudes toward the CCHR and clinical sensibility (20). Paramedics will be asked to provide their level of agreement with twelve statements about the CCHR using a six-point Likert scale. The first seven statements are worded in such a way that a higher number indicates higher acceptance, while the last five are worded in the opposite manner to minimise the acquiescence bias (21).

Consent process

An exception to the normal process of informed consent is established for patients in emergency situations who are unable to provide consent. As stated in the Declaration of Helsinki, "for a research subject who is legally incompetent, physically or mentally incapable of giving consent or is a legally incompetent minor, the investigator must obtain informed consent from the legally authorised representative in accordance with applicable law. These groups should not be included in research unless the research is necessary to promote the health

For peer review only

of the population represented and this research cannot instead be performed on legally competent persons" (22).

In this study, we will include patients in the prehospital environment who are receiving an emergency ambulance response having sustained a head injury. The nature of this presentation means that mental capacity to provide informed consent for participation in research will be compromised, especially given the time critical nature of the emergency medical response. Therefore, we will adopt a deferred consent model, which is widely used in research of this nature (23). Deferred consent in the emergency setting is a practice where medical treatment or research participation is initiated without prior consent from patients due to urgent circumstances and their compromised mental capacity, with the intention to obtain consent retrospectively once the patient's mental capacity has improved (24).

Paramedics will enrol eligible participants, enabling them to collect the required data. Patient care will be unchanged. We will then seek deferred written informed consent after the patients have received their initial treatment in hospital, and once they have regained their mental capacity.

When seeking written informed consent, a participant information sheet will be provided. Deferred consent may be obtained by either of the following means: (a) a member of staff may seek written informed consent at the receiving hospital; or (b) deferred consent may be sought in the community by post (written informed consent), email or telephone. While seeking deferred consent, staff will be available to answer any questions that the participants may have.

For participants lacking capacity, we will seek consent from their relative or legally authorised representative, which is known as proxy consent (25). A research nurse will briefly explain to

For peer review only

them that the patient will receive the usual hospital care for TBI. The responsible nurse will also clarify that only basic clinical data will be reported using a case report form to be analysed to improve prehospital care. A brief information sheet will be provided upon request. The ability of participants to provide consent can be assessed at any point during the study period. Participants who regain capacity will be provided with a detailed participant information sheet and their written consent to continue in this feasibility study will be obtained. If the patient does not regain capacity, we will seek deferred assent from a relative or professional legal representative to retain the patient's data in the study.

Data analysis

Quantitative data

A descriptive analysis will be conducted based on the baseline data for patients enrolled in the study to characterise the demographic and clinical profile. This will include the number of eligible participants who were approached, the proportion that consented to participate, data completeness (expressed as number and percentage) for each variable collected and number and percentage of participants completing follow-up. We will also report recruitment data as rates (number of participants enrolled per week across the study period).

The inter-observer reliability between paramedics and emergency physicians' use of the CCHR will be calculated using Cohen's Kappa, a statistical method for measuring agreement between two observers, together with 95% confidence intervals. Additionally, we will report upon the CCHR's diagnostic performance for ruling out clinically significant TBI. This will be achieved by calculating the sensitivity, specificity, positive and negative likelihood ratios with 95% confidence intervals against a reference standard of traumatic intracranial lesion, which will be

For peer review only

identified from in-hospital records. Given the nature of this feasibility study, the analyses will be viewed as exploratory, preliminary and will be descriptive in nature. Furthermore, to determine the acceptability of the CCHR, the OADRI average item scores will be calculated, with higher scores indicating greater acceptability.

Qualitative data

The six-stage thematic analysis approach will be employed to analyse all interviews, as suggested by Braun and Clarke (26). This involves becoming familiar with the data, generating initial codes, searching for themes, reviewing themes, defining and naming themes and sub-themes, and producing the report. We will adhere to the four criteria for trustworthiness established by Lincoln and Guba: credibility, dependability, confirmability and transferability (27). Each step of the qualitative data collection and analysis will be guided by a qualitative research expert. The qualitative data will be managed and analysed using NVivo 12.

Progression criteria

This feasibility study will be overseen and governed by the Trial Steering Committee (TSC), which will include the chief investigator, principal investigator, two lay members, topic experts in TBI and one paramedic. The role of the TSC is to provide external oversight and to ensure that the study is conducted in compliance with Good Clinical Practice guidelines (28, 29).

The traffic light approach (green/amber/red), also known as a (stop-amend-go) rule, will be used to determine whether or not to proceed with a full definitive trial (30). Green (go) indicates that the study meets the pre-set criteria, and that it would be feasible for a full-powered definitive trial to be conducted without any modifications to the study protocol. Amber (amend) indicates that further feasibility work or modifications to the protocol are required before proceeding with the full trial. Red (stop) indicates that proceeding to a definitive study is not

For peer review only

recommended. Taking into account the feasibility outcomes, as well as qualitative interviews with patients and paramedics, a decision will be made regarding whether or not to proceed with the trial or whether to make any amendments to the study design. The progression criteria are shown in Table 2.

Patient and public involvement

It has become increasingly apparent that involving patients and the public in healthcare research can enhance the development and implementation of clinical studies, the recruitment of participants, and the relevance of study topics and outcomes in relation to their needs and experiences (31, 32). It will be a priority to take into account the perspectives of patients and the public during the design, conduct, reporting and dissemination of the research. To ensure maximum clinical impact, the study has been discussed with patient and public representatives through the NIHR Brain Injury MedTech Co-operative. A comprehensive and in-depth discussion was conducted regarding the study procedures, including key aspects such as the consent process and patient-facing materials. Two members have agreed to join the study steering committee, which is expected to meet two or three times during the study period to advise on the progress of the research, discuss any adverse events, proposed protocol amendments and other matters relating to research governance. Additionally, a lay summary will be developed in collaboration with the lay members to disseminate the study findings.

For peer review only

Table 2: Progression criteria to proceed with a definitive trial.

	Contributing data	Green (Proceed to a definitive trial)	Amber (Proceed to a definitive trial following some changes to the protocol	Do not proceed to a definitive trial
**Screening**	Training and delegation logs	At least 20 paramedics at participating ambulance hubs trained and on delegation logs	At least 10 paramedics at participating ambulance hubs trained and on delegation logs	Less than 10 paramedics at participating ambulance hubs trained and on delegation logs
**Recruitment acceptability**	Screening & recruitment log	≥ 80% of eligible screened patients are consented to take part	≥ 60% of eligible screened patients are consented to take part	< 60% of eligible screened patients are consented to take part
**Retention**	Screening & recruitment log	Retention of ≥ 90% of study participants until study completion	Retention of ≥ 80% of study participants until study completion	Retention of < 80% of study participants until study completion
**Study procedures**	Participation data	At least 85% of the outcome data is collected (e.g., case report forms)	A minimum of 60% of the outcome data is collected (e.g., case report forms)	< 60% of the outcome data is collected (e.g., case report forms)
**Adverse events**	Participation data	No or very minor adverse events and no participants discontinued the study	Minor or serious adverse events leading to 10% or less participants discontinuing the study	Serious adverse events leading to >10% of participants discontinuing the study
**Acceptability**	Participant interview Paramedics questionnaire and interviews	Responses to OADRI* all 'agree' or 'strongly agree'. No major barriers to acceptability/participation identified during interviews with paramedics and patients	Responses to OADRI mainly 'agree' or 'strongly agree'. Some barriers to participation identified during interviews but remediable action identified.	Responses to OADRI mainly 'disagree' or 'strongly disagree'. Major barriers to acceptability/participation identified during interviews with no clear remediable action.

* OADRI: Ottawa Acceptability of Decision Rules Instrument

Safety considerations, adverse events and serious adverse events

Given the nature of this study, any risk to participants is very low. It is important to note that this study is observational in nature: a patient's participation in this study will have no effect

For peer review only

on the quality of medical care they usually receive in both prehospital and hospital settings. Our study will have no impact on the decision to undergo a CT scan as this will be undertaken at the discretion of the treating clinician, as part of routine clinical care, in accordance with current national guidance. The study will also have no effect on the decision to transport patients to the hospital. There are also small risks associated with data management, but we have carefully considered how data will be handled during the study and we will operate in accordance with current legislation and the standard operating procedures of the sponsor organisation to mitigate that risk. We are not aware of any potential risks this study would pose to the researchers.

Because of the low-risk nature of the study, we do not anticipate any adverse events (including serious adverse events) as a result of participating in this research. However, given the patient group (patients with TBI), we anticipate that many patients will experience expected adverse events, which are related to their initial injury. We will not ask sites to report expected adverse events. Expected serious adverse events include, for example, death due to head injury; neurosurgical procedures for TBI; admission to hospital for traumatic brain injury; loss of function due to TBI. Unexpected adverse events and serious adverse events should be reported. Adverse events will be defined in accordance with ICH Good Clinical Practice as any untoward medical occurrence in a participant, but will only be reported if this was not expected as a result of the patient's condition, up to the point of handover in hospital.

Impact

It is anticipated that the feasibility study will provide valuable insight into whether conducting a full trial in the future is warranted. It will also provide guidance to enhance the study design,

For peer review only

recruitment plan, and consent process. If feasibility is established, we will prepare to run a fully powered multi-centre study to evaluate the diagnostic accuracy of using the CCHR in the prehospital field. This will also involve drafting a proposal to apply for research funding.

The outcomes relating to diagnostic accuracy will be of particular relevance given our knowledge that this is the first study investigating the feasibility of using the CCHR in the prehospital environment. Therefore, it is anticipated that the findings of this feasibility study will be used to inform design of a fully powered diagnostic test accuracy study. The findings of this study will be complemented by the findings of mixed methods research that we are also conducting which will explore facilitators and barriers to implementing new diagnostic pathways in the prehospital environment as perceived by practising paramedics (33).

In parallel with a fully powered diagnostic test accuracy study, it will also be important to work with key stakeholders including primary care practitioners, neurosurgeons and patient and public representatives. The care pathway for patients who are not conveyed to hospital will need to be defined to ensure that patients are adequately supported with appropriate safety netting for those who experience ongoing symptoms or complications.

Ethics and dissemination

Ethical approval to conduct this feasibility study has been obtained from the Health Research Authority and the Research Ethics Committee (REC reference: 22/NW/0358). Any substantial amendment to the study protocol will need to be approved by an ethics committee after being approved by the sponsor. To ensure privacy and confidentiality, all records and files relating to the study will be kept in a secure location and will only be accessible to members of the

For peer review only

research team. The results will be published in peer-reviewed journals, presented at relevant national and international conferences, and will be incorporated into a doctoral thesis (NA). The study findings will also be disseminated through presentations at relevant academic forums.

Declarations

Ethics approval and consent to participate

The nature of this study requires ethical approval from the Health Research Authority and Research Ethics Committee, which has been obtained (REC reference: 22/NW/0358).

Consent for publication

Not applicable.

Availability of data and material

Not applicable.

Competing interests

Richard Body has consulted for Abbott Point of Care and is undertaking contract clinical research with BrainBox, which manufactures assays for biomarkers of TBI.

Funding

This work is supported by a PhD scholarship awarded to NA by the Saudi Arabian Cultural Bureau in the UK and King Saud University (KSU1872). The funders had no role in the design of this study protocol. The study is sponsored by the University of Manchester. The publication fee is funded by the cardiovascular department, University of Manchester, UK. No grant or award number.

Authors contributions

NA collaborated with the study team (SB, SC, FL, and RB) in preparing the initial study protocol. The protocol was developed through collective input and discussions. NA took the lead in drafting the manuscript, working closely with SB, SC, FL, and RB to ensure its accuracy and completeness. The final version of the manuscript was approved by all authors.

Acknowledgements

Not applicable.

Author affiliations

[1]Division of Cardiovascular Sciences, University of Manchester, Manchester, UK. [2]Department of Accidents and Trauma, Prince Sultan bin Abdulaziz College for Emergency Medical Services, King Saud University, Riyadh, Saudi Arabia. [3]Medical Directorate, North West Ambulance Service NHS Trust, Bolton, BL1 5DD, UK. [4] Emergency Department, Manchester University NHS Foundation Trust, Manchester, UK. [5]University of Sheffield, School of Health and Related Research, Sheffield, UK.

For peer review only

References

1. Berkner J, Mannix R, Qiu J. Clinical Traumatic Brain Injury in the Preclinical Setting. Methods Mol Biol. 2016;1462:11-28.
2. Majdan M, Plancikova D, Brazinova A, Rusnak M, Nieboer D, Feigin V, et al. Epidemiology of traumatic brain injuries in Europe: a cross-sectional analysis. Lancet Public Health. 2016;1(2):e76-e83.
3. Anandalwar SP, Mau CY, Gordhan CG, Majmundar N, Meleis A, Prestigiacomo CJ, et al. Eliminating unnecessary routine head CT scanning in neurologically intact mild traumatic brain injury patients: implementation and evaluation of a new protocol. J Neurosurg. 2016;125(3):667-73.
4. Decuypere M, Klimo P, Jr. Spectrum of traumatic brain injury from mild to severe. Surg Clin North Am. 2012;92(4):939-57, ix.
5. Haydel MJ, Preston CA, Mills TJ, Luber S, Blaudeau E, DeBlieux PM. Indications for computed tomography in patients with minor head injury. N Engl J Med. 2000;343(2):100-5.
6. Stiell IG, Lesiuk H, Wells GA, McKnight RD, Brison R, Clement C, et al. The Canadian CT Head Rule Study for patients with minor head injury: rationale, objectives, and methodology for phase I (derivation). Ann Emerg Med. 2001;38(2):160-9.
7. Stiell IG, Lesiuk H, Wells GA, Coyle D, McKnight RD, Brison R, et al. Canadian CT head rule study for patients with minor head injury: methodology for phase II (validation and economic analysis). Ann Emerg Med. 2001;38(3):317-22.
8. Stiell IG, Clement CM, Rowe BH, Schull MJ, Brison R, Cass D, et al. Comparison of the Canadian CT Head Rule and the New Orleans Criteria in patients with minor head injury. Jama. 2005;294(12):1511-8.
9. Papa L, Stiell IG, Clement CM, Pawlowicz A, Wolfram A, Braga C, et al. Performance of the Canadian CT Head Rule and the New Orleans Criteria for predicting any traumatic intracranial injury on computed tomography in a United States Level I trauma center. Acad Emerg Med. 2012;19(1):2-10.
10. Bouida W, Marghli S, Souissi S, Ksibi H, Methammem M, Haguiga H, et al. Prediction value of the Canadian CT head rule and the New Orleans criteria for positive head CT scan and acute neurosurgical procedures in minor head trauma: a multicenter external validation study. Ann Emerg Med. 2013;61(5):521-7.
11. Bonney PA, Briggs A, Briggs RG, Jarvis CA, Attenello F, Giannotta SL. Rate of Intracranial Hemorrhage After Minor Head Injury. Cureus. 2020;12(9):e10653.
12. O'Cathain A, Knowles E, Bishop-Edwards L, Coster J, Crum A, Jacques R, et al. Understanding variation in ambulance service non-conveyance rates: a mixed methods study. Health Services and Delivery Research. 2018.
13. Mason S, Kuczawski M, Teare MD, Stevenson M, Goodacre S, Ramlakhan S, et al. AHEAD study: an observational study of the management of anticoagulated patients who suffer head injury. BMJ open. 2017;7(1):e014324.
14. Lawrence T, Helmy A, Bouamra O, Woodford M, Lecky F, Hutchinson PJ. Traumatic brain injury in England and Wales: prospective audit of epidemiology, complications and standardised mortality. BMJ Open. 2016;6(11):e012197.
15. Aarts GWA, Camaro C, Adang EMM, Rodwell L, van Hout R, Brok G, et al. Pre-hospital rule-out of non-ST-segment elevation acute coronary syndrome by a single troponin: final

For peer review only

one-year outcomes of the ARTICA randomised trial. Eur Heart J Qual Care Clin Outcomes. 2024.
16. Arain M, Campbell MJ, Cooper CL, Lancaster GA. What is a pilot or feasibility study? A review of current practice and editorial policy. BMC medical research methodology. 2010;10(1):1-7.
17. Teresi JA, Yu X, Stewart AL, Hays RD. Guidelines for Designing and Evaluating Feasibility Pilot Studies. Med Care. 2022;60(1):95-103.
18. van den Berg P, Kendal S, Alderson HV, Body R. An exploration of patients' experiences of participation in a randomised controlled trial of the Manchester Acute Coronary Syndromes (MACS) decision rule. Emerg Med J. 2017;34(9):593-8.
19. Majid MAA, Othman M, Mohamad SF, Lim SAH, Yusof A. Piloting for interviews in qualitative research: Operationalization and lessons learnt. International Journal of Academic Research in Business and Social Sciences. 2017;7(4):1073-80.
20. Brehaut JC, Graham ID, Wood TJ, Taljaard M, Eagles D, Lott A, et al. Measuring acceptability of clinical decision rules: validation of the Ottawa acceptability of decision rules instrument (OADRI) in four countries. Medical Decision Making. 2010;30(3):398-408.
21. Schriesheim C, Hill K. Controlling Acquiescence Response Bias by Item Reversals: The Effect on Questionnaire Validity. Educational and Psychological Measurement - EDUC PSYCHOL MEAS. 1981;41:1101-14.
22. Association WM. World Medical Association Declaration of Helsinki: ethical principles for medical research involving human subjects. Jama. 2013;310(20):2191-4.
23. Kompanje E, Jansen T, Le Noble J, de Geus H, Bakker J. Deferred consent for inclusion of patients unable to give their consent in studies in the field of emergency medicine. Nederlands Tijdschrift Voor Geneeskunde. 2008;152(38):2057-61.
24. van der Graaf R, Hoogerwerf MA, de Vries MC. The ethics of deferred consent in times of pandemics. Nat Med. 2020;26(9):1328-30.
25. Kompanje EJO, Maas AIR, Hilhorst M, Slieker F, Teasdale G. Ethical considerations on consent procedures for emergency research in severe and moderate traumatic brain injury. Acta Neurochirurgica. 2005;147:633-40.
26. Braun V, Clarke V. Using thematic analysis in psychology. Qualitative research in psychology. 2006;3(2):77-101.
27. Forero R, Nahidi S, De Costa J, Mohsin M, Fitzgerald G, Gibson N, et al. Application of four-dimension criteria to assess rigour of qualitative research in emergency medicine. BMC Health Serv Res. 2018;18(1):120.
28. Daykin A, Selman L, Cramer H, McCann S, Shorter G, Sydes M, et al. What are the roles and valued attributes of a Trial Steering Committee? Ethnographic study of eight clinical trials facing challenges. Trials. 2016;17:307.
29. Harman NL, Conroy EJ, Lewis SC, Murray G, Norrie J, Sydes MR, et al. Exploring the role and function of trial steering committees: results of an expert panel meeting. Trials. 2015;16:597.
30. Avery KNL, Williamson PR, Gamble C, Connell Francischetto E, Metcalfe C, Davidson P, et al. Informing efficient randomised controlled trials: exploration of challenges in developing progression criteria for internal pilot studies. BMJ Open. 2017;7(2):e013537.
31. Price A, Albarqouni L, Kirkpatrick J, Clarke M, Liew SM, Roberts N, et al. Patient and public involvement in the design of clinical trials: an overview of systematic reviews. Journal of evaluation in clinical practice. 2018;24(1):240-53.

For peer review only

32. Gamble C, Dudley L, Allam A, Bell P, Goodare H, Hanley B, et al. Patient and public involvement in the early stages of clinical trial development: A systematic cohort investigation. BMJ open. 2014;4:e005234.
33. Alqurashi N, Alotaibi A, Bell S, Lecky F, Body R. Towards exploring current challenges and future opportunities relating to the prehospital triage of patients with traumatic brain injury: a mixed-methods study protocol. BMJ Open. 2023;13(3):e068555.

Figure legends:

- **Figure 1: Study flowchart**

For peer review only

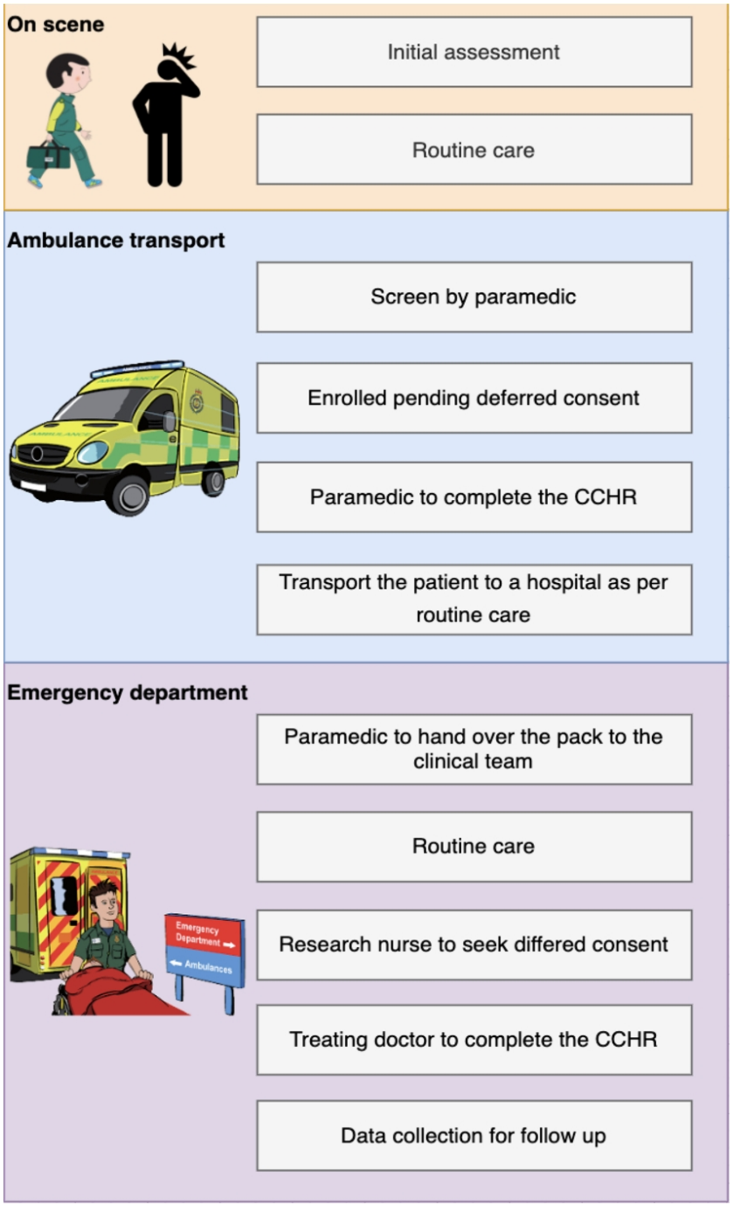

Figure 1: Study flowchart

149x247mm (144 x 144 DPI)

Head injury Evaluation and Ambulance Diagnosis: A Feasibility Study

Semi-structured interview, topic guide – Patients

Date: **Participant ID number:**

Pre-interview

- Privacy notice read.
- Consent form.
- Information sheet.

---

1. Please share your experience from the moment you were initially approached about the study.
   - **Follow-up questions, if required:**

   a. How did you feel about being approached for the study? Were there any specific factors that influenced your decision to participate?

   b. Were you given enough information to help you to decide whether to take part in this study? Was there anything that you found confusing or unclear?

   c. In this study, we didn't ask patients for permission during the ambulance journey. This is because they had experienced a head injury and were being taken to hospital as an emergency. How did you feel about this? Do you think you would have been able to make a decision about taking part when you were still in the ambulance?

2. How did you feel about the way we asked for your permission to take part in this study?
   - **Follow-up questions, if required:**

   a. Please share your overall thoughts about how we asked for your permission to take part in this study.

   b. Is there anything particularly good or bad about the way we asked for your permission to take part in the study?

   c. Do you think you were given enough time to decide whether to take part? Why or why not?

3. Can you recall any specific reasons or factors that influenced your belief that it is important to understand the study? How did this understanding affect your decision to take part in this study?

For peer review only - http://bmjopen.bmj.com/site/about/guidelines.xhtml

4. In your opinion, are there any factors that you believe might discourage people from participating in this study?

Follow-up questions, if required:

   a. Is there anything that might discourage people from participating in this study? How do you think these factors could be addressed?

5. Can you share any unexpected events or experiences that occurred during your participation in the study?

6. Would you recommend taking part in this trial to somebody else?

Follow-up questions, if required:

   a. Can you explain your thoughts and the reasons behind your recommendation or non-recommendation of participation in this study to others?
   b. In your opinion, what specific factors or aspects of the study influenced your recommendation or non-recommendation?
   c. Is there anything that you think would stop people from fully participating in this study?

7. Is there anything else you would still like to know about this study?

******END******

For peer review only

Head injury Evaluation and Ambulance Diagnosis: A Feasibility Study
Semi-structured interview, topic guide - Paramedics

Date: **Participant ID number:**

Pre-interview

- Privacy notice read.
- Consent form.
- Information sheet.

---

1. Firstly, I would like to know how long you have been a paramedic, and what is your highest academic qualification level?

2. Have you completed the training on the study protocol?
   a. How did you find it?
   b. Did you find the study protocol easy to understand?
   c. Was the protocol easy to remember in practice?
   d. What could be improved in the trial protocol?
3. What was the best aspect of being involved in the study?
   a. Could you tell me how easy it was to use the study case report form?

4. What would be the advantages of using the Canadian CT head Rule in the prehospital setting?
   a. Looking back to your experience, could you tell me how did you find using the Canadian CT head rule in the prehospital field?

5. What would be the disadvantages of using the Canadian CT head Rule in the prehospital setting?
   a. Looking back to your experience, did you experience any barriers/challenges to applying the Canadian CT Head Rule?

6. Would you like the Canadian CT Head Rule to become part of your practice?

7. Do you have any additional thoughts you would like to share about the study?

END

For peer review only - http://bmjopen.bmj.com/site/about/guidelines.xhtml

SPIRIT Checklist

		Reporting Item	Page Number
**Administrative information**			
Title	#1	Descriptive title identifying the study design, population, interventions, and, if applicable, trial acronym	1
Trial registration	#2a	Trial identifier and registry name. If not yet registered, name of intended registry	8
Trial registration: data set	#2b	All items from the World Health Organization Trial Registration Data Set	NA
Protocol version	#3	Date and version identifier	NA
Funding	#4	Sources and types of financial, material, and other support	24
Roles and responsibilities: contributorship	#5a	Names, affiliations, and roles of protocol contributors	24,25
Roles and responsibilities: sponsor contact information	#5b	Name and contact information for the trial sponsor	24
Roles and responsibilities: sponsor and funder	#5c	Role of study sponsor and funders, if any, in study design; collection, management, analysis, and interpretation of data; writing of the report; and the decision to submit the report for publication, including whether they will have ultimate authority over any of these activities	24
Roles and responsibilities: committees	#5d	Composition, roles, and responsibilities of the coordinating centre, steering committee, endpoint adjudication committee, data management team, and other individuals or groups overseeing the trial, if applicable (see Item 21a for data monitoring committee)	19
**Introduction**			

For peer review only

Background and rationale	#6a	Description of research question and justification for undertaking the trial, including summary of relevant studies (published and unpublished) examining benefits and harms for each intervention	5-7
Background and rationale: choice of comparators	#6b	Explanation for choice of comparators	NA
Objectives	#7	Specific objectives or hypotheses	8
Trial design	#8	Description of trial design including type of trial (eg, parallel group, crossover, factorial, single group), allocation ratio, and framework (eg, superiority, equivalence, non-inferiority, exploratory)	8,9
**Methods: Participants, interventions, and outcomes**			
Study setting	#9	Description of study settings (eg, community clinic, academic hospital) and list of countries where data will be collected. Reference to where list of study sites can be obtained	8,9
Eligibility criteria	#10	Inclusion and exclusion criteria for participants. If applicable, eligibility criteria for study centres and individuals who will perform the interventions (eg, surgeons, psychotherapists)	9,10
Interventions: description	#11a	Interventions for each group with sufficient detail to allow replication, including how and when they will be administered	NA
Interventions: modifications	#11b	Criteria for discontinuing or modifying allocated interventions for a given trial participant (eg, drug dose change in response to harms, participant request, or improving / worsening disease)	NA
Interventions: adherance	#11c	Strategies to improve adherence to intervention protocols, and any procedures for monitoring adherence (eg, drug tablet return; laboratory tests)	13-15
Interventions: concomitant care	#11d	Relevant concomitant care and interventions that are permitted or prohibited during the trial	NA
Outcomes	#12	Primary, secondary, and other outcomes, including the specific measurement variable (eg, systolic blood pressure), analysis	11,12

For peer review only

		metric (eg, change from baseline, final value, time to event), method of aggregation (eg, median, proportion), and time point for each outcome. Explanation of the clinical relevance of chosen efficacy and harm outcomes is strongly recommended	
Participant timeline	#13	Time schedule of enrolment, interventions (including any run-ins and washouts), assessments, and visits for participants. A schematic diagram is highly recommended (see Figure)	13-15 and Figure 1
Sample size	#14	Estimated number of participants needed to achieve study objectives and how it was determined, including clinical and statistical assumptions supporting any sample size calculations	11
Recruitment	#15	Strategies for achieving adequate participant enrolment to reach target sample size	11
**Methods: Assignment of interventions (for controlled trials)**			
Allocation: sequence generation	#16a	Method of generating the allocation sequence (eg, computer-generated random numbers), and list of any factors for stratification. To reduce predictability of a random sequence, details of any planned restriction (eg, blocking) should be provided in a separate document that is unavailable to those who enrol participants or assign interventions	NA
Allocation concealment mechanism	#16b	Mechanism of implementing the allocation sequence (eg, central telephone; sequentially numbered, opaque, sealed envelopes), describing any steps to conceal the sequence until interventions are assigned	NA
Allocation: implementation	#16c	Who will generate the allocation sequence, who will enrol participants, and who will assign participants to interventions	NA
Blinding (masking)	#17a	Who will be blinded after assignment to interventions (eg, trial participants, care providers, outcome assessors, data analysts), and how	NA
Blinding (masking): emergency unblinding	#17b	If blinded, circumstances under which unblinding is permissible, and procedure for revealing a participant's allocated intervention during the trial	NA
**Methods: Data**			

For peer review only

**collection, management, and analysis**			
Data collection plan	#18a	Plans for assessment and collection of outcome, baseline, and other trial data, including any related processes to promote data quality (eg, duplicate measurements, training of assessors) and a description of study instruments (eg, questionnaires, laboratory tests) along with their reliability and validity, if known. Reference to where data collection forms can be found, if not in the protocol	13-15
Data collection plan: retention	#18b	Plans to promote participant retention and complete follow-up, including list of any outcome data to be collected for participants who discontinue or deviate from intervention protocols	15
Data management	#19	Plans for data entry, coding, security, and storage, including any related processes to promote data quality (eg, double data entry; range checks for data values). Reference to where details of data management procedures can be found, if not in the protocol	13,14
Statistics: outcomes	#20a	Statistical methods for analysing primary and secondary outcomes. Reference to where other details of the statistical analysis plan can be found, if not in the protocol	18,19
Statistics: additional analyses	#20b	Methods for any additional analyses (eg, subgroup and adjusted analyses)	NA
Statistics: analysis population and missing data	#20c	Definition of analysis population relating to protocol non-adherence (eg, as randomised analysis), and any statistical methods to handle missing data (eg, multiple imputation)	18,19
**Methods: Monitoring**			
Data monitoring: formal committee	#21a	Composition of data monitoring committee (DMC); summary of its role and reporting structure; statement of whether it is independent from the sponsor and competing interests; and reference to where further details about its charter can be found, if not in the protocol. Alternatively, an explanation of why a DMC is not needed	19,20
Data monitoring: interim analysis	#21b	Description of any interim analyses and stopping guidelines, including who will have access to these interim results and make the final decision to terminate the trial	19,20

For peer review only

Harms	#22	Plans for collecting, assessing, reporting, and managing solicited and spontaneously reported adverse events and other unintended effects of trial interventions or trial conduct	21,22
Auditing	#23	Frequency and procedures for auditing trial conduct, if any, and whether the process will be independent from investigators and the sponsor	19
**Ethics and dissemination**			
Research ethics approval	#24	Plans for seeking research ethics committee / institutional review board (REC / IRB) approval	23
Protocol amendments	#25	Plans for communicating important protocol modifications (eg, changes to eligibility criteria, outcomes, analyses) to relevant parties (eg, investigators, REC / IRBs, trial participants, trial registries, journals, regulators)	23
Consent or assent	#26a	Who will obtain informed consent or assent from potential trial participants or authorised surrogates, and how (see Item 32)	16-18
Consent or assent: ancillary studies	#26b	Additional consent provisions for collection and use of participant data and biological specimens in ancillary studies, if applicable	NA
Confidentiality	#27	How personal information about potential and enrolled participants will be collected, shared, and maintained in order to protect confidentiality before, during, and after the trial	23,24
Declaration of interests	#28	Financial and other competing interests for principal investigators for the overall trial and each study site	24
Data access	#29	Statement of who will have access to the final trial dataset, and disclosure of contractual agreements that limit such access for investigators	24
Ancillary and post trial care	#30	Provisions, if any, for ancillary and post-trial care, and for compensation to those who suffer harm from trial participation	NA
Dissemination policy: trial results	#31a	Plans for investigators and sponsor to communicate trial results to participants, healthcare professionals, the public, and other relevant groups (eg, via publication, reporting in results databases, or other data sharing arrangements), including any publication restrictions	23,24

For peer review only

Dissemination policy: authorship	#31b	Authorship eligibility guidelines and any intended use of professional writers	NA
Dissemination policy: reproducible research	#31c	Plans, if any, for granting public access to the full protocol, participant-level dataset, and statistical code	NA
**Appendices**			
Informed consent materials	#32	Model consent form and other related documentation given to participants and authorised surrogates	
Biological specimens	#33	Plans for collection, laboratory evaluation, and storage of biological specimens for genetic or molecular analysis in the current trial and for future use in ancillary studies, if applicable	NA

For peer review only

BMJ Open

Head Injury Evaluation and Ambulance Diagnosis (HOME) Study Protocol: A Feasibility Study Assessing the Implementation of the Canadian Computed Tomography Head Rule in the Prehospital Setting

Journal:	*BMJ Open*
Manuscript ID	bmjopen-2023-077191.R3
Article Type:	Protocol
Date Submitted by the Author:	01-May-2024
Complete List of Authors:	Alqurashi, Naif; The University of Manchester, Division of Cardiovascular Sciences; King Saud University, Department of Accidents and Trauma, Prince Sultan bin Abdelaziz College for Emergency Medical Services, King Saud University, Riyadh, Saudi Arabia.<br>Bell, Steve; North West Ambulance Service NHS Trust, Medical Directorate<br>Carley, Simon D; Manchester University NHS Foundation Trust, Emergency Department<br>Lecky, Fiona; University of Sheffield, School of Health and Related Research<br>Body, Richard; The University of Manchester, Division of Cardiovascular Sciences
**Primary Subject Heading**:	Emergency medicine
Secondary Subject Heading:	Evidence based practice
Keywords:	ACCIDENT & EMERGENCY MEDICINE, Feasibility Studies, TRAUMA MANAGEMENT

SCHOLARONE™ Manuscripts

Head Injury Evaluation and Ambulance Diagnosis (HOME) Study Protocol: A Feasibility Study Assessing the Implementation of the Canadian Computed Tomography Head Rule in the Prehospital Setting

Naif Alqurashi (1,2) Steve Bell (3), Simon D Carley (4), Fiona Lecky (5), Richard Body (1,4)

1. Division of Cardiovascular Sciences, University of Manchester, Manchester, UK.
2. Department of Accidents and Trauma, Prince Sultan bin Abdulaziz College for Emergency Medical Services, King Saud University, Riyadh, Saudi Arabia.
3. Medical Directorate, North West Ambulance Service NHS Trust, Bolton, BL1 5DD, UK.
4. Emergency Department, Manchester University NHS Foundation Trust, Manchester, UK.
5. University of Sheffield, School of Health and Related Research, Sheffield, UK.

***Corresponding author:** Naif Alqurashi, Division of Cardiovascular Sciences, University of Manchester, 46 Grafton Street, Manchester, M13 9PL, United Kingdom; E-mail: nalqurashi1@ksu.edu.sa

Key words: Prehospital; Traumatic brain injury; Triage; Trauma centre; Feasibility study

Word count: 4526 words, excluding the abstract, strengths and limitations, tables and the declarations.

For peer review only

Abstract

Introduction

Traumatic brain injury (TBI) is a common presentation in the prehospital environment. At present, paramedics do not routinely use tools to identify low-risk patients who could be left at scene or taken to a local hospital rather than a major trauma centre. The Canadian Computed Tomography Head Rule (CCHR) was developed to guide the use of computed tomography imaging in hospital. It has not been evaluated in the prehospital setting. We aim to address this gap by evaluating the feasibility and acceptability of implementing the CCHR to patients and paramedics, and the feasibility of conducting a full-scale clinical trial of its use.

Methods and analysis

We will recruit adult patients who are being transported to an emergency department (ED) by ambulance after suffering a mild TBI. Paramedics will prospectively collect data for the CCHR. All patients will be transported to the ED, where deferred consent will be taken and the treating clinician will re-assess the CCHR, blinded to paramedic interpretation. The primary clinical outcome will be neurosurgically significant TBI. Feasibility outcomes include recruitment and attrition rates. We will assess acceptability of the CCHR to paramedics using the Ottawa Acceptability of Decision Rules Instrument. Inter-observer reliability of the CCHR will be assessed between paramedics and the treating clinician in the ED. Participating paramedics and patients will be invited to participate in semi-structured interviews to explore the acceptability of trial processes and facilitators and barriers to the use of the CCHR in practice. Data will be analysed thematically. We anticipate recruiting approximately 100 patients over six months.

For peer review only

Ethics and dissemination

This study was approved by the Health Research Authority and the Research Ethics Committee (REC reference: 22/NW/0358). The results will be published in a peer-reviewed journal, presented at conferences, and will be incorporated into a doctoral thesis.

For peer review only

Strengths and limitations of this study

- To our knowledge, this study will be the first to evaluate the feasibility and acceptability of implementing the Canadian CT Head Rule (CCHR) in the prehospital setting, filling an important gap in the existing literature.
- The study has a limited sample size, as its primary objective is to assess the acceptability and feasibility of implementing the CCHR in the prehospital setting.
- The findings of this study will be used in the future to inform the design of a fully powered diagnostic test accuracy study.

For peer review only

Introduction

Traumatic brain injury (TBI) is widely recognised as being one of the most common causes of death and disability especially among young adults (1); however, it is becoming more frequent in the older population (2). TBI is a heterogeneous pathophysiological condition that varies widely in terms of its aetiology, severity, clinical presentation, and outcomes. This broad spectrum of severity creates clinical challenges, such as the inappropriate allocation of major trauma centre (MTC) resources to less serious cases (3).

The goal of prehospital trauma triage tools is to efficiently and promptly direct patients to the medical facility best suited to their needs and the available resources. Most TBIs are mild in nature and can be treated at a local trauma unit or non-trauma centres (4). There is no evidence indicating that these patients should be transported to a specialist neurological centre. Therefore, accurate prehospital triage of these patients will reduce the likelihood of MTCs being overwhelmed with patients suffering from mild TBI or non-TBI injuries as well as reducing the costs that might result from over-triaging patients who are unlikely to benefit from the costly resources of an MTC.

The cranial CT scan is considered the gold standard for the emergency evaluation of patients with suspected intracranial injury (5). Various validated clinical decision tools, including the Canadian CT Head Rule (CCHR), have been developed through extensive prospective studies to assess the need for a CT scan after a minor head injury (6, 7). Comparative studies have examined various clinical tools, most notably the New Orleans Criteria (NOC) and the CCHR, revealing that although both demonstrate similar sensitivity, the CCHR generally provides greater specificity (8-10). Several studies have shown that the CCHR achieves 100% sensitivity

For peer review only

in identifying neurosurgical needs and captures significant CT findings, offering greater specificity (36%-65%) compared to other decision rules (9-11).

The National Institute for Health and Care Excellence (NICE) has guidelines that largely follow the CCHR to determine when to perform CT scans in cases of head injury (12). This approach is supported by evidence showing that the CCHR rule is the most cost-effective among various decision rules evaluated (13). Although the number of CT scans has increased, the admission rate has been halved (from 9% to 4%), resulting in a saving of £3,381 per 100 patients (14, 15). The implementation of NICE head injury guidelines indicates that the higher costs of CT scans, averaging about £100 per scan, are balanced by the savings from reduced hospital admissions, estimated to be around £847 per patient (13).

Considering that paramedics have access to all the necessary data for the CCHR, it might be feasible to utilise the CCHR in the prehospital setting to promote consistency in care and optimise resource utilisation. Prehospital implementation of the CCHR could enable paramedics to transport patients to lower-level or non-trauma centres, or even (if shown to be sufficiently sensitive) safely leave patients at the scene. However, no studies have explored whether clinical decision aids can be used to rule out neurosurgically significant TBI in patients with apparently mild TBI in the prehospital field.

In England and Wales, around one million emergency department (ED) visits annually are for head injuries, with 90% being minor (GCS 13-15), and the majority of patients are transported by ambulance services (16). Optimising healthcare resource use and enhancing patient care experiences can be greatly advanced by reducing unnecessary ambulance transports to distant MTCs, when appropriate and feasible (17, 18). Integrating the CCHR into prehospital field presents a valuable opportunity to improve the triage process, allowing prehospital care

For peer review only

providers to make informed decisions regarding the necessity of hospital transport and the level of care required, thereby optimising healthcare resources and potentially reducing healthcare costs. This approach also opens the door to exploring alternative care pathways for such patients, ensuring they receive appropriate and timely care without overwhelming hospital resources.

To obtain reliable estimates of the diagnostic accuracy of the CCHR, a large, multi-centre study is warranted. This would be expensive and time consuming; however, the findings of such a study could inform changes to clinical practice. Successful delivery of such research would depend heavily upon adherence to the study protocol and the rates of recruitment attrition. Before embarking on such a study, it is necessary to evaluate the viability of the intended study processes (19).

The purpose of this study is to establish the feasibility of a fully powered diagnostic-test accuracy study to evaluate the accuracy of the CCHR when applied by paramedics to patients with mild TBI in the prehospital setting.

Research question

Is it feasible to conduct a prospective clinical study to evaluate the diagnostic accuracy of the CCHR in the prehospital field to assist with making informed decisions regarding the appropriate level of care for patients with minor head injuries?

Objectives

- To determine the feasibility of a study to evaluate the diagnostic accuracy of the CCHR in the prehospital environment.

For peer review only

- To evaluate the inter-observer reliability of the CCHR between prehospital clinicians and ED physicians.
- To evaluate the clinical sensibility of using the CCHR in the prehospital environment and to measure paramedics' attitudes toward the CCHR.
- To obtain an estimate of the diagnostic accuracy of the CCHR when used in the prehospital setting.

Methods

Study design and setting

A prospective, multi-centre feasibility study will be conducted in Greater Manchester, a region within the service area of the North West Ambulance Service (NWAS). NWAS is a National Health Service (NHS) trust provides emergency medical services and patient transportation across the North West region of England. It covers an extensive area of over 5,400 square miles, providing emergency and non-emergency medical services to a population of over 7.5 million people. There are two adults MTCs in Greater Manchester, which are part of the network covered by NWAS. These are Salford Royal Hospital and Manchester Royal Infirmary. In addition to these MTCs, the network also includes several trauma units and local emergency hospitals, each playing a distinct role in the trauma care pathway. The study protocol has been prospectively registered through the ISRCTN registry (study ID: ISRCTN92566288).

For the purpose of the study, we modified the CCHR for prehospital use (Table 1). The original rule excluded patients <16 years, patients with a bleeding disorder or users of oral anticoagulants and/or antiplatelet and or those experiencing a post-traumatic seizure. In the modified version, only patients under the age of 16 years will be excluded. However, patients

For peer review only

taking anticoagulant or antiplatelet drugs will be included and will be considered 'positive' with the modified CCHR (i.e., would require transport to hospital). Anticoagulant use is defined as the use of warfarin, edoxaban, dabigatran, apixaban, and rivaroxaban. Antiplatelets use is defined as the use of clopidogrel, ticlopidine, ticagrelor, dipyridamole and prasugrel. Figure 1 displays a visual representation of the recruitment process. At the time of writing in April 2024, the study is actively in the recruitment phase and open for participant enrolment.

Participants

Patient eligibility

Inclusion criteria

This study will be conducted using a convenience sample of adult patients with mild TBI who will be transported to Northern Care Alliance NHS Foundation Trust and Manchester University Hospital NHS Foundation Trust. Only those patients who meet the following criteria will be included:

- Adult patients (≥16 years) who receive an emergency ambulance response for a primary complaint of head injury. This includes those involved in Motor Vehicle Crashes (MVCs), provided they meet the other inclusion criteria.
- Glasgow Coma Scale (GCS) score of 13–15 at the time of assessment by attending paramedics.
- Patients transported to the hospital for clinical care.

Exclusion criteria

- Secondary transfers patients including interfacility transport.
- Penetrating skull injury.

For peer review only

- Trauma to other body regions that require clinical treatment, indicating multisystem trauma. This includes patients involved in MVCs where the treating paramedic assesses the presence of injuries beyond isolated head trauma that require clinical intervention. Paramedics will apply their clinical judgment to identify and exclude such cases.
- Prisoners.

Table1: The original and modified versions of the Canadian CT Head rule

**Canadian CT head rule (original)**	**Canadian CT head rule (modified)**
**High risk (for neurosurgical interventions)**	**High risk (for neurosurgical interventions)**
- GCS score <15 at 2 hrs after injury	- GCS score <15 at presentation
- Suspected open or depressed skull fracture	- Suspected open or depressed skull fracture
- Any sign of basal skull fracture	- Any sign of basal skull fracture
- Vomiting ≥ 2 episodes	- Vomiting ≥ 2 episodes
- Age ≥ 65 years	- Age ≥ 65 years
**Medium risk (for brain injury on CT)**	**Medium risk (for brain injury on CT)**
Amnesia before impact > 30 minutes	- Amnesia before impact > 30 minutes
Dangerous mechanism of injury (pedestrian struck by vehicle, ejection from vehicle, fall from >3 feet or >5 stairs.	Dangerous mechanism of injury (pedestrian struck by vehicle, ejection from vehicle, fall from >3 feet or >5 stairs.
* Patients with any positive high risk or medium risk criteria will be considered to have a 'positive' outcome (requiring transport to a hospital)	

Study duration and sample size

This is a feasibility study, in which evaluating recruitment rate is an outcome; therefore, it is not possible to specify a sample size a priori. However, we intend to recruit participants for a

For peer review only - http://bmjopen.bmj.com/site/about/guidelines.xhtml

fixed period of six months. Based on data routinely collected by the Manchester University NHS Foundation Trust, a total of 5,332 patients arrived in the ED by ambulance with a primary complaint of head injury over a period of four years and nine months up to November 2020. We intend to recruit participants from two similarly sized MTCs. We anticipate that approximately 1,100 patients will be eligible to participate in this study during the recruitment period. Accounting for the availability of trained paramedics on the study delegation log and concurrent injuries to other body systems that would exclude patients from participating in this study, approximately 100 participants can be recruited to this study. This number of participants is generally accepted as being appropriate for studies that focus on assessing feasibility and acceptability (20).

Outcomes

We will study the following feasibility outcomes:

- The number of eligible patients who are approached.
- The proportion of those approached who consent to participate in the study.
- The completeness of data collection.
- The completeness of follow-up.
- Determination of the inter-observer reliability of the CCHR completed by prehospital care providers and ED physicians.
- Determination of the inter-observer reliability for each component of the CCHR assessed by prehospital care providers and ED physicians.
- The diagnostic performance and clinical sensibility of the CCHR in the opinion of attending paramedics.
- Determine the acceptability of trial processes as perceived by paramedics and patients.

For peer review only

Additionally, as a preliminary evaluation of the diagnostic accuracy of the CCHR, the presence of traumatic intracranial lesions requiring neurosurgical intervention will be examined. This includes any neurosurgical intervention and/or abnormal head CT findings and/or death within seven days resulting from TBI.

Measuring and defining outcomes

Feasibility outcomes will be assessed using screening logs and case report forms completed by attending paramedics during the course of the study. At the end of the study period, a minimum of two clinicians (affiliated with the relevant NHS trusts) will review in-hospital patient records to diagnose the secondary outcomes of this feasibility study (neurosurgical significant TBI, abnormal CT findings and death within seven days). A third independent investigator will be invited to adjudicate any discrepancies. Diagnostic adjudication will be blinded to the final interpterion of the CCHR. Additionally, a neuroradiology consultant/expert will be consulted in case of the CT scan diagnosis is inconclusive.

Neurosurgical interventions are defined as any neurosurgical intervention including intracranial pressure monitoring or an Abbreviated Injury Scale (AIS) with the severity ≥3 in the head region. Abnormal head CT findings will be classified as normal or abnormal and are defined as at least one of the following: skull fractures, intracranial lesions, such as cerebral contusion and traumatic intracranial haemorrhages, or other clinically significant findings requiring hospital admission, neurosurgical interventions and routine follow-up.

For peer review only

Data collection

Prehospital setting

During the study period, all patients will receive standard prehospital care in accordance with local guidelines. Prior to their participation in the study, all participating paramedics will be provided with bespoke training specifically designed for the study. This training will cover all aspects of the study protocol, including eligibility assessment, the consent process, data collection procedures, and guidance on interpreting the CCHR. To ensure accessibility and flexibility, the training will be offered both face-to-face and through various online sessions conducted via Zoom. Additionally, the training sessions have been recorded and made available online for those unable to attend live. This approach allows paramedics to access the training material at their convenience, ensuring that all participants receive comprehensive training on the study protocols and data collection methods.

Prehospital data will be collected using Research Electronic Data Capture (REDCap), a secure web-based platform hosted at the University of Manchester. REDCap is a software application that was created to facilitate the process of collecting and managing research data. Prior to recruitment, a training session will be held to equip participating sites with data collection skills. In addition, paper case report forms (CRFs) will be available as an alternative method of data collection. It is expected that this approach will provide flexibility and ensure continuity in data collection. A variety of data will be collected in the prehospital setting, including, but not limited to:

- Baseline demographics information (i.e, gender - age)
- Anticoagulant use.
- Injury characteristics (i.e., mechanism of injury, place).

For peer review only

- Past medical history.
- Initial chief complaint (i.e., vomiting, headache, loss of consciousness, amnesia).
- Prehospital physiological parameters (i.e., GCS, vital signs).
- Clinical criteria required to use the CCHR.
- Destination hospital.

Paramedics will record these data contemporaneously, either prior to or immediately following handover at hospital.

In-hospital environment

REDCap will be utilised to collect hospital data, and a paper CRF will also be available as an alternative. Emergency physicians at the receiving hospital will assess the patients to determine whether a CT scan is necessary in accordance with local and national guidelines. Relevant data about imaging studies undertaken and their findings will be extracted from hospital records. The routine radiology reports will be used to extract the follow-up data required for this study. Where there is uncertainty about the interpretation of the radiology report, a second radiologist will be asked to interpret the images.

Further, emergency physicians at the receiving hospitals will be asked to assess the patients using the CCHR and record their findings, blinded to the assessments made by participating paramedics. These data will be used to determine the inter-observer reliability of the CCHR between paramedics in the prehospital environment and clinicians in the ED. Additionally, in-hospital medical records will be reviewed to obtain information regarding neurosurgical interventions undertaken during the patient's hospital admission.

For peer review only

Follow-up

Following the initial presentation to the ED, patients will be followed up to the time of hospital discharge. Data will be collected from the clinical records of the patient, including, but not limited to

- GCS on arrival in hospital.
- Details of the clinician's assessment of the CCHR.
- Outcome of any brain imaging (i.e., CT and/or MR scans, recorded as part of routine practice).
- AIS in relation to head injury.
- Injury Severity Score.
- Mortality status.
- Neurosurgical procedures and/or surgery.

Evaluation of acceptability

We will use a qualitative approach to evaluate the acceptability of trial processes used to study participants. We will purposively sample (a) study participants and (b) paramedics. The intention is to maximise heterogeneity (according to age, sex and ethnic origin, and for paramedics, seniority and base ambulance station). Patients and participating paramedics will be interviewed for the purpose of exploring their experiences of participating in a study of this nature. Previous emergency care research has demonstrated the value of this method in providing information regarding the feasibility and design of a larger study (21).

To maximise convenience for participants and thereby recruitment, participants will be invited to take part in a semi-structured interview either by telephone, face-to-face or video conference.

For peer review only

Informed consent will be obtained from all participants. We will explore the factors that facilitate or discourage participation in the research. Paramedics and study participants will be offered a £30 high street voucher to thank them for taking part. Telephone and sign language interpreters will be provided where necessary. The interview topic guides (Appendix 1) consist of a number of questions and prompts concerning the patients and paramedics' experiences of participating in this study, as well as the paramedics' perceptions of the viability of the CCHR. Prior to commencement of the interviews, two pilot interviews will be carried out. To determine the relevance, clarity and duration required to complete each interview, one will be with a paramedic and the other will be with a patient (22).

The Ottawa Acceptability of Decision Rules Instrument (OADRI) is a 12-item instrument that will be used to measure paramedics' attitudes toward the CCHR and clinical sensibility (23). Paramedics will be asked to provide their level of agreement with twelve statements about the CCHR using a six-point Likert scale. The first seven statements are worded in such a way that a higher number indicates higher acceptance, while the last five are worded in the opposite manner to minimise the acquiescence bias (24).

Consent process

An exception to the normal process of informed consent is established for patients in emergency situations who are unable to provide consent. As stated in the Declaration of Helsinki, "for a research subject who is legally incompetent, physically or mentally incapable of giving consent or is a legally incompetent minor, the investigator must obtain informed consent from the legally authorised representative in accordance with applicable law. These groups should not be included in research unless the research is necessary to promote the health

For peer review only

of the population represented and this research cannot instead be performed on legally competent persons" (25).

In this study, we will include patients in the prehospital environment who are receiving an emergency ambulance response having sustained a head injury. The nature of this presentation means that mental capacity to provide informed consent for participation in research will be compromised, especially given the time critical nature of the emergency medical response. Therefore, we will adopt a deferred consent model, which is widely used in research of this nature (26). Deferred consent in the emergency setting is a practice where medical treatment or research participation is initiated without prior consent from patients due to urgent circumstances and their compromised mental capacity, with the intention to obtain consent retrospectively once the patient's mental capacity has improved (27).

Paramedics will enrol eligible participants, enabling them to collect the required data. Patient care will be unchanged. We will then seek deferred written informed consent after the patients have received their initial treatment in hospital, and once they have regained their mental capacity.

When seeking written informed consent, a participant information sheet will be provided. Deferred consent may be obtained by either of the following means: (a) a member of staff may seek written informed consent at the receiving hospital; or (b) deferred consent may be sought in the community by post (written informed consent), email or telephone. While seeking deferred consent, staff will be available to answer any questions that the participants may have.

For participants lacking capacity, we will seek consent from their relative or legally authorised representative, which is known as proxy consent (28). A research nurse will briefly explain to

For peer review only - http://bmjopen.bmj.com/site/about/guidelines.xhtml

them that the patient will receive the usual hospital care for TBI. The responsible nurse will also clarify that only basic clinical data will be reported using a case report form to be analysed to improve prehospital care. A brief information sheet will be provided upon request. The ability of participants to provide consent can be assessed at any point during the study period. Participants who regain capacity will be provided with a detailed participant information sheet and their written consent to continue in this feasibility study will be obtained. If the patient does not regain capacity, we will seek deferred assent from a relative or professional legal representative to retain the patient's data in the study.

Data analysis

Quantitative data

A descriptive analysis will be conducted based on the baseline data for patients enrolled in the study to characterise the demographic and clinical profile. This will include the number of eligible participants who were approached, the proportion that consented to participate, data completeness (expressed as number and percentage) for each variable collected and number and percentage of participants completing follow-up. We will also report recruitment data as rates (number of participants enrolled per week across the study period).

The inter-observer reliability between paramedics and emergency physicians' use of the CCHR will be calculated using Cohen's Kappa, a statistical method for measuring agreement between two observers, together with 95% confidence intervals. Additionally, we will report upon the CCHR's diagnostic performance for ruling out clinically significant TBI. This will be achieved by calculating the sensitivity, specificity, positive and negative likelihood ratios with 95% confidence intervals against a reference standard of traumatic intracranial lesion, which will be

For peer review only

identified from in-hospital records. Given the nature of this feasibility study, the analyses will be viewed as exploratory, preliminary and will be descriptive in nature. Furthermore, to determine the acceptability of the CCHR, the OADRI average item scores will be calculated, with higher scores indicating greater acceptability.

Qualitative data

The six-stage thematic analysis approach will be employed to analyse all interviews, as suggested by Braun and Clarke (29). This involves becoming familiar with the data, generating initial codes, searching for themes, reviewing themes, defining and naming themes and sub-themes, and producing the report. We will adhere to the four criteria for trustworthiness established by Lincoln and Guba: credibility, dependability, confirmability and transferability (30). Each step of the qualitative data collection and analysis will be guided by a qualitative research expert. The qualitative data will be managed and analysed using NVivo 12.

Progression criteria

This feasibility study will be overseen and governed by the Trial Steering Committee (TSC), which will include the chief investigator, principal investigator, two lay members, topic experts in TBI and one paramedic. The role of the TSC is to provide external oversight and to ensure that the study is conducted in compliance with Good Clinical Practice guidelines (31, 32).

The traffic light approach (green/amber/red), also known as a (stop-amend-go) rule, will be used to determine whether or not to proceed with a full definitive trial (33). Green (go) indicates that the study meets the pre-set criteria, and that it would be feasible for a full-powered definitive trial to be conducted without any modifications to the study protocol. Amber (amend) indicates that further feasibility work or modifications to the protocol are required before proceeding with the full trial. Red (stop) indicates that proceeding to a definitive study is not

For peer review only

recommended. Taking into account the feasibility outcomes, as well as qualitative interviews with patients and paramedics, a decision will be made regarding whether or not to proceed with the trial or whether to make any amendments to the study design. The progression criteria are shown in Table 2.

Patient and public involvement

It has become increasingly apparent that involving patients and the public in healthcare research can enhance the development and implementation of clinical studies, the recruitment of participants, and the relevance of study topics and outcomes in relation to their needs and experiences (34, 35). It will be a priority to take into account the perspectives of patients and the public during the design, conduct, reporting and dissemination of the research. To ensure maximum clinical impact, the study has been discussed with patient and public representatives through the NIHR Brain Injury MedTech Co-operative. A comprehensive and in-depth discussion was conducted regarding the study procedures, including key aspects such as the consent process and patient-facing materials. Two members have agreed to join the study steering committee, which is expected to meet two or three times during the study period to advise on the progress of the research, discuss any adverse events, proposed protocol amendments and other matters relating to research governance. Additionally, a lay summary will be developed in collaboration with the lay members to disseminate the study findings.

For peer review only

Table 2: Progression criteria to proceed with a definitive trial.

	Contributing data	Green (Proceed to a definitive trial)	Amber (Proceed to a definitive trial following some changes to the protocol	Do not proceed to a definitive trial
**Screening**	Training and delegation logs	At least 20 paramedics at participating ambulance hubs trained and on delegation logs	At least 10 paramedics at participating ambulance hubs trained and on delegation logs	Less than 10 paramedics at participating ambulance hubs trained and on delegation logs
**Recruitment acceptability**	Screening & recruitment log	≥ 80% of eligible screened patients are consented to take part	≥ 60% of eligible screened patients are consented to take part	< 60% of eligible screened patients are consented to take part
**Retention**	Screening & recruitment log	Retention of ≥ 90% of study participants until study completion	Retention of ≥ 80% of study participants until study completion	Retention of < 80% of study participants until study completion
**Study procedures**	Participation data	At least 85% of the outcome data is collected (e.g., case report forms)	A minimum of 60% of the outcome data is collected (e.g., case report forms)	< 60% of the outcome data is collected (e.g., case report forms)
**Adverse events**	Participation data	No or very minor adverse events and no participants discontinued the study	Minor or serious adverse events leading to 10% or less participants discontinuing the study	Serious adverse events leading to >10% of participants discontinuing the study
**Acceptability**	Participant interview Paramedics questionnaire and interviews	Responses to OADRI* all 'agree' or 'strongly agree'. No major barriers to acceptability/participation identified during interviews with paramedics and patients	Responses to OADRI mainly 'agree' or 'strongly agree'. Some barriers to participation identified during interviews but remediable action identified.	Responses to OADRI mainly 'disagree' or 'strongly disagree'. Major barriers to acceptability/participation identified during interviews with no clear remediable action.

* OADRI: Ottawa Acceptability of Decision Rules Instrument

Safety considerations, adverse events and serious adverse events

Given the nature of this study, any risk to participants is very low. It is important to note that this study is observational in nature: a patient's participation in this study will have no effect

For peer review only

on the quality of medical care they usually receive in both prehospital and hospital settings. Our study will have no impact on the decision to undergo a CT scan as this will be undertaken at the discretion of the treating clinician, as part of routine clinical care, in accordance with current national guidance. The study will also have no effect on the decision to transport patients to the hospital. There are also small risks associated with data management, but we have carefully considered how data will be handled during the study and we will operate in accordance with current legislation and the standard operating procedures of the sponsor organisation to mitigate that risk. We are not aware of any potential risks this study would pose to the researchers.

Because of the low-risk nature of the study, we do not anticipate any adverse events (including serious adverse events) as a result of participating in this research. However, given the patient group (patients with TBI), we anticipate that many patients will experience expected adverse events, which are related to their initial injury. We will not ask sites to report expected adverse events. Expected serious adverse events include, for example, death due to head injury; neurosurgical procedures for TBI; admission to hospital for traumatic brain injury; loss of function due to TBI. Unexpected adverse events and serious adverse events should be reported. Adverse events will be defined in accordance with ICH Good Clinical Practice as any untoward medical occurrence in a participant, but will only be reported if this was not expected as a result of the patient's condition, up to the point of handover in hospital.

Impact

It is anticipated that the feasibility study will provide valuable insight into whether conducting a full trial in the future is warranted. It will also provide guidance to enhance the study design,

For peer review only

recruitment plan, and consent process. If feasibility is established, we will prepare to run a fully powered multi-centre study to evaluate the diagnostic accuracy of using the CCHR in the prehospital field. This will also involve drafting a proposal to apply for research funding.

The outcomes relating to diagnostic accuracy will be of particular relevance given our knowledge that this is the first study investigating the feasibility of using the CCHR in the prehospital environment. Therefore, it is anticipated that the findings of this feasibility study will be used to inform design of a fully powered diagnostic test accuracy study. The findings of this study will be complemented by the findings of mixed methods research that we are also conducting which will explore facilitators and barriers to implementing new diagnostic pathways in the prehospital environment as perceived by practising paramedics (36).

In parallel with a fully powered diagnostic test accuracy study, it will also be important to work with key stakeholders including primary care practitioners, neurosurgeons and patient and public representatives. The care pathway for patients who are not conveyed to hospital will need to be defined to ensure that patients are adequately supported with appropriate safety netting for those who experience ongoing symptoms or complications.

Ethics and dissemination

Ethical approval to conduct this feasibility study has been obtained from the Health Research Authority and the Research Ethics Committee (REC reference: 22/NW/0358). Any substantial amendment to the study protocol will need to be approved by an ethics committee after being approved by the sponsor. To ensure privacy and confidentiality, all records and files relating to the study will be kept in a secure location and will only be accessible to members of the

For peer review only

research team. The results will be published in peer-reviewed journals, presented at relevant national and international conferences, and will be incorporated into a doctoral thesis (NA). The study findings will also be disseminated through presentations at relevant academic forums.

Declarations

Ethics approval and consent to participate

The nature of this study requires ethical approval from the Health Research Authority and Research Ethics Committee, which has been obtained (REC reference: 22/NW/0358).

Consent for publication

Not applicable.

Availability of data and material

Not applicable.

Competing interests

Richard Body has consulted for Abbott Point of Care and is undertaking contract clinical research with BrainBox, which manufactures assays for biomarkers of TBI.

Funding

This work is supported by a PhD scholarship awarded to NA by the Saudi Arabian Cultural Bureau in the UK and King Saud University (KSU1872). The funders had no role in the design of this study protocol. The study is sponsored by the University of Manchester. The publication fee is funded by the cardiovascular department, University of Manchester, UK. No grant or award number.

For peer review only

Authors contributions

NA collaborated with the study team (SB, SC, FL, and RB) in preparing the initial study protocol. The protocol was developed through collective input and discussions. NA took the lead in drafting the manuscript, working closely with SB, SC, FL, and RB to ensure its accuracy and completeness. The final version of the manuscript was approved by all authors.

Acknowledgements

Not applicable.

Author affiliations

[1]Division of Cardiovascular Sciences, University of Manchester, Manchester, UK. [2]Department of Accidents and Trauma, Prince Sultan bin Abdulaziz College for Emergency Medical Services, King Saud University, Riyadh, Saudi Arabia. [3]Medical Directorate, North West Ambulance Service NHS Trust, Bolton, BL1 5DD, UK. [4] Emergency Department, Manchester University NHS Foundation Trust, Manchester, UK. [5]University of Sheffield, School of Health and Related Research, Sheffield, UK.

For peer review only

References

1. Berkner J, Mannix R, Qiu J. Clinical Traumatic Brain Injury in the Preclinical Setting. Methods Mol Biol. 2016;1462:11-28.
2. Majdan M, Plancikova D, Brazinova A, Rusnak M, Nieboer D, Feigin V, et al. Epidemiology of traumatic brain injuries in Europe: a cross-sectional analysis. Lancet Public Health. 2016;1(2):e76-e83.
3. Anandalwar SP, Mau CY, Gordhan CG, Majmundar N, Meleis A, Prestigiacomo CJ, et al. Eliminating unnecessary routine head CT scanning in neurologically intact mild traumatic brain injury patients: implementation and evaluation of a new protocol. J Neurosurg. 2016;125(3):667-73.
4. Decuypere M, Klimo P, Jr. Spectrum of traumatic brain injury from mild to severe. Surg Clin North Am. 2012;92(4):939-57, ix.
5. Haydel MJ, Preston CA, Mills TJ, Luber S, Blaudeau E, DeBlieux PM. Indications for computed tomography in patients with minor head injury. N Engl J Med. 2000;343(2):100-5.
6. Stiell IG, Lesiuk H, Wells GA, McKnight RD, Brison R, Clement C, et al. The Canadian CT Head Rule Study for patients with minor head injury: rationale, objectives, and methodology for phase I (derivation). Ann Emerg Med. 2001;38(2):160-9.
7. Stiell IG, Lesiuk H, Wells GA, Coyle D, McKnight RD, Brison R, et al. Canadian CT head rule study for patients with minor head injury: methodology for phase II (validation and economic analysis). Ann Emerg Med. 2001;38(3):317-22.
8. Stiell IG, Clement CM, Rowe BH, Schull MJ, Brison R, Cass D, et al. Comparison of the Canadian CT Head Rule and the New Orleans Criteria in patients with minor head injury. Jama. 2005;294(12):1511-8.
9. Papa L, Stiell IG, Clement CM, Pawlowicz A, Wolfram A, Braga C, et al. Performance of the Canadian CT Head Rule and the New Orleans Criteria for predicting any traumatic intracranial injury on computed tomography in a United States Level I trauma center. Acad Emerg Med. 2012;19(1):2-10.
10. Bouida W, Marghli S, Souissi S, Ksibi H, Methammem M, Haguiga H, et al. Prediction value of the Canadian CT head rule and the New Orleans criteria for positive head CT scan and acute neurosurgical procedures in minor head trauma: a multicenter external validation study. Ann Emerg Med. 2013;61(5):521-7.
11. Bonney PA, Briggs A, Briggs RG, Jarvis CA, Attenello F, Giannotta SL. Rate of Intracranial Hemorrhage After Minor Head Injury. Cureus. 2020;12(9):e10653.
12. National Clinical Guideline C. National Institute for Health and Clinical Excellence: Guidance. Head Injury: Triage, Assessment, Investigation and Early Management of Head Injury in Children, Young People and Adults. London: National Institute for Health and Care Excellence (UK)
Copyright © National Clinical Guideline Centre, 2023.; 2023.
13. Pandor A, Goodacre S, Harnan S, Holmes M, Pickering A, Fitzgerald P, et al. Diagnostic management strategies for adults and children with minor head injury: a systematic review and an economic evaluation. Health Technol Assess. 2011;15(27):1-202.
14. Sultan HY, Boyle A, Pereira M, Antoun N, Maimaris C. Application of the Canadian CT head rules in managing minor head injuries in a UK emergency department: implications for the implementation of the NICE guidelines. Emergency Medicine Journal. 2004;21(4):420.

For peer review only

15. Hassan Z, Smith M, Littlewood S, Bouamra O, Hughes D, Biggin C, et al. Head injuries: a study evaluating the impact of the NICE head injury guidelines. Emergency medicine journal. 2005;22(12):845-9.
16. Lawrence T, Helmy A, Bouamra O, Woodford M, Lecky F, Hutchinson PJ. Traumatic brain injury in England and Wales: prospective audit of epidemiology, complications and standardised mortality. BMJ Open. 2016;6(11):e012197.
17. O'Cathain A, Knowles E, Bishop-Edwards L, Coster J, Crum A, Jacques R, et al. Understanding variation in ambulance service non-conveyance rates: a mixed methods study. Health Services and Delivery Research. 2018.
18. Mason S, Kuczawski M, Teare MD, Stevenson M, Goodacre S, Ramlakhan S, et al. AHEAD study: an observational study of the management of anticoagulated patients who suffer head injury. BMJ open. 2017;7(1):e014324.
19. Arain M, Campbell MJ, Cooper CL, Lancaster GA. What is a pilot or feasibility study? A review of current practice and editorial policy. BMC medical research methodology. 2010;10(1):1-7.
20. Teresi JA, Yu X, Stewart AL, Hays RD. Guidelines for Designing and Evaluating Feasibility Pilot Studies. Med Care. 2022;60(1):95-103.
21. van den Berg P, Kendal S, Alderson HV, Body R. An exploration of patients' experiences of participation in a randomised controlled trial of the Manchester Acute Coronary Syndromes (MACS) decision rule. Emerg Med J. 2017;34(9):593-8.
22. Majid MAA, Othman M, Mohamad SF, Lim SAH, Yusof A. Piloting for interviews in qualitative research: Operationalization and lessons learnt. International Journal of Academic Research in Business and Social Sciences. 2017;7(4):1073-80.
23. Brehaut JC, Graham ID, Wood TJ, Taljaard M, Eagles D, Lott A, et al. Measuring acceptability of clinical decision rules: validation of the Ottawa acceptability of decision rules instrument (OADRI) in four countries. Medical Decision Making. 2010;30(3):398-408.
24. Schriesheim C, Hill K. Controlling Acquiescence Response Bias by Item Reversals: The Effect on Questionnaire Validity. Educational and Psychological Measurement - EDUC PSYCHOL MEAS. 1981;41:1101-14.
25. Association WM. World Medical Association Declaration of Helsinki: ethical principles for medical research involving human subjects. Jama. 2013;310(20):2191-4.
26. Kompanje E, Jansen T, Le Noble J, de Geus H, Bakker J. Deferred consent for inclusion of patients unable to give their consent in studies in the field of emergency medicine. Nederlands Tijdschrift Voor Geneeskunde. 2008;152(38):2057-61.
27. van der Graaf R, Hoogerwerf MA, de Vries MC. The ethics of deferred consent in times of pandemics. Nat Med. 2020;26(9):1328-30.
28. Kompanje EJO, Maas AIR, Hilhorst M, Slieker F, Teasdale G. Ethical considerations on consent procedures for emergency research in severe and moderate traumatic brain injury. Acta Neurochirurgica. 2005;147:633-40.
29. Braun V, Clarke V. Using thematic analysis in psychology. Qualitative research in psychology. 2006;3(2):77-101.
30. Forero R, Nahidi S, De Costa J, Mohsin M, Fitzgerald G, Gibson N, et al. Application of four-dimension criteria to assess rigour of qualitative research in emergency medicine. BMC Health Serv Res. 2018;18(1):120.
31. Daykin A, Selman L, Cramer H, McCann S, Shorter G, Sydes M, et al. What are the roles and valued attributes of a Trial Steering Committee? Ethnographic study of eight clinical trials facing challenges. Trials. 2016;17:307.

For peer review only

32. Harman NL, Conroy EJ, Lewis SC, Murray G, Norrie J, Sydes MR, et al. Exploring the role and function of trial steering committees: results of an expert panel meeting. Trials. 2015;16:597.
33. Avery KNL, Williamson PR, Gamble C, Connell Francischetto E, Metcalfe C, Davidson P, et al. Informing efficient randomised controlled trials: exploration of challenges in developing progression criteria for internal pilot studies. BMJ Open. 2017;7(2):e013537.
34. Price A, Albarqouni L, Kirkpatrick J, Clarke M, Liew SM, Roberts N, et al. Patient and public involvement in the design of clinical trials: an overview of systematic reviews. Journal of evaluation in clinical practice. 2018;24(1):240-53.
35. Gamble C, Dudley L, Allam A, Bell P, Goodare H, Hanley B, et al. Patient and public involvement in the early stages of clinical trial development: A systematic cohort investigation. BMJ open. 2014;4:e005234.
36. Alqurashi N, Alotaibi A, Bell S, Lecky F, Body R. Towards exploring current challenges and future opportunities relating to the prehospital triage of patients with traumatic brain injury: a mixed-methods study protocol. BMJ Open. 2023;13(3):e068555.

Figure legends:

- **Figure 1: Study flowchart**

For peer review only

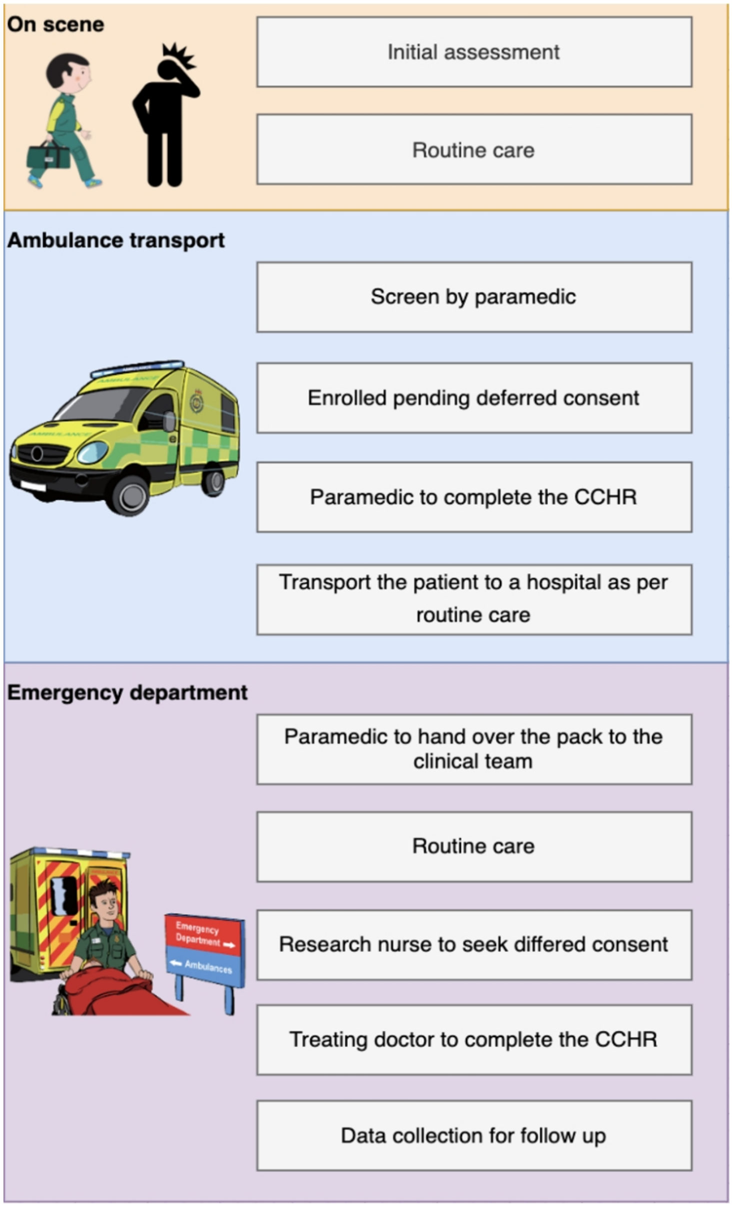

Figure 1: Study flowchart

149x247mm (144 x 144 DPI)

Head injury Evaluation and Ambulance Diagnosis: A Feasibility Study

Semi-structured interview, topic guide – Patients

Date: **Participant ID number:**

Pre-interview

- Privacy notice read.
- Consent form.
- Information sheet.

---

1. Please share your experience from the moment you were initially approached about the study.
   - **Follow-up questions, if required:**

   a. How did you feel about being approached for the study? Were there any specific factors that influenced your decision to participate?
   b. Were you given enough information to help you to decide whether to take part in this study? Was there anything that you found confusing or unclear?
   c. In this study, we didn't ask patients for permission during the ambulance journey. This is because they had experienced a head injury and were being taken to hospital as an emergency. How did you feel about this? Do you think you would have been able to make a decision about taking part when you were still in the ambulance?

2. How did you feel about the way we asked for your permission to take part in this study?
   - **Follow-up questions, if required:**

   a. Please share your overall thoughts about how we asked for your permission to take part in this study.
   b. Is there anything particularly good or bad about the way we asked for your permission to take part in the study?
   c. Do you think you were given enough time to decide whether to take part? Why or why not?

3. Can you recall any specific reasons or factors that influenced your belief that it is important to understand the study? How did this understanding affect your decision to take part in this study?

For peer review only - http://bmjopen.bmj.com/site/about/guidelines.xhtml

4. In your opinion, are there any factors that you believe might discourage people from participating in this study?

Follow-up questions, if required:

a. Is there anything that might discourage people from participating in this study? How do you think these factors could be addressed?

5. Can you share any unexpected events or experiences that occurred during your participation in the study?

6. Would you recommend taking part in this trial to somebody else?

Follow-up questions, if required:

a. Can you explain your thoughts and the reasons behind your recommendation or non-recommendation of participation in this study to others?
b. In your opinion, what specific factors or aspects of the study influenced your recommendation or non-recommendation?
c. Is there anything that you think would stop people from fully participating in this study?

7. Is there anything else you would still like to know about this study?

******END******

For peer review only - http://bmjopen.bmj.com/site/about/guidelines.xhtml

Head injury Evaluation and Ambulance Diagnosis: A Feasibility Study

Semi-structured interview, topic guide - Paramedics

Date: **Participant ID number:**

Pre-interview

- Privacy notice read.
- Consent form.
- Information sheet.

---

1. Firstly, I would like to know how long you have been a paramedic, and what is your highest academic qualification level?

2. Have you completed the training on the study protocol?
   a. How did you find it?
   b. Did you find the study protocol easy to understand?
   c. Was the protocol easy to remember in practice?
   d. What could be improved in the trial protocol?
3. What was the best aspect of being involved in the study?
   a. Could you tell me how easy it was to use the study case report form?

4. What would be the advantages of using the Canadian CT head Rule in the prehospital setting?
   a. Looking back to your experience, could you tell me how did you find using the Canadian CT head rule in the prehospital field?

5. What would be the disadvantages of using the Canadian CT head Rule in the prehospital setting?
   a. Looking back to your experience, did you experience any barriers/challenges to applying the Canadian CT Head Rule?

6. Would you like the Canadian CT Head Rule to become part of your practice?

7. Do you have any additional thoughts you would like to share about the study?

END

For peer review only - http://bmjopen.bmj.com/site/about/guidelines.xhtml

SPIRIT Checklist

		Reporting Item	Page Number
**Administrative information**			
Title	#1	Descriptive title identifying the study design, population, interventions, and, if applicable, trial acronym	1
Trial registration	#2a	Trial identifier and registry name. If not yet registered, name of intended registry	8
Trial registration: data set	#2b	All items from the World Health Organization Trial Registration Data Set	NA
Protocol version	#3	Date and version identifier	NA
Funding	#4	Sources and types of financial, material, and other support	24
Roles and responsibilities: contributorship	#5a	Names, affiliations, and roles of protocol contributors	24,25
Roles and responsibilities: sponsor contact information	#5b	Name and contact information for the trial sponsor	24
Roles and responsibilities: sponsor and funder	#5c	Role of study sponsor and funders, if any, in study design; collection, management, analysis, and interpretation of data; writing of the report; and the decision to submit the report for publication, including whether they will have ultimate authority over any of these activities	24
Roles and responsibilities: committees	#5d	Composition, roles, and responsibilities of the coordinating centre, steering committee, endpoint adjudication committee, data management team, and other individuals or groups overseeing the trial, if applicable (see Item 21a for data monitoring committee)	19
**Introduction**			

For peer review only

Background and rationale	#6a	Description of research question and justification for undertaking the trial, including summary of relevant studies (published and unpublished) examining benefits and harms for each intervention	5-7
Background and rationale: choice of comparators	#6b	Explanation for choice of comparators	NA
Objectives	#7	Specific objectives or hypotheses	8
Trial design	#8	Description of trial design including type of trial (eg, parallel group, crossover, factorial, single group), allocation ratio, and framework (eg, superiority, equivalence, non-inferiority, exploratory)	8,9
**Methods: Participants, interventions, and outcomes**			
Study setting	#9	Description of study settings (eg, community clinic, academic hospital) and list of countries where data will be collected. Reference to where list of study sites can be obtained	8,9
Eligibility criteria	#10	Inclusion and exclusion criteria for participants. If applicable, eligibility criteria for study centres and individuals who will perform the interventions (eg, surgeons, psychotherapists)	9,10
Interventions: description	#11a	Interventions for each group with sufficient detail to allow replication, including how and when they will be administered	NA
Interventions: modifications	#11b	Criteria for discontinuing or modifying allocated interventions for a given trial participant (eg, drug dose change in response to harms, participant request, or improving / worsening disease)	NA
Interventions: adherance	#11c	Strategies to improve adherence to intervention protocols, and any procedures for monitoring adherence (eg, drug tablet return; laboratory tests)	13-15
Interventions: concomitant care	#11d	Relevant concomitant care and interventions that are permitted or prohibited during the trial	NA
Outcomes	#12	Primary, secondary, and other outcomes, including the specific measurement variable (eg, systolic blood pressure), analysis	11,12

For peer review only

		metric (eg, change from baseline, final value, time to event), method of aggregation (eg, median, proportion), and time point for each outcome. Explanation of the clinical relevance of chosen efficacy and harm outcomes is strongly recommended	
Participant timeline	#13	Time schedule of enrolment, interventions (including any run-ins and washouts), assessments, and visits for participants. A schematic diagram is highly recommended (see Figure)	13-15 and Figure 1
Sample size	#14	Estimated number of participants needed to achieve study objectives and how it was determined, including clinical and statistical assumptions supporting any sample size calculations	11
Recruitment	#15	Strategies for achieving adequate participant enrolment to reach target sample size	11
**Methods: Assignment of interventions (for controlled trials)**			
Allocation: sequence generation	#16a	Method of generating the allocation sequence (eg, computer-generated random numbers), and list of any factors for stratification. To reduce predictability of a random sequence, details of any planned restriction (eg, blocking) should be provided in a separate document that is unavailable to those who enrol participants or assign interventions	NA
Allocation concealment mechanism	#16b	Mechanism of implementing the allocation sequence (eg, central telephone; sequentially numbered, opaque, sealed envelopes), describing any steps to conceal the sequence until interventions are assigned	NA
Allocation: implementation	#16c	Who will generate the allocation sequence, who will enrol participants, and who will assign participants to interventions	NA
Blinding (masking)	#17a	Who will be blinded after assignment to interventions (eg, trial participants, care providers, outcome assessors, data analysts), and how	NA
Blinding (masking): emergency unblinding	#17b	If blinded, circumstances under which unblinding is permissible, and procedure for revealing a participant's allocated intervention during the trial	NA
**Methods: Data**			

For peer review only

**collection, management, and analysis**			
Data collection plan	#18a	Plans for assessment and collection of outcome, baseline, and other trial data, including any related processes to promote data quality (eg, duplicate measurements, training of assessors) and a description of study instruments (eg, questionnaires, laboratory tests) along with their reliability and validity, if known. Reference to where data collection forms can be found, if not in the protocol	13-15
Data collection plan: retention	#18b	Plans to promote participant retention and complete follow-up, including list of any outcome data to be collected for participants who discontinue or deviate from intervention protocols	15
Data management	#19	Plans for data entry, coding, security, and storage, including any related processes to promote data quality (eg, double data entry; range checks for data values). Reference to where details of data management procedures can be found, if not in the protocol	13,14
Statistics: outcomes	#20a	Statistical methods for analysing primary and secondary outcomes. Reference to where other details of the statistical analysis plan can be found, if not in the protocol	18,19
Statistics: additional analyses	#20b	Methods for any additional analyses (eg, subgroup and adjusted analyses)	NA
Statistics: analysis population and missing data	#20c	Definition of analysis population relating to protocol non-adherence (eg, as randomised analysis), and any statistical methods to handle missing data (eg, multiple imputation)	18,19
**Methods: Monitoring**			
Data monitoring: formal committee	#21a	Composition of data monitoring committee (DMC); summary of its role and reporting structure; statement of whether it is independent from the sponsor and competing interests; and reference to where further details about its charter can be found, if not in the protocol. Alternatively, an explanation of why a DMC is not needed	19,20
Data monitoring: interim analysis	#21b	Description of any interim analyses and stopping guidelines, including who will have access to these interim results and make the final decision to terminate the trial	19,20

For peer review only

Harms	#22	Plans for collecting, assessing, reporting, and managing solicited and spontaneously reported adverse events and other unintended effects of trial interventions or trial conduct	21,22
Auditing	#23	Frequency and procedures for auditing trial conduct, if any, and whether the process will be independent from investigators and the sponsor	19
**Ethics and dissemination**			
Research ethics approval	#24	Plans for seeking research ethics committee / institutional review board (REC / IRB) approval	23
Protocol amendments	#25	Plans for communicating important protocol modifications (eg, changes to eligibility criteria, outcomes, analyses) to relevant parties (eg, investigators, REC / IRBs, trial participants, trial registries, journals, regulators)	23
Consent or assent	#26a	Who will obtain informed consent or assent from potential trial participants or authorised surrogates, and how (see Item 32)	16-18
Consent or assent: ancillary studies	#26b	Additional consent provisions for collection and use of participant data and biological specimens in ancillary studies, if applicable	NA
Confidentiality	#27	How personal information about potential and enrolled participants will be collected, shared, and maintained in order to protect confidentiality before, during, and after the trial	23,24
Declaration of interests	#28	Financial and other competing interests for principal investigators for the overall trial and each study site	24
Data access	#29	Statement of who will have access to the final trial dataset, and disclosure of contractual agreements that limit such access for investigators	24
Ancillary and post trial care	#30	Provisions, if any, for ancillary and post-trial care, and for compensation to those who suffer harm from trial participation	NA
Dissemination policy: trial results	#31a	Plans for investigators and sponsor to communicate trial results to participants, healthcare professionals, the public, and other relevant groups (eg, via publication, reporting in results databases, or other data sharing arrangements), including any publication restrictions	23,24

For peer review only

Dissemination policy: authorship	#31b	Authorship eligibility guidelines and any intended use of professional writers	NA
Dissemination policy: reproducible research	#31c	Plans, if any, for granting public access to the full protocol, participant-level dataset, and statistical code	NA
**Appendices**			
Informed consent materials	#32	Model consent form and other related documentation given to participants and authorised surrogates	
Biological specimens	#33	Plans for collection, laboratory evaluation, and storage of biological specimens for genetic or molecular analysis in the current trial and for future use in ancillary studies, if applicable	NA

For peer review only

Head Injury Evaluation and Ambulance Diagnosis (HOME) Study Protocol: A Feasibility Study Assessing the Implementation of the Canadian Computed Tomography Head Rule in the Prehospital Setting

Journal:	*BMJ Open*
Manuscript ID	bmjopen-2023-077191.R4
Article Type:	Protocol
Date Submitted by the Author:	15-May-2024

Complete List of Authors:	Alqurashi, Naif; The University of Manchester, Division of Cardiovascular Sciences; King Saud University, Department of Accidents and Trauma, Prince Sultan bin Abdelaziz College for Emergency Medical Services, King Saud University, Riyadh, Saudi Arabia.<br>Bell, Steve; North West Ambulance Service NHS Trust, Medical Directorate<br>Carley, Simon D; Manchester University NHS Foundation Trust, Emergency Department<br>Lecky, Fiona; University of Sheffield, School of Health and Related Research<br>Body, Richard; The University of Manchester, Division of Cardiovascular Sciences

**Primary Subject Heading**:	Emergency medicine
Secondary Subject Heading:	Evidence based practice
Keywords:	ACCIDENT & EMERGENCY MEDICINE, Feasibility Studies, TRAUMA MANAGEMENT

Head Injury Evaluation and Ambulance Diagnosis (HOME) Study Protocol: A Feasibility Study Assessing the Implementation of the Canadian Computed Tomography Head Rule in the Prehospital Setting

Naif Alqurashi (1,2) Steve Bell (3), Simon D Carley (4), Fiona Lecky (5), Richard Body (1,4)

1. Division of Cardiovascular Sciences, University of Manchester, Manchester, UK.
2. Department of Accidents and Trauma, Prince Sultan bin Abdulaziz College for Emergency Medical Services, King Saud University, Riyadh, Saudi Arabia.
3. Medical Directorate, North West Ambulance Service NHS Trust, Bolton, BL1 5DD, UK.
4. Emergency Department, Manchester University NHS Foundation Trust, Manchester, UK.
5. University of Sheffield, School of Health and Related Research, Sheffield, UK.

***Corresponding author:** Naif Alqurashi, Division of Cardiovascular Sciences, University of Manchester, 46 Grafton Street, Manchester, M13 9PL, United Kingdom; E-mail: nalqurashi1@ksu.edu.sa

Key words: Prehospital; Traumatic brain injury; Triage; Trauma centre; Feasibility study

Word count: 4495 words, excluding the abstract, strengths and limitations, tables and the declarations.

For peer review only

Abstract

Introduction

Traumatic brain injury (TBI) is a common presentation in the prehospital environment. At present, paramedics do not routinely use tools to identify low-risk patients who could be left at scene or taken to a local hospital rather than a major trauma centre. The Canadian Computed Tomography Head Rule (CCHR) was developed to guide the use of computed tomography imaging in hospital. It has not been evaluated in the prehospital setting. We aim to address this gap by evaluating the feasibility and acceptability of implementing the CCHR to patients and paramedics, and the feasibility of conducting a full-scale clinical trial of its use.

Methods and analysis

We will recruit adult patients who are being transported to an emergency department (ED) by ambulance after suffering a mild TBI. Paramedics will prospectively collect data for the CCHR. All patients will be transported to the ED, where deferred consent will be taken and the treating clinician will re-assess the CCHR, blinded to paramedic interpretation. The primary clinical outcome will be neurosurgically significant TBI. Feasibility outcomes include recruitment and attrition rates. We will assess acceptability of the CCHR to paramedics using the Ottawa Acceptability of Decision Rules Instrument. Inter-observer reliability of the CCHR will be assessed between paramedics and the treating clinician in the ED. Participating paramedics and patients will be invited to participate in semi-structured interviews to explore the acceptability of trial processes and facilitators and barriers to the use of the CCHR in practice. Data will be analysed thematically. We anticipate recruiting approximately 100 patients over six months.

For peer review only

Ethics and dissemination

This study was approved by the Health Research Authority and the Research Ethics Committee (REC reference: 22/NW/0358). The results will be published in a peer-reviewed journal, presented at conferences, and will be incorporated into a doctoral thesis.

For peer review only

Strengths and limitations of this study

- To our knowledge, this study will be the first to evaluate the feasibility and acceptability of implementing the Canadian CT Head Rule (CCHR) in the prehospital setting, filling an important gap in the existing literature.
- The study has a limited sample size, as its primary objective is to assess the acceptability and feasibility of implementing the CCHR in the prehospital setting.
- The findings of this study will be used in the future to inform the design of a fully powered diagnostic test accuracy study.

For peer review only

Introduction

Traumatic brain injury (TBI) is widely recognised as being one of the most common causes of death and disability especially among young adults (1); however, it is becoming more frequent in the older population (2). TBI is a heterogeneous pathophysiological condition that varies widely in terms of its aetiology, severity, clinical presentation, and outcomes. This broad spectrum of severity creates clinical challenges, such as the inappropriate allocation of major trauma centre (MTC) resources to less serious cases (3).

The goal of prehospital trauma triage tools is to efficiently and promptly direct patients to the medical facility best suited to their needs and the available resources. Most TBIs are mild in nature and can be treated at a local trauma unit or non-trauma centres (4). There is no evidence indicating that these patients should be transported to a specialist neurological centre. Therefore, accurate prehospital triage of these patients will reduce the likelihood of MTCs being overwhelmed with patients suffering from mild TBI or non-TBI injuries as well as reducing the costs that might result from over-triaging patients who are unlikely to benefit from the costly resources of an MTC.

The cranial CT scan is considered the gold standard for the emergency evaluation of patients with suspected intracranial injury (5). Various validated clinical decision tools, including the Canadian CT Head Rule (CCHR), have been developed through extensive prospective studies to assess the need for a CT scan after a minor head injury (6, 7). Comparative studies have examined various clinical tools, most notably the New Orleans Criteria (NOC) and the CCHR, revealing that although both demonstrate similar sensitivity, the CCHR generally provides greater specificity (8-10). Several studies have shown that the CCHR achieves 100% sensitivity

For peer review only

in identifying neurosurgical needs and captures significant CT findings, offering greater specificity (36%-65%) compared to other decision rules (9-11).

The National Institute for Health and Care Excellence (NICE) has guidelines that largely follow the CCHR to determine when to perform CT scans in cases of head injury (12). This approach is supported by evidence showing that the CCHR rule is the most cost-effective among various decision rules evaluated (13). Although the number of CT scans has increased, the admission rate has been halved (from 9% to 4%), resulting in a saving of £3,381 per 100 patients (14, 15). The implementation of NICE head injury guidelines indicates that the higher costs of CT scans, averaging about £100 per scan, are balanced by the savings from reduced hospital admissions, estimated to be around £847 per patient (13).

Considering that paramedics have access to all the necessary data for the CCHR, it might be feasible to utilise the CCHR in the prehospital setting to promote consistency in care and optimise resource utilisation. Prehospital implementation of the CCHR could enable paramedics to transport patients to lower-level or non-trauma centres, or even (if shown to be sufficiently sensitive) safely leave patients at the scene. However, no studies have explored whether clinical decision aids can be used to rule out neurosurgically significant TBI in patients with apparently mild TBI in the prehospital field.

In England and Wales, around one million emergency department (ED) visits annually are for head injuries, with 90% being minor (GCS 13-15), and the majority of patients are transported by ambulance services (16). Optimising healthcare resource use and enhancing patient care experiences can be greatly advanced by reducing unnecessary ambulance transports to distant MTCs, when appropriate and feasible (17, 18). Integrating the CCHR into prehospital field presents a valuable opportunity to improve the triage process, allowing prehospital care

For peer review only

providers to make informed decisions regarding the necessity of hospital transport and the level of care required, thereby optimising healthcare resources and potentially reducing healthcare costs. This approach also opens the door to exploring alternative care pathways for such patients, ensuring they receive appropriate and timely care without overwhelming hospital resources.

To obtain reliable estimates of the diagnostic accuracy of the CCHR, a large, multi-centre study is warranted. This would be expensive and time consuming; however, the findings of such a study could inform changes to clinical practice. Successful delivery of such research would depend heavily upon adherence to the study protocol and the rates of recruitment attrition. Before embarking on such a study, it is necessary to evaluate the viability of the intended study processes (19).

The purpose of this study is to establish the feasibility of a fully powered diagnostic-test accuracy study to evaluate the accuracy of the CCHR when applied by paramedics to patients with mild TBI in the prehospital setting.

Research question

Is it feasible to conduct a prospective clinical study to evaluate the diagnostic accuracy of the CCHR in the prehospital field to assist with making informed decisions regarding the appropriate level of care for patients with minor head injuries?

Objectives

- To determine the feasibility of a study to evaluate the diagnostic accuracy of the CCHR in the prehospital environment.

For peer review only

- To evaluate the inter-observer reliability of the CCHR between prehospital clinicians and ED physicians.
- To evaluate the clinical sensibility of using the CCHR in the prehospital environment and to measure paramedics' attitudes toward the CCHR.
- To obtain an estimate of the diagnostic accuracy of the CCHR when used in the prehospital setting.

Methods

Study design and setting

A prospective, multi-centre feasibility study will be conducted in Greater Manchester, a region within the service area of the North West Ambulance Service (NWAS). NWAS is a National Health Service (NHS) trust provides emergency medical services and patient transportation across the North West region of England. It covers an extensive area of over 5,400 square miles, providing emergency and non-emergency medical services to a population of over 7.5 million people. There are two adults MTCs in Greater Manchester, which are part of the network covered by NWAS. These are Salford Royal Hospital and Manchester Royal Infirmary. In addition to these MTCs, the network also includes several trauma units and local emergency hospitals, each playing a distinct role in the trauma care pathway. The study protocol has been prospectively registered through the ISRCTN registry (study ID: ISRCTN92566288).

For the purpose of the study, we modified the CCHR for prehospital use (Table 1). The original rule excluded patients <16 years, patients with a bleeding disorder or users of oral anticoagulants and/or antiplatelet and or those experiencing a post-traumatic seizure. In the modified version, only patients under the age of 16 years will be excluded. However, patients

For peer review only

taking anticoagulant or antiplatelet drugs will be included and will be considered 'positive' with the modified CCHR (i.e., would require transport to hospital). Anticoagulant use is defined as the use of warfarin, edoxaban, dabigatran, apixaban, and rivaroxaban. Antiplatelets use is defined as the use of clopidogrel, ticlopidine, ticagrelor, dipyridamole and prasugrel. Figure 1 displays a visual representation of the recruitment process. At the time of writing in April 2024, the study is actively in the recruitment phase and open for participant enrolment.

Participants

Patient eligibility

Inclusion criteria

This study will be conducted using a convenience sample of adult patients with mild TBI who will be transported to Northern Care Alliance NHS Foundation Trust and Manchester University Hospital NHS Foundation Trust. Only those patients who meet the following criteria will be included:

- Adult patients (≥16 years) who receive an emergency ambulance response for a primary complaint of head injury. This includes those involved in Motor Vehicle Crashes (MVCs), provided they meet the other inclusion criteria.
- Glasgow Coma Scale (GCS) score of 13–15 at the time of assessment by attending paramedics.
- Patients transported to the hospital for clinical care.

Exclusion criteria

- Secondary transfers patients including interfacility transport.
- Penetrating skull injury.

For peer review only

- Trauma to other body regions that require clinical treatment, indicating multisystem trauma. This includes patients involved in MVCs where the treating paramedic assesses the presence of injuries beyond isolated head trauma that require clinical intervention. Paramedics will apply their clinical judgment to identify and exclude such cases.
- Prisoners.

Table1: The original and modified versions of the Canadian CT Head rule

**Canadian CT head rule (original)**	**Canadian CT head rule (modified)**
**High risk (for neurosurgical interventions)**	**High risk (for neurosurgical interventions)**
- GCS score <15 at 2 hrs after injury	- GCS score <15 at presentation
- Suspected open or depressed skull fracture	- Suspected open or depressed skull fracture
- Any sign of basal skull fracture	- Any sign of basal skull fracture
- Vomiting ≥ 2 episodes	- Vomiting ≥ 2 episodes
- Age ≥ 65 years	- Age ≥ 65 years
**Medium risk (for brain injury on CT)**	**Medium risk (for brain injury on CT)**
Amnesia before impact > 30 minutes	- Amnesia before impact > 30 minutes
Dangerous mechanism of injury (pedestrian struck by vehicle, ejection from vehicle, fall from >3 feet or >5 stairs.	Dangerous mechanism of injury (pedestrian struck by vehicle, ejection from vehicle, fall from >3 feet or >5 stairs.
* Patients with any positive high risk or medium risk criteria will be considered to have a 'positive' outcome (requiring transport to a hospital)	

Study duration and sample size

This is a feasibility study, in which evaluating recruitment rate is an outcome; therefore, it is not possible to specify a sample size a priori. However, we intend to recruit participants for a

For peer review only - http://bmjopen.bmj.com/site/about/guidelines.xhtml

fixed period of six months. Based on data routinely collected by the Manchester University NHS Foundation Trust, a total of 5,332 patients arrived in the ED by ambulance with a primary complaint of head injury over a period of four years and nine months up to November 2020. We intend to recruit participants from two similarly sized MTCs. We anticipate that approximately 1,100 patients will be eligible to participate in this study during the recruitment period. Accounting for the availability of trained paramedics on the study delegation log and concurrent injuries to other body systems that would exclude patients from participating in this study, approximately 100 participants can be recruited to this study. This number of participants is generally accepted as being appropriate for studies that focus on assessing feasibility and acceptability (20).

Outcomes

We will study the following feasibility outcomes:

- The number of eligible patients who are approached.
- The proportion of those approached who consent to participate in the study.
- The completeness of data collection.
- The completeness of follow-up.
- The number and proportion of patients with neurosurgically significant TBI.
- Determination of the inter-observer reliability of the CCHR completed by prehospital care providers and ED physicians.
- Determination of the inter-observer reliability for each component of the CCHR assessed by prehospital care providers and ED physicians.
- The diagnostic performance and clinical sensibility of the CCHR in the opinion of attending paramedics.
- Determine the acceptability of trial processes as perceived by paramedics and patients.

For peer review only

Measuring and defining outcomes

Feasibility outcomes will be assessed using screening logs and case report forms completed by attending paramedics during the course of the study. At the end of the study period, a minimum of two clinicians (affiliated with the relevant NHS trusts) will review in-hospital patient records to diagnose the secondary outcomes of this feasibility study (neurosurgically significant TBI, abnormal CT findings and death within seven days). A third independent investigator will be invited to adjudicate any discrepancies. Diagnostic adjudication will be blinded to the final interpretation of the CCHR. Additionally, a neuroradiology consultant/expert will be consulted in case of the CT scan diagnosis is inconclusive.

Neurosurgical interventions are defined as any neurosurgical intervention including intracranial pressure monitoring or an Abbreviated Injury Scale (AIS) with the severity $\geq$3 in the head region. Abnormal head CT findings will be classified as normal or abnormal and are defined as at least one of the following: skull fractures, intracranial lesions, such as cerebral contusion and traumatic intracranial haemorrhages, or other clinically significant findings requiring hospital admission, neurosurgical interventions, and routine follow-up.

Data collection

Prehospital setting

During the study period, all patients will receive standard prehospital care in accordance with local guidelines. Prior to their participation in the study, all participating paramedics will be provided with bespoke training specifically designed for the study. This training will cover all aspects of the study protocol, including eligibility assessment, the consent process, data collection procedures, and guidance on interpreting the CCHR. To ensure accessibility and

For peer review only

flexibility, the training will be offered both face-to-face and through various online sessions conducted via Zoom. Additionally, the training sessions have been recorded and made available online for those unable to attend live. This approach allows paramedics to access the training material at their convenience, ensuring that all participants receive comprehensive training on the study protocols and data collection methods.

Prehospital data will be collected using Research Electronic Data Capture (REDCap), a secure web-based platform hosted at the University of Manchester. REDCap is a software application that was created to facilitate the process of collecting and managing research data. Prior to recruitment, a training session will be held to equip participating sites with data collection skills. In addition, paper case report forms (CRFs) will be available as an alternative method of data collection. It is expected that this approach will provide flexibility and ensure continuity in data collection. A variety of data will be collected in the prehospital setting, including, but not limited to:

- Baseline demographics information (i.e, gender - age)
- Anticoagulant use.
- Injury characteristics (i.e., mechanism of injury, place).
- Past medical history.
- Initial chief complaint (i.e., vomiting, headache, loss of consciousness, amnesia).
- Prehospital physiological parameters (i.e., GCS, vital signs).
- Clinical criteria required to use the CCHR.
- Destination hospital.

Paramedics will record these data contemporaneously, either prior to or immediately following handover at hospital.

For peer review only

In-hospital environment

REDCap will be utilised to collect hospital data, and a paper CRF will also be available as an alternative. Emergency physicians at the receiving hospital will assess the patients to determine whether a CT scan is necessary in accordance with local and national guidelines. Relevant data about imaging studies undertaken and their findings will be extracted from hospital records. The routine radiology reports will be used to extract the follow-up data required for this study. Where there is uncertainty about the interpretation of the radiology report, a second radiologist will be asked to interpret the images.

Further, emergency physicians at the receiving hospitals will be asked to assess the patients using the CCHR and record their findings, blinded to the assessments made by participating paramedics. These data will be used to determine the inter-observer reliability of the CCHR between paramedics in the prehospital environment and clinicians in the ED. Additionally, in-hospital medical records will be reviewed to obtain information regarding neurosurgical interventions undertaken during the patient's hospital admission.

Follow-up

Following the initial presentation to the ED, patients will be followed up to the time of hospital discharge. Data will be collected from the clinical records of the patient, including, but not limited to

- GCS on arrival in hospital.
- Details of the clinician's assessment of the CCHR.
- Outcome of any brain imaging (i.e., CT and/or MR scans, recorded as part of routine practice).
- AIS in relation to head injury.

For peer review only

- Injury Severity Score.
- Mortality status.
- Neurosurgical procedures and/or surgery.

Evaluation of acceptability

We will use a qualitative approach to evaluate the acceptability of trial processes used to study participants. We will purposively sample (a) study participants and (b) paramedics. The intention is to maximise heterogeneity (according to age, sex and ethnic origin, and for paramedics, seniority and base ambulance station). Patients and participating paramedics will be interviewed for the purpose of exploring their experiences of participating in a study of this nature. Previous emergency care research has demonstrated the value of this method in providing information regarding the feasibility and design of a larger study (21).

To maximise convenience for participants and thereby recruitment, participants will be invited to take part in a semi-structured interview either by telephone, face-to-face or video conference. Informed consent will be obtained from all participants. We will explore the factors that facilitate or discourage participation in the research. Paramedics and study participants will be offered a £30 high street voucher to thank them for taking part. Telephone and sign language interpreters will be provided where necessary. The interview topic guides (Appendix 1) consist of a number of questions and prompts concerning the patients and paramedics' experiences of participating in this study, as well as the paramedics' perceptions of the viability of the CCHR. Prior to commencement of the interviews, two pilot interviews will be carried out. To determine the relevance, clarity and duration required to complete each interview, one will be with a paramedic and the other will be with a patient (22).

For peer review only

The Ottawa Acceptability of Decision Rules Instrument (OADRI) is a 12-item instrument that will be used to measure paramedics' attitudes toward the CCHR and clinical sensibility (23). Paramedics will be asked to provide their level of agreement with twelve statements about the CCHR using a six-point Likert scale. The first seven statements are worded in such a way that a higher number indicates higher acceptance, while the last five are worded in the opposite manner to minimise the acquiescence bias (24).

Consent process

An exception to the normal process of informed consent is established for patients in emergency situations who are unable to provide consent. As stated in the Declaration of Helsinki, "for a research subject who is legally incompetent, physically or mentally incapable of giving consent or is a legally incompetent minor, the investigator must obtain informed consent from the legally authorised representative in accordance with applicable law. These groups should not be included in research unless the research is necessary to promote the health of the population represented and this research cannot instead be performed on legally competent persons" (25).

In this study, we will include patients in the prehospital environment who are receiving an emergency ambulance response having sustained a head injury. The nature of this presentation means that mental capacity to provide informed consent for participation in research will be compromised, especially given the time critical nature of the emergency medical response. Therefore, we will adopt a deferred consent model, which is widely used in research of this nature (26). Deferred consent in the emergency setting is a practice where medical treatment or research participation is initiated without prior consent from patients due to urgent

For peer review only

circumstances and their compromised mental capacity, with the intention to obtain consent retrospectively once the patient's mental capacity has improved (27).

Paramedics will enrol eligible participants, enabling them to collect the required data. Patient care will be unchanged. We will then seek deferred written informed consent after the patients have received their initial treatment in hospital, and once they have regained their mental capacity.

When seeking written informed consent, a participant information sheet will be provided. Deferred consent may be obtained by either of the following means: (a) a member of staff may seek written informed consent at the receiving hospital; or (b) deferred consent may be sought in the community by post (written informed consent), email or telephone. While seeking deferred consent, staff will be available to answer any questions that the participants may have.

For participants lacking capacity, we will seek consent from their relative or legally authorised representative, which is known as proxy consent (28). A research nurse will briefly explain to them that the patient will receive the usual hospital care for TBI. The responsible nurse will also clarify that only basic clinical data will be reported using a case report form to be analysed to improve prehospital care. A brief information sheet will be provided upon request. The ability of participants to provide consent can be assessed at any point during the study period. Participants who regain capacity will be provided with a detailed participant information sheet and their written consent to continue in this feasibility study will be obtained. If the patient does not regain capacity, we will seek deferred assent from a relative or professional legal representative to retain the patient’s data in the study.

For peer review only

Data analysis

Quantitative data

A descriptive analysis will be conducted based on the baseline data for patients enrolled in the study to characterise the demographic and clinical profile. This will include the number of eligible participants who were approached, the proportion that consented to participate, data completeness (expressed as number and percentage) for each variable collected and number and percentage of participants completing follow-up. We will also report recruitment data as rates (number of participants enrolled per week across the study period).

The inter-observer reliability between paramedics and emergency physicians' use of the CCHR will be calculated using Cohen's Kappa, a statistical method for measuring agreement between two observers, together with 95% confidence intervals. Additionally, we will report upon the CCHR's diagnostic performance for ruling out clinically significant TBI. This will be achieved by calculating the sensitivity, specificity, positive and negative likelihood ratios with 95% confidence intervals against a reference standard of traumatic intracranial lesion, which will be identified from in-hospital records. Given the nature of this feasibility study, the analyses will be viewed as exploratory, preliminary and will be descriptive in nature. Furthermore, to determine the acceptability of the CCHR, the OADRI average item scores will be calculated, with higher scores indicating greater acceptability.

Qualitative data

The six-stage thematic analysis approach will be employed to analyse all interviews, as suggested by Braun and Clarke (29). This involves becoming familiar with the data, generating initial codes, searching for themes, reviewing themes, defining and naming themes and sub-themes, and producing the report. We will adhere to the four criteria for trustworthiness

established by Lincoln and Guba: credibility, dependability, confirmability and transferability (30). Each step of the qualitative data collection and analysis will be guided by a qualitative research expert. The qualitative data will be managed and analysed using NVivo 12.

Progression criteria

This feasibility study will be overseen and governed by the Trial Steering Committee (TSC), which will include the chief investigator, principal investigator, two lay members, topic experts in TBI and one paramedic. The role of the TSC is to provide external oversight and to ensure that the study is conducted in compliance with Good Clinical Practice guidelines (31, 32).

The traffic light approach (green/amber/red), also known as a (stop-amend-go) rule, will be used to determine whether or not to proceed with a full definitive trial (33). Green (go) indicates that the study meets the pre-set criteria, and that it would be feasible for a full-powered definitive trial to be conducted without any modifications to the study protocol. Amber (amend) indicates that further feasibility work or modifications to the protocol are required before proceeding with the full trial. Red (stop) indicates that proceeding to a definitive study is not recommended. Taking into account the feasibility outcomes, as well as qualitative interviews with patients and paramedics, a decision will be made regarding whether or not to proceed with the trial or whether to make any amendments to the study design. The progression criteria are shown in Table 2.

Patient and public involvement

It has become increasingly apparent that involving patients and the public in healthcare research can enhance the development and implementation of clinical studies, the recruitment of participants, and the relevance of study topics and outcomes in relation to their needs and experiences (34, 35). It will be a priority to take into account the perspectives of patients and

For peer review only

the public during the design, conduct, reporting and dissemination of the research. To ensure maximum clinical impact, the study has been discussed with patient and public representatives through the NIHR Brain Injury MedTech Co-operative. A comprehensive and in-depth discussion was conducted regarding the study procedures, including key aspects such as the consent process and patient-facing materials. Two members have agreed to join the study steering committee, which is expected to meet two or three times during the study period to advise on the progress of the research, discuss any adverse events, proposed protocol amendments and other matters relating to research governance. Additionally, a lay summary will be developed in collaboration with the lay members to disseminate the study findings.

Table 2: Progression criteria to proceed with a definitive trial.

	Contributing data	Green (Proceed to a definitive trial)	Amber (Proceed to a definitive trial following some changes to the protocol	Do not proceed to a definitive trial
**Screening**	Training and delegation logs	At least 20 paramedics at participating ambulance hubs trained and on delegation logs	At least 10 paramedics at participating ambulance hubs trained and on delegation logs	Less than 10 paramedics at participating ambulance hubs trained and on delegation logs
**Recruitment acceptability**	Screening & recruitment log	≥ 80% of eligible screened patients are consented to take part	≥ 60% of eligible screened patients are consented to take part	< 60% of eligible screened patients are consented to take part
**Retention**	Screening & recruitment log	Retention of ≥ 90% of study participants until study completion	Retention of ≥ 80% of study participants until study completion	Retention of < 80% of study participants until study completion
**Study procedures**	Participation data	At least 85% of the outcome data is collected (e.g., case report forms)	A minimum of 60% of the outcome data is collected (e.g., case report forms)	< 60% of the outcome data is collected (e.g., case report forms)
**Adverse events**	Participation data	No or very minor adverse events and no participants discontinued the study	Minor or serious adverse events leading to 10% or less participants discontinuing the study	Serious adverse events leading to >10% of participants discontinuing the study

For peer review only

**Acceptability**	Participant interview Paramedics questionnaire and interviews	Responses to OADRI* all ‘agree’ or ‘strongly agree’. No major barriers to acceptability/participation identified during interviews with paramedics and patients	Responses to OADRI mainly ‘agree’ or ‘strongly agree’. Some barriers to participation identified during interviews but remediable action identified.	Responses to OADRI mainly ‘disagree’ or ‘strongly disagree’. Major barriers to acceptability/participation identified during interviews with no clear remediable action.

* OADRI: Ottawa Acceptability of Decision Rules Instrument

Safety considerations, adverse events and serious adverse events

Given the nature of this study, any risk to participants is very low. It is important to note that this study is observational in nature: a patient’s participation in this study will have no effect on the quality of medical care they usually receive in both prehospital and hospital settings. Our study will have no impact on the decision to undergo a CT scan as this will be undertaken at the discretion of the treating clinician, as part of routine clinical care, in accordance with current national guidance. The study will also have no effect on the decision to transport patients to the hospital. There are also small risks associated with data management, but we have carefully considered how data will be handled during the study and we will operate in accordance with current legislation and the standard operating procedures of the sponsor organisation to mitigate that risk. We are not aware of any potential risks this study would pose to the researchers.

Because of the low-risk nature of the study, we do not anticipate any adverse events (including serious adverse events) as a result of participating in this research. However, given the patient group (patients with TBI), we anticipate that many patients will experience expected adverse events, which are related to their initial injury. We will not ask sites to report expected adverse events. Expected serious adverse events include, for example, death due to head injury;

For peer review only

neurosurgical procedures for TBI; admission to hospital for traumatic brain injury; loss of function due to TBI. Unexpected adverse events and serious adverse events should be reported. Adverse events will be defined in accordance with ICH Good Clinical Practice as any untoward medical occurrence in a participant, but will only be reported if this was not expected as a result of the patient's condition, up to the point of handover in hospital.

Impact

It is anticipated that the feasibility study will provide valuable insight into whether conducting a full trial in the future is warranted. It will also provide guidance to enhance the study design, recruitment plan, and consent process. If feasibility is established, we will prepare to run a fully powered multi-centre study to evaluate the diagnostic accuracy of using the CCHR in the prehospital field. This will also involve drafting a proposal to apply for research funding.

The outcomes relating to diagnostic accuracy will be of particular relevance given our knowledge that this is the first study investigating the feasibility of using the CCHR in the prehospital environment. Therefore, it is anticipated that the findings of this feasibility study will be used to inform design of a fully powered diagnostic test accuracy study. The findings of this study will be complemented by the findings of mixed methods research that we are also conducting which will explore facilitators and barriers to implementing new diagnostic pathways in the prehospital environment as perceived by practising paramedics (36).

In parallel with a fully powered diagnostic test accuracy study, it will also be important to work with key stakeholders including primary care practitioners, neurosurgeons and patient and public representatives. The care pathway for patients who are not conveyed to hospital will

For peer review only

need to be defined to ensure that patients are adequately supported with appropriate safety netting for those who experience ongoing symptoms or complications.

Ethics and dissemination

Ethical approval to conduct this feasibility study has been obtained from the Health Research Authority and the Research Ethics Committee (REC reference: 22/NW/0358). Any substantial amendment to the study protocol will need to be approved by an ethics committee after being approved by the sponsor. To ensure privacy and confidentiality, all records and files relating to the study will be kept in a secure location and will only be accessible to members of the research team. The results will be published in peer-reviewed journals, presented at relevant national and international conferences, and will be incorporated into a doctoral thesis (NA). The study findings will also be disseminated through presentations at relevant academic forums.

Declarations

Ethics approval and consent to participate

The nature of this study requires ethical approval from the Health Research Authority and Research Ethics Committee, which has been obtained (REC reference: 22/NW/0358).

Consent for publication

Not applicable.

Availability of data and material

Not applicable.

Competing interests

Richard Body has consulted for Abbott Point of Care and is undertaking contract clinical research with BrainBox, which manufactures assays for biomarkers of TBI.

Funding

This work is supported by a PhD scholarship awarded to NA by the Saudi Arabian Cultural Bureau in the UK and King Saud University (KSU1872). The funders had no role in the design of this study protocol. The study is sponsored by the University of Manchester. The publication fee is funded by the cardiovascular department, University of Manchester, UK. No grant or award number.

Authors contributions

NA collaborated with the study team (SB, SC, FL, and RB) in preparing the initial study protocol. The protocol was developed through collective input and discussions. NA took the lead in drafting the manuscript, working closely with SB, SC, FL, and RB to ensure its accuracy and completeness. The final version of the manuscript was approved by all authors.

Acknowledgements

Not applicable.

Author affiliations

[1]Division of Cardiovascular Sciences, University of Manchester, Manchester, UK. [2]Department of Accidents and Trauma, Prince Sultan bin Abdulaziz College for Emergency Medical Services, King Saud University, Riyadh, Saudi Arabia. [3]Medical Directorate, North West Ambulance Service NHS Trust, Bolton, BL1 5DD, UK. [4] Emergency Department, Manchester University NHS Foundation Trust, Manchester, UK. [5]University of Sheffield, School of Health and Related Research, Sheffield, UK.

For peer review only

References

1. Berkner J, Mannix R, Qiu J. Clinical Traumatic Brain Injury in the Preclinical Setting. Methods Mol Biol. 2016;1462:11-28.
2. Majdan M, Plancikova D, Brazinova A, Rusnak M, Nieboer D, Feigin V, et al. Epidemiology of traumatic brain injuries in Europe: a cross-sectional analysis. Lancet Public Health. 2016;1(2):e76-e83.
3. Anandalwar SP, Mau CY, Gordhan CG, Majmundar N, Meleis A, Prestigiacomo CJ, et al. Eliminating unnecessary routine head CT scanning in neurologically intact mild traumatic brain injury patients: implementation and evaluation of a new protocol. J Neurosurg. 2016;125(3):667-73.
4. Decuypere M, Klimo P, Jr. Spectrum of traumatic brain injury from mild to severe. Surg Clin North Am. 2012;92(4):939-57, ix.
5. Haydel MJ, Preston CA, Mills TJ, Luber S, Blaudeau E, DeBlieux PM. Indications for computed tomography in patients with minor head injury. N Engl J Med. 2000;343(2):100-5.
6. Stiell IG, Lesiuk H, Wells GA, McKnight RD, Brison R, Clement C, et al. The Canadian CT Head Rule Study for patients with minor head injury: rationale, objectives, and methodology for phase I (derivation). Ann Emerg Med. 2001;38(2):160-9.
7. Stiell IG, Lesiuk H, Wells GA, Coyle D, McKnight RD, Brison R, et al. Canadian CT head rule study for patients with minor head injury: methodology for phase II (validation and economic analysis). Ann Emerg Med. 2001;38(3):317-22.
8. Stiell IG, Clement CM, Rowe BH, Schull MJ, Brison R, Cass D, et al. Comparison of the Canadian CT Head Rule and the New Orleans Criteria in patients with minor head injury. Jama. 2005;294(12):1511-8.
9. Papa L, Stiell IG, Clement CM, Pawlowicz A, Wolfram A, Braga C, et al. Performance of the Canadian CT Head Rule and the New Orleans Criteria for predicting any traumatic intracranial injury on computed tomography in a United States Level I trauma center. Acad Emerg Med. 2012;19(1):2-10.
10. Bouida W, Marghli S, Souissi S, Ksibi H, Methammem M, Haguiga H, et al. Prediction value of the Canadian CT head rule and the New Orleans criteria for positive head CT scan and acute neurosurgical procedures in minor head trauma: a multicenter external validation study. Ann Emerg Med. 2013;61(5):521-7.
11. Bonney PA, Briggs A, Briggs RG, Jarvis CA, Attenello F, Giannotta SL. Rate of Intracranial Hemorrhage After Minor Head Injury. Cureus. 2020;12(9):e10653.
12. National Clinical Guideline C. National Institute for Health and Clinical Excellence: Guidance. Head Injury: Triage, Assessment, Investigation and Early Management of Head Injury in Children, Young People and Adults. London: National Institute for Health and Care Excellence (UK)
Copyright © National Clinical Guideline Centre, 2023.; 2023.
13. Pandor A, Goodacre S, Harnan S, Holmes M, Pickering A, Fitzgerald P, et al. Diagnostic management strategies for adults and children with minor head injury: a systematic review and an economic evaluation. Health Technol Assess. 2011;15(27):1-202.
14. Sultan HY, Boyle A, Pereira M, Antoun N, Maimaris C. Application of the Canadian CT head rules in managing minor head injuries in a UK emergency department: implications for the implementation of the NICE guidelines. Emergency Medicine Journal. 2004;21(4):420.

For peer review only

15. Hassan Z, Smith M, Littlewood S, Bouamra O, Hughes D, Biggin C, et al. Head injuries: a study evaluating the impact of the NICE head injury guidelines. Emergency medicine journal. 2005;22(12):845-9.
16. Lawrence T, Helmy A, Bouamra O, Woodford M, Lecky F, Hutchinson PJ. Traumatic brain injury in England and Wales: prospective audit of epidemiology, complications and standardised mortality. BMJ Open. 2016;6(11):e012197.
17. O'Cathain A, Knowles E, Bishop-Edwards L, Coster J, Crum A, Jacques R, et al. Understanding variation in ambulance service non-conveyance rates: a mixed methods study. Health Services and Delivery Research. 2018.
18. Mason S, Kuczawski M, Teare MD, Stevenson M, Goodacre S, Ramlakhan S, et al. AHEAD study: an observational study of the management of anticoagulated patients who suffer head injury. BMJ open. 2017;7(1):e014324.
19. Arain M, Campbell MJ, Cooper CL, Lancaster GA. What is a pilot or feasibility study? A review of current practice and editorial policy. BMC medical research methodology. 2010;10(1):1-7.
20. Teresi JA, Yu X, Stewart AL, Hays RD. Guidelines for Designing and Evaluating Feasibility Pilot Studies. Med Care. 2022;60(1):95-103.
21. van den Berg P, Kendal S, Alderson HV, Body R. An exploration of patients' experiences of participation in a randomised controlled trial of the Manchester Acute Coronary Syndromes (MACS) decision rule. Emerg Med J. 2017;34(9):593-8.
22. Majid MAA, Othman M, Mohamad SF, Lim SAH, Yusof A. Piloting for interviews in qualitative research: Operationalization and lessons learnt. International Journal of Academic Research in Business and Social Sciences. 2017;7(4):1073-80.
23. Brehaut JC, Graham ID, Wood TJ, Taljaard M, Eagles D, Lott A, et al. Measuring acceptability of clinical decision rules: validation of the Ottawa acceptability of decision rules instrument (OADRI) in four countries. Medical Decision Making. 2010;30(3):398-408.
24. Schriesheim C, Hill K. Controlling Acquiescence Response Bias by Item Reversals: The Effect on Questionnaire Validity. Educational and Psychological Measurement - EDUC PSYCHOL MEAS. 1981;41:1101-14.
25. Association WM. World Medical Association Declaration of Helsinki: ethical principles for medical research involving human subjects. Jama. 2013;310(20):2191-4.
26. Kompanje E, Jansen T, Le Noble J, de Geus H, Bakker J. Deferred consent for inclusion of patients unable to give their consent in studies in the field of emergency medicine. Nederlands Tijdschrift Voor Geneeskunde. 2008;152(38):2057-61.
27. van der Graaf R, Hoogerwerf MA, de Vries MC. The ethics of deferred consent in times of pandemics. Nat Med. 2020;26(9):1328-30.
28. Kompanje EJO, Maas AIR, Hilhorst M, Slieker F, Teasdale G. Ethical considerations on consent procedures for emergency research in severe and moderate traumatic brain injury. Acta Neurochirurgica. 2005;147:633-40.
29. Braun V, Clarke V. Using thematic analysis in psychology. Qualitative research in psychology. 2006;3(2):77-101.
30. Forero R, Nahidi S, De Costa J, Mohsin M, Fitzgerald G, Gibson N, et al. Application of four-dimension criteria to assess rigour of qualitative research in emergency medicine. BMC Health Serv Res. 2018;18(1):120.
31. Daykin A, Selman L, Cramer H, McCann S, Shorter G, Sydes M, et al. What are the roles and valued attributes of a Trial Steering Committee? Ethnographic study of eight clinical trials facing challenges. Trials. 2016;17:307.

For peer review only

32. Harman NL, Conroy EJ, Lewis SC, Murray G, Norrie J, Sydes MR, et al. Exploring the role and function of trial steering committees: results of an expert panel meeting. Trials. 2015;16:597.
33. Avery KNL, Williamson PR, Gamble C, Connell Francischetto E, Metcalfe C, Davidson P, et al. Informing efficient randomised controlled trials: exploration of challenges in developing progression criteria for internal pilot studies. BMJ Open. 2017;7(2):e013537.
34. Price A, Albarqouni L, Kirkpatrick J, Clarke M, Liew SM, Roberts N, et al. Patient and public involvement in the design of clinical trials: an overview of systematic reviews. Journal of evaluation in clinical practice. 2018;24(1):240-53.
35. Gamble C, Dudley L, Allam A, Bell P, Goodare H, Hanley B, et al. Patient and public involvement in the early stages of clinical trial development: A systematic cohort investigation. BMJ open. 2014;4:e005234.
36. Alqurashi N, Alotaibi A, Bell S, Lecky F, Body R. Towards exploring current challenges and future opportunities relating to the prehospital triage of patients with traumatic brain injury: a mixed-methods study protocol. BMJ Open. 2023;13(3):e068555.

Figure legends:

- **Figure 1: Study flowchart**

For peer review only

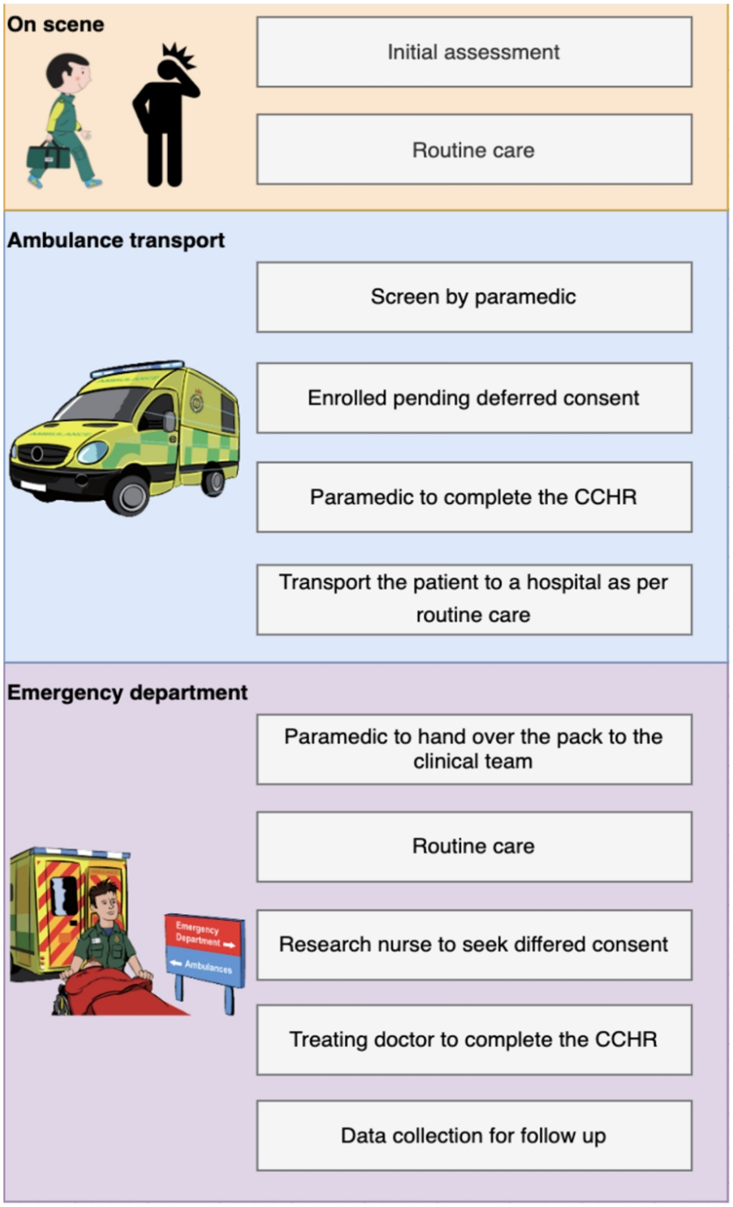

Figure 1: Study flowchart

149x247mm (144 x 144 DPI)

Head injury Evaluation and Ambulance Diagnosis: A Feasibility Study

Semi-structured interview, topic guide – Patients

Date: **Participant ID number:**

Pre-interview

- Privacy notice read.
- Consent form.
- Information sheet.

---

1. Please share your experience from the moment you were initially approached about the study.
   - **Follow-up questions, if required:**

   a. How did you feel about being approached for the study? Were there any specific factors that influenced your decision to participate?

   b. Were you given enough information to help you to decide whether to take part in this study? Was there anything that you found confusing or unclear?

   c. In this study, we didn't ask patients for permission during the ambulance journey. This is because they had experienced a head injury and were being taken to hospital as an emergency. How did you feel about this? Do you think you would have been able to make a decision about taking part when you were still in the ambulance?

2. How did you feel about the way we asked for your permission to take part in this study?
   - **Follow-up questions, if required:**

   a. Please share your overall thoughts about how we asked for your permission to take part in this study.

   b. Is there anything particularly good or bad about the way we asked for your permission to take part in the study?

   c. Do you think you were given enough time to decide whether to take part? Why or why not?

3. Can you recall any specific reasons or factors that influenced your belief that it is important to understand the study? How did this understanding affect your decision to take part in this study?

For peer review only - http://bmjopen.bmj.com/site/about/guidelines.xhtml

4. In your opinion, are there any factors that you believe might discourage people from participating in this study?

Follow-up questions, if required:

   a. Is there anything that might discourage people from participating in this study? How do you think these factors could be addressed?

5. Can you share any unexpected events or experiences that occurred during your participation in the study?

6. Would you recommend taking part in this trial to somebody else?

Follow-up questions, if required:

   a. Can you explain your thoughts and the reasons behind your recommendation or non-recommendation of participation in this study to others?
   b. In your opinion, what specific factors or aspects of the study influenced your recommendation or non-recommendation?
   c. Is there anything that you think would stop people from fully participating in this study?

7. Is there anything else you would still like to know about this study?

******END******

For peer review only - http://bmjopen.bmj.com/site/about/guidelines.xhtml

Head injury Evaluation and Ambulance Diagnosis: A Feasibility Study
Semi-structured interview, topic guide - Paramedics

Date: **Participant ID number:**

Pre-interview

- Privacy notice read.
- Consent form.
- Information sheet.

---

1. Firstly, I would like to know how long you have been a paramedic, and what is your highest academic qualification level?

2. Have you completed the training on the study protocol?
   a. How did you find it?
   b. Did you find the study protocol easy to understand?
   c. Was the protocol easy to remember in practice?
   d. What could be improved in the trial protocol?
3. What was the best aspect of being involved in the study?
   a. Could you tell me how easy it was to use the study case report form?

4. What would be the advantages of using the Canadian CT head Rule in the prehospital setting?
   a. Looking back to your experience, could you tell me how did you find using the Canadian CT head rule in the prehospital field?

5. What would be the disadvantages of using the Canadian CT head Rule in the prehospital setting?
   a. Looking back to your experience, did you experience any barriers/challenges to applying the Canadian CT Head Rule?

6. Would you like the Canadian CT Head Rule to become part of your practice?

7. Do you have any additional thoughts you would like to share about the study?

END

For peer review only - http://bmjopen.bmj.com/site/about/guidelines.xhtml

SPIRIT Checklist

		Reporting Item	Page Number
**Administrative information**			
Title	#1	Descriptive title identifying the study design, population, interventions, and, if applicable, trial acronym	1
Trial registration	#2a	Trial identifier and registry name. If not yet registered, name of intended registry	8
Trial registration: data set	#2b	All items from the World Health Organization Trial Registration Data Set	NA
Protocol version	#3	Date and version identifier	NA
Funding	#4	Sources and types of financial, material, and other support	24
Roles and responsibilities: contributorship	#5a	Names, affiliations, and roles of protocol contributors	24,25
Roles and responsibilities: sponsor contact information	#5b	Name and contact information for the trial sponsor	24
Roles and responsibilities: sponsor and funder	#5c	Role of study sponsor and funders, if any, in study design; collection, management, analysis, and interpretation of data; writing of the report; and the decision to submit the report for publication, including whether they will have ultimate authority over any of these activities	24
Roles and responsibilities: committees	#5d	Composition, roles, and responsibilities of the coordinating centre, steering committee, endpoint adjudication committee, data management team, and other individuals or groups overseeing the trial, if applicable (see Item 21a for data monitoring committee)	19
**Introduction**			

For peer review only

Background and rationale	#6a	Description of research question and justification for undertaking the trial, including summary of relevant studies (published and unpublished) examining benefits and harms for each intervention	5-7
Background and rationale: choice of comparators	#6b	Explanation for choice of comparators	NA
Objectives	#7	Specific objectives or hypotheses	8
Trial design	#8	Description of trial design including type of trial (eg, parallel group, crossover, factorial, single group), allocation ratio, and framework (eg, superiority, equivalence, non-inferiority, exploratory)	8,9
**Methods: Participants, interventions, and outcomes**			
Study setting	#9	Description of study settings (eg, community clinic, academic hospital) and list of countries where data will be collected. Reference to where list of study sites can be obtained	8,9
Eligibility criteria	#10	Inclusion and exclusion criteria for participants. If applicable, eligibility criteria for study centres and individuals who will perform the interventions (eg, surgeons, psychotherapists)	9,10
Interventions: description	#11a	Interventions for each group with sufficient detail to allow replication, including how and when they will be administered	NA
Interventions: modifications	#11b	Criteria for discontinuing or modifying allocated interventions for a given trial participant (eg, drug dose change in response to harms, participant request, or improving / worsening disease)	NA
Interventions: adherance	#11c	Strategies to improve adherence to intervention protocols, and any procedures for monitoring adherence (eg, drug tablet return; laboratory tests)	13-15
Interventions: concomitant care	#11d	Relevant concomitant care and interventions that are permitted or prohibited during the trial	NA
Outcomes	#12	Primary, secondary, and other outcomes, including the specific measurement variable (eg, systolic blood pressure), analysis	11,12

For peer review only

		metric (eg, change from baseline, final value, time to event), method of aggregation (eg, median, proportion), and time point for each outcome. Explanation of the clinical relevance of chosen efficacy and harm outcomes is strongly recommended	
Participant timeline	#13	Time schedule of enrolment, interventions (including any run-ins and washouts), assessments, and visits for participants. A schematic diagram is highly recommended (see Figure)	13-15 and Figure 1
Sample size	#14	Estimated number of participants needed to achieve study objectives and how it was determined, including clinical and statistical assumptions supporting any sample size calculations	11
Recruitment	#15	Strategies for achieving adequate participant enrolment to reach target sample size	11
**Methods: Assignment of interventions (for controlled trials)**			
Allocation: sequence generation	#16a	Method of generating the allocation sequence (eg, computer-generated random numbers), and list of any factors for stratification. To reduce predictability of a random sequence, details of any planned restriction (eg, blocking) should be provided in a separate document that is unavailable to those who enrol participants or assign interventions	NA
Allocation concealment mechanism	#16b	Mechanism of implementing the allocation sequence (eg, central telephone; sequentially numbered, opaque, sealed envelopes), describing any steps to conceal the sequence until interventions are assigned	NA
Allocation: implementation	#16c	Who will generate the allocation sequence, who will enrol participants, and who will assign participants to interventions	NA
Blinding (masking)	#17a	Who will be blinded after assignment to interventions (eg, trial participants, care providers, outcome assessors, data analysts), and how	NA
Blinding (masking): emergency unblinding	#17b	If blinded, circumstances under which unblinding is permissible, and procedure for revealing a participant's allocated intervention during the trial	NA
**Methods: Data**			

For peer review only

**collection, management, and analysis**			
Data collection plan	#18a	Plans for assessment and collection of outcome, baseline, and other trial data, including any related processes to promote data quality (eg, duplicate measurements, training of assessors) and a description of study instruments (eg, questionnaires, laboratory tests) along with their reliability and validity, if known. Reference to where data collection forms can be found, if not in the protocol	13-15
Data collection plan: retention	#18b	Plans to promote participant retention and complete follow-up, including list of any outcome data to be collected for participants who discontinue or deviate from intervention protocols	15
Data management	#19	Plans for data entry, coding, security, and storage, including any related processes to promote data quality (eg, double data entry; range checks for data values). Reference to where details of data management procedures can be found, if not in the protocol	13,14
Statistics: outcomes	#20a	Statistical methods for analysing primary and secondary outcomes. Reference to where other details of the statistical analysis plan can be found, if not in the protocol	18,19
Statistics: additional analyses	#20b	Methods for any additional analyses (eg, subgroup and adjusted analyses)	NA
Statistics: analysis population and missing data	#20c	Definition of analysis population relating to protocol non-adherence (eg, as randomised analysis), and any statistical methods to handle missing data (eg, multiple imputation)	18,19
**Methods: Monitoring**			
Data monitoring: formal committee	#21a	Composition of data monitoring committee (DMC); summary of its role and reporting structure; statement of whether it is independent from the sponsor and competing interests; and reference to where further details about its charter can be found, if not in the protocol. Alternatively, an explanation of why a DMC is not needed	19,20
Data monitoring: interim analysis	#21b	Description of any interim analyses and stopping guidelines, including who will have access to these interim results and make the final decision to terminate the trial	19,20

For peer review only

Harms	#22	Plans for collecting, assessing, reporting, and managing solicited and spontaneously reported adverse events and other unintended effects of trial interventions or trial conduct	21,22
Auditing	#23	Frequency and procedures for auditing trial conduct, if any, and whether the process will be independent from investigators and the sponsor	19
**Ethics and dissemination**			
Research ethics approval	#24	Plans for seeking research ethics committee / institutional review board (REC / IRB) approval	23
Protocol amendments	#25	Plans for communicating important protocol modifications (eg, changes to eligibility criteria, outcomes, analyses) to relevant parties (eg, investigators, REC / IRBs, trial participants, trial registries, journals, regulators)	23
Consent or assent	#26a	Who will obtain informed consent or assent from potential trial participants or authorised surrogates, and how (see Item 32)	16-18
Consent or assent: ancillary studies	#26b	Additional consent provisions for collection and use of participant data and biological specimens in ancillary studies, if applicable	NA
Confidentiality	#27	How personal information about potential and enrolled participants will be collected, shared, and maintained in order to protect confidentiality before, during, and after the trial	23,24
Declaration of interests	#28	Financial and other competing interests for principal investigators for the overall trial and each study site	24
Data access	#29	Statement of who will have access to the final trial dataset, and disclosure of contractual agreements that limit such access for investigators	24
Ancillary and post trial care	#30	Provisions, if any, for ancillary and post-trial care, and for compensation to those who suffer harm from trial participation	NA
Dissemination policy: trial results	#31a	Plans for investigators and sponsor to communicate trial results to participants, healthcare professionals, the public, and other relevant groups (eg, via publication, reporting in results databases, or other data sharing arrangements), including any publication restrictions	23,24

For peer review only

Dissemination policy: authorship	#31b	Authorship eligibility guidelines and any intended use of professional writers	NA
Dissemination policy: reproducible research	#31c	Plans, if any, for granting public access to the full protocol, participant-level dataset, and statistical code	NA
**Appendices**			
Informed consent materials	#32	Model consent form and other related documentation given to participants and authorised surrogates	
Biological specimens	#33	Plans for collection, laboratory evaluation, and storage of biological specimens for genetic or molecular analysis in the current trial and for future use in ancillary studies, if applicable	NA

For peer review only
